# Secondary metabolome and its defensive role in the aeolidoidean *Phyllodesmium longicirrum*, (Gastropoda, Heterobranchia, Nudibranchia)

**DOI:** 10.3762/bjoc.13.50

**Published:** 2017-03-13

**Authors:** Alexander Bogdanov, Cora Hertzer, Stefan Kehraus, Samuel Nietzer, Sven Rohde, Peter J Schupp, Heike Wägele, Gabriele M König

**Affiliations:** 1 Institute for Pharmaceutical Biology, University of Bonn, Nussallee 6, 53115 Bonn, Germany; 2 Institute for Chemistry and Biology of the Marine Environment (ICBM), Carl-von-Ossietzki University Oldenburg, Schleusenstraße 1, 26382 Wilhelmshaven, Germany; 3 Zoologisches Forschungsmuseum Alexander Koenig, Adenauerallee 160, 53113 Bonn, Germany

**Keywords:** chemical defense, chemoecology, natural compounds, Nudibranchia, *Phyllodesmium longicirrum*

## Abstract

*Phyllodesmium longicirrum* is the largest aeolidoidean species known to date, and extremely rich in terpenoid chemistry. Herein we report the isolation of a total of 19 secondary metabolites from a single specimen of this species, i.e., steroids **1–4**, cembranoid diterpenes **5–13**, complex biscembranoids **14** and **15**, and the chatancin-type diterpenes **16–19**. These compounds resemble those from soft corals of the genus *Sarcophyton*, of which to date, however, only *S. trocheliophorum* is described as a food source for *P. longicirrum*. Fish feeding deterrent activity was determined using the tropical puffer fish *Canthigaster solandri*, and showed activity for (2*S*)-isosarcophytoxide (**10**), cembranoid bisepoxide **12** and 4-oxochatancin (**16**). Determining the metabolome of *P. longicirrum* and its bioactivity, makes it evident that this seemingly vulnerable soft bodied animal is well protected from fish by its chemical arsenal.

## Introduction

Marine gastropods, of which approximately 150.000 are known, mostly are protected by a shell. However, shell reduction or even loss is common within several marine Heterobranchia clades, which were united under the name Opisthobranchia in former times [1]. To compensate this lack of physical protection, alternative defensive strategies, such as the production of calcareous needles or acidic sulfates, and sequestration or de novo synthesis of toxic metabolites emerged within the opisthobranch taxa [2–5]. Adaptations and mimicry, which help to hide in habitats is frequent in marine gastropods, as obvious from very diverse and spectacular phenotypes [1,6].

A most intriguing defense strategy, the incorporation of intact stinging cells (cnidocysts) from hydrozoan food sources, is used by animals belonging to the Aeolidoidea [7]. One of the aeolidoidean genera, i.e., *Phyllodesmium*, however, switched to cnidocyst poor Octocorallia as a food source. From there, some *Phyllodesmium* species, including *P. longicirrum* incorporate algal unicellular symbionts. These so-called zooxanthellae are stored in branches of the digestive glands which reach into the so-called cerata. *P. longicirrum* is able to maintain the symbionts for over 6 months and is believed to benefit from additional nutrients produced via photosynthesis [8–9]. The greenish-brownish color of the dinoflagellates offers additional camouflage, while being exposed grazing on soft coral surfaces. Most importantly, the octocorallian food also offers a wide spectrum of terpenoid chemistry, which is incorporated and stored by *Phyllodesmium*.

Only few chemical investigations were undertaken on *Phyllodesmium* species [10–15], describing mostly terpenoid secondary metabolites. Indirect evidence suggests that these compounds are sequestered from the respective octocorallian prey organisms. In rare cases, the ecological function of some of these metabolites as deterrent agents was demonstrated, e.g., acetoxypukalide from *P. guamensis* [15] and 4-oxochatancin (**16**) in *P. longicirrum* [12] were shown to cause a significant feeding deterrence under laboratory conditions at concentration levels below natural abundance in the sea slug bodies.

Herein we report on the secondary metabolome of a single specimen of *P. longicirrum*, including the structure elucidation of the new metabolites **1**, **5**, **9**, **14** and **15**, and show the fish feeding deterrent activity of the major metabolites **10** and **12**.

## Results

### UPLC–HRMS metabolome analysis

From the ethanolic extract of *P. longicirrum* the ethyl acetate-soluble organic compounds were analyzed. A first fractionation was achieved by vacuum liquid chromatography (VLC) on reversed-phase material yielding 11 fractions. ^1^H NMR analysis of these indicated the presence of chemically diverse secondary metabolites in the major fractions 3–8, whereas the hydrophilic fractions 1 and 2 merely contained sugars and the lipophilic ones, i.e., 9–11 simple lipids.

**Figure 1:**
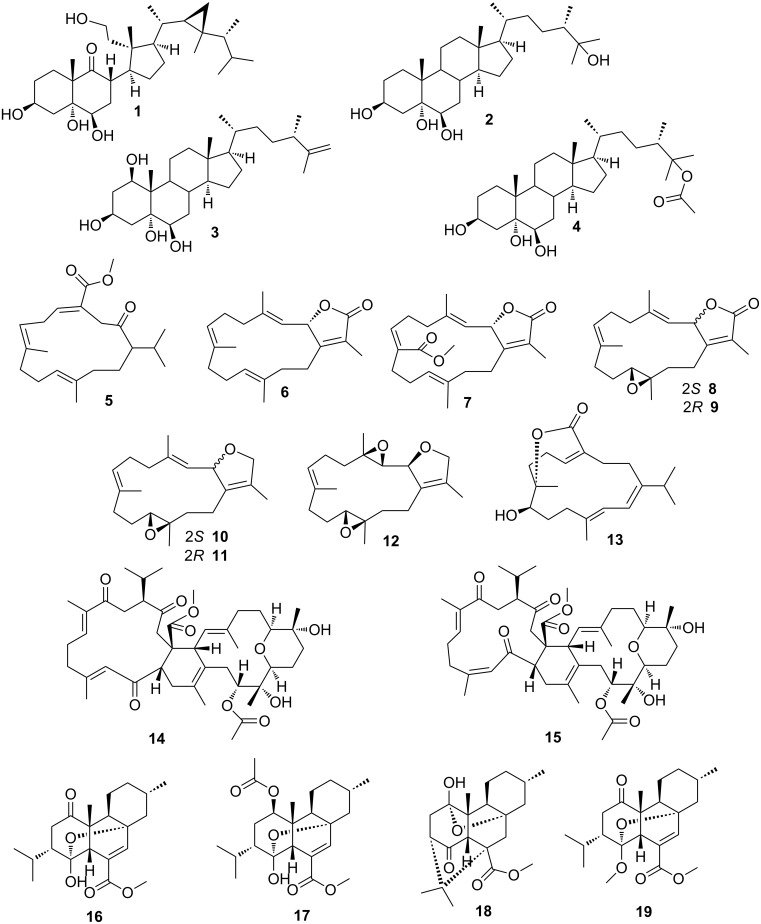
Secondary metabolites isolated in this study from *P. longicirrum*.

Detailed UPLC–HRMS investigation was thus performed with the VLC fractions 3–8. The resulting UPLC chromatograms (Supporting Information File 1, Figures S47–52) were extremely complex and gave an impression on the multi-faceted metabolome of this animal. The majority (except **4** and **17**) of the subsequently isolated and characterized secondary metabolites (**1**–**19**, Figure 1) could be assigned to the detected *m*/*z* values (Supporting Information File 1, Table S7A), e.g., prominent MS data were associated with the presence of metabolites with a molecular weight of 362 Da, relating to 4-oxochatancin (**16**) or 1-oxo-9-hydroisochatancin (**18**). Peaks with retention times around 14 min in the chromatograms of VLC 5 and 6 contained a metabolite showing an *m*/*z* of 319.23 (M + H) and 341.21 (M + Na), which indicated the presence of bisepoxide **12**, having a molecular weight of 318.45 Da. A mass charge ratio of 475.39 (M + H − H_2_O) and 493.39 (M + H), found for the peak with a retention time of 14.7 min of the UPLC-chromatogram of VLC fraction 7, is characteristic for the secosteroid **1** or the polyhydroxylated steroid **4**, both with a molecular weight of 492 Da.

More importantly, UPLC–HRMS investigations produced also some peaks with *m/z* values that cannot be linked to isolated compounds **1**–**19**. Thus, in VLC fraction 7 and 8 an *m/z* value of 287.24 (M + H) indicates most probably the presence of the instable sarcophytonin A with a molecular weight of 286.23 Da [16]. VLC fractions 7 and 8 also contain *m/z* values characteristic for steroid constituents of *Sarcophyton* soft corals that could not be isolated in the current study, e.g., *m/z* 397.35 (M + H) suggests the presence of a steroid compound reported by Kobayashi et al. from *Sarcophyton glaucum* [17] with a molecular mass of 396 Da as outlined in Table S7B (Supporting Information File 1). Detailed analysis of the UPLC–HRMS data also revealed the presence of *m*/*z* 711.39 (M + H) and 669.44 (M + H) in VLC fractions 6 and 7. These values would fit to not yet reported biscembranoids, containing compound **5** as a possible biogenetic precursor, with a suggested molecular mass of 710 and 668 Da (see Supporting Information File 1, Table S7B).

Notable is the occurrence of numerous peaks containing *m*/*z* values attributable to isomers of the isolated metabolites. Besides the isosarcophines **8** and **9** with a molecular weight of 316 Da, *m*/*z* values 317.21 (M + H) were also detected in the chromatograms of the fractions VLC 6 and 7 in different chromatographic peaks (*t*_R_: 12.0, 12.7, 14.0, 15.9 min) indicating the presence of further possible cembranoid isomers as shown in Table S7A (Supporting Information File 1). The *m*/*z* values (M + H, 739.44) attributable to the isobisglaucumlides B (**14**) and C (**15**) are also found at four different retention times of the UPLC chromatograms, suggesting the presence of the further isomeric metabolites. These findings highlight the amazingly complex and diverse metabolome of *P. longicirrum*.

**Figure 2:**
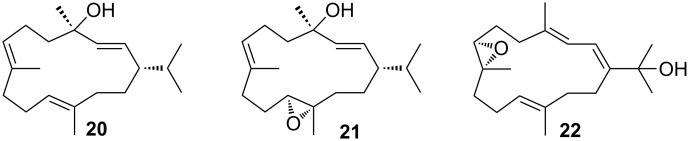
Structures of secondary metabolites from *P. longicirrum* as described by Coll et al. in 1985 [13].

Regarding the reported secondary metabolites of *P. longicirrum* **20–22**, Figure 2 by Coll et al. [13], only compound **22** with a molecular weight of 304 Da resulting in an *m/z* of 305.25 (M + H) may be present in VLC 8 (see Supporting Information File 1, Figure S7B). However, there are about 20 further cembranoids described from *Sarcophyton* spp. with a molecular weight of 304 Da, making this assessment very tentative. It can, however be stated that the *P. longicirrum* specimen investigated in this study either belongs to a different chemotype or has different food preference than the one investigated by Coll and co-workers [13].

### Detailed chemical investigation of *P. longicirrum* including structure elucidation of the new metabolites **1**, **5**, **9**, **14**, **15** and stereochemical assignment of **12**

Repeated fractionation of VLC fractions 5–8 resulted in the isolation of a range of secondary metabolites, i.e., four steroids **1–4**, nine cembranoid diterpenes **5–13** and two biscembranoids **14** and **15**, as well as four polycyclic diterpenes of the chatancin type **16–19** [12]. Compounds **1** and **5** are new chemical structures. The same applies to the biscembranoids **14** and **15**, which however show close resemblance to bisglaucumlides B and C [18], but differ in their stereochemistry from the latter. Since no studies regarding the stereochemical features of the cembranoid bisepoxide **12** were published to date, we propose here its relative configuration. Figure 1 summarizes all metabolites found during this investigation*.* It is noteworthy, that the previously reported cembranoid diterpenes (see Figure 2, compounds **20–22**) from *P. longicirrum* by Coll et al. [13] were not isolated from the complex secondary metabolome of the investigated specimen, although the UPLC–HRMS data (see above) suggest that cembranoid alcohol **22** may be present.

Compound **1** was isolated as amorphous white solid. The specific optical rotation was measured in chloroform (*c* 0.1), giving [α]_D_^20^ −21.0. The molecular formula C_30_H_52_O_5_ was established by a HRMS measurement, which yielded *m/z* 515.3694 [M + Na] for the molecular ion. The ring double bond equivalent (RDE) was calculated to be five. The IR spectrum revealed the presence of hydroxy groups (broad band at 3360 cm^−1^) and a keto function (sharp band at 1697 cm^−1^).

The planar structure of **1** was established by extensive NMR experiments (^1^H, ^13^C NMR, COSY, DEPT, HSQC and HMBC (see Supporting Information File 1, Table S1). The ^13^C NMR spectrum showed 30 resonances attributable to 7 methyl, 9 methylene and 8 methine groups. A ^13^C NMR resonance at 218.4 ppm confirmed the keto group (C-9), whereas a primary alcohol moiety was evident from a ^13^C NMR resonance at δ_C_ 59.1 (C-11). Further oxygenated carbons, i.e., C-3, C-5 and C-6 gave rise to ^13^C NMR resonances at δ_C_ 68.0, 80.7 and 75.7, respectively. Proton carbon assignments were done according to correlations obtained in a HSQC experiment. The absence of ^13^C NMR resonances for sp^2^ hybridized carbons for C=C bonds, together with a RDE of five indicated the presence of several rings in **1**, likely of steroid origin. The latter is supported by characteristically shielded ^1^H NMR resonances at δ_H_ 0.54 and δ_H_ −0.05 (both dd, H_2_-30) as well as a multiplet at δ_H_ 0.32 (H-22) for a cyclopropyl group, as typically found in gorgosterols [19–20].

**Figure 3:**
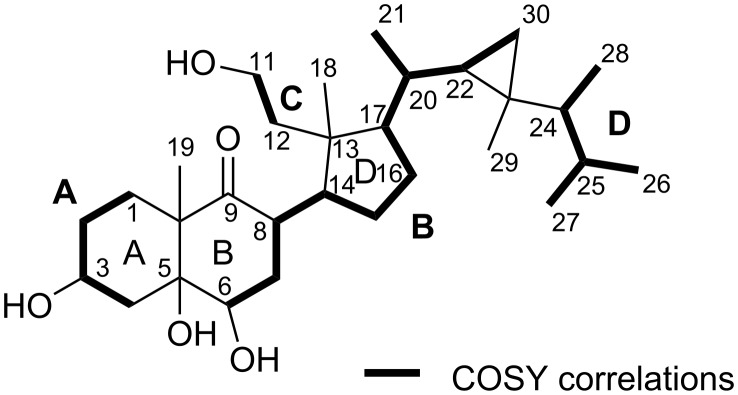
Significant ^1^H,^1^H COSY correlations as found in compound **1**.

A ^1^H,^1^H COSY experiment led to partial structures which could be combined using HMBC correlations. Spin system **A** included H_2_-1 to H_2_-4, whereas H-6 through to H-30 formed spin system **B** (see Figure 3). The connection of partial structures **A** and **B** was established from HMBC correlations, i.e., from the resonances of H-4 to C-5 and C-6, as well as H-6 to C-5. The position of the C-9 ketone function was established due to HMBC correlations from resonances of H_2_-7 and H-8 to C-9. The decaline system was finally confirmed by the heteronuclear long range correlations of the resonances from H_3_-19. Of the five degrees of unsaturation one is ascribed to a keto function, another one to the cyclopropane ring in the side chain, and two further ones to the decaline ring, thus requiring a further ring in **1**. Considering this, a secosterol backbone was likely. Also, the ^13^C NMR resonance of the oxygenated methylene at δ_C_ 59.1 (C-11) is characteristic for marine-derived secosterols [21]. The ^1^H-^1^H spin system **C** only including H_2_-11 and H_2_-12 was connected to the partial structure **B** via long range correlations from H_3_-18 to C-12, C-13, C-14 and C-17. This also established the still required ring D. Finally, the complete gorgosterol side chain could be elucidated by connection of the ^1^H,^1^H spin system **D** with **B** using HMBC correlations from H_3_-28 to C-23 and from H_3_-29 to C-22, C-23, C-24 and C-30 (see Figure 3).

**Figure 4:**
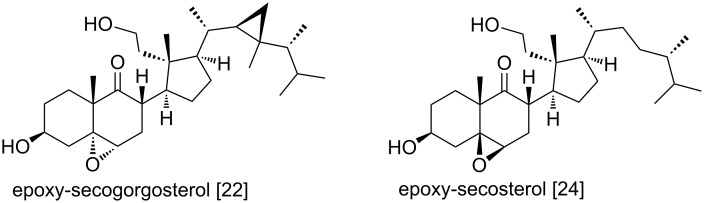
Secosterols [22,24] related to 3β,5α,6β-trihydroxy-9-oxo-9,11-secogorgostan-11-ol (**1**) from *P. longicirrum*.

The NMR data (Supporting Information File 1, Table S1) of compound **1** resembled most closely those of epoxy-secosterols isolated from the gorgonian *Pseudopterogorgia americana* [22] and from the soft coral *Pachyclavularia violacea* (now *Briareum violaceum* [23]) by Anta et al. ([24], Figure 4). However, the ^13^C NMR chemical shifts of C-5 and C-6 in compound **1** (δ_C_ 80.7 and δ_C_ 75.7, respectively) differ from shifts for the equivalent carbons in epoxy-secogorgosterol reported by Naz et al. (δ_C_ 61.0 and δ_C_ 60.4, respectively; [22]) and from those of the epoxy-secosterol reported by Anta et al. (δ_C_ 65.5 and δ_C_ 58.1, respectively; [24]). The downfield shift, observed for these carbons in **1**, results from the cleavage of the epoxide ring, and ^13^C values around δ_C_ 70–80 as observed for **1** are characteristic for hydroxylated carbons.

The relative stereochemistry of the secogorgosterol **1** was established by analysis of ^1^H,^1^H coupling constants, NOESY data, and comparison of NMR spectral data with those of similar compounds [22,24]. An equatorial orientation of the OH-group at C-3 was evident, since H-3 displayed ^1^H,^1^H coupling constants to the vicinal axial H-2β and H-4β of 12 Hz and to the equatorial H-2α and H-4α of 6 Hz. NOE correlations of H-3 to H-1α, H-2α and H-4α indicate thus an α-orientation of axial H-3 and a β-orientation of the equatorial hydroxy group at C-3. The identical ^13^C NMR shift of C-3 (δ_C_ 68.0) with the reported value [24] supports this orientation.

**Figure 5:**
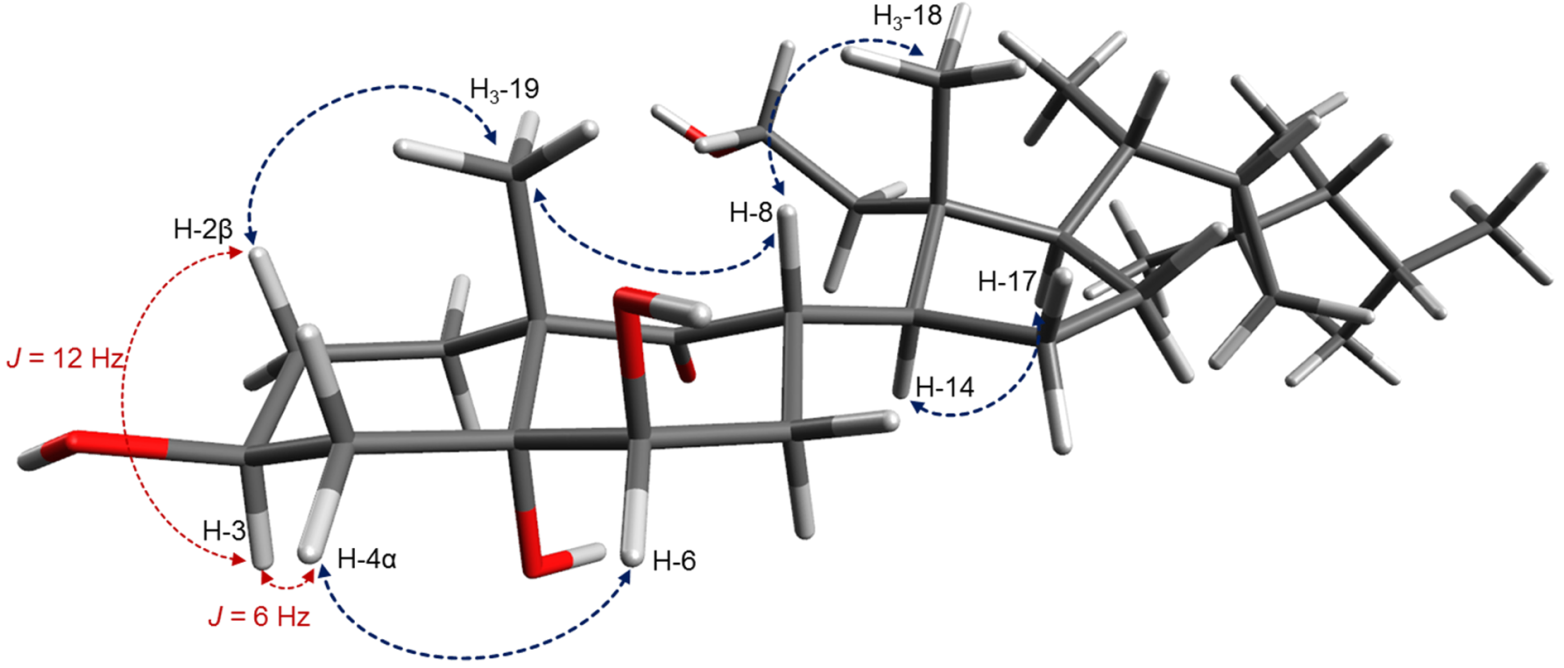
Conformational structure of **1** (key NOESY correlations are indicated with blue arrows; coupling constants crucial for the determination of the orientation of the 3-OH group are shown).

^1^H NMR measurements in pyridine-*d*_5_ led to a further downfield shift of the deshielded H-3 resonance to δ_H_ 4.81 (compared with δ_H_ 4.00 in MeOH-*d*_4_). This shift is explained by a 1,3 axial–axial interaction with the 5α hydroxy group [25–26], demonstrating the α-orientation of the substituent at the bridge head carbon C-5. NOEs between the resonances for H-2β as well as H-1β to H_3_-19 showed the latter to be β-orientated, and thus the trans configuration of the decaline system. The ^1^H NMR signal at δ_H_ 3.66 for H-6 exhibited NOE correlation with H-4α (δ_H_ 1.70, m) indicating β-orientation of the equatorial OH-group at C-6. Contrary to the reported epoxy-secosteroid by Naz et al. [22], no NOE was observed between the resonances of H-6 and β-oriented H_3_-19 confirming the β-orientation of the hydroxy group at C-6 (see Figure 5).

NOE correlations between H_3_-19 and H-8, as well as between H-8 and H_3_-18 showed the β-orientation of the methyl groups CH_3_-18, CH_3_-19 and of the proton at C-8, which is in accordance with reported stereochemistry for the related metabolites of this compound-class [21–22]. Free rotation along the bond between C-8 and C-14 is unlikely because of the bulky substituents on ring D. NOE correlations were observed between α-oriented H-14 and H-17 demonstrating β-orientation of the gorgosterol side chain. ^13^C NMR shifts for the carbons of the side chain (C-20 to C-30: δ_C_ 36.3, 21.4, 33.3, 26.9, 52.2, 33.4, 22.7, 21.9, 15.8, 14.7, 22.2) were almost identical with those reported by Naz et al. (C-20 to C-30: δ_C_ 34.9, 20.8, 31.9, 25.9, 50.5, 31.4, 22.3, 21.5, 15.2, 14.2, 21.2) [22]. The relative stereochemistry of the gorgosterol side chain was thus suggested to be the same. For the compound **1** we propose the name 3β,5α,6β-trihydroxy-9-oxo-9,11-secogorgostan-11-ol.

Chemical structures of the polyhydroxylated steroids **2**–**4** were established by comparison of the NMR and MS data obtained in our laboratory (Supporting Information File 1, Figures S6–11) with the reported values [27–28].

Compound **5** was isolated as colorless oil (1.5 mg). The specific optical rotation was measured in chloroform (*c* 0.09), and yielded [α]_D_^20^ +3.5. The molecular formula of compound **5** was deduced by HRMS–ESI (M + Na 355.2244 Da) to be C_21_H_32_O_3_. Ring double bond equivalents (RDE) were calculated to be six. The IR spectrum of compound **5** showed absorptions for carbonyl bonds at 1700 cm^−1^ and 1679 cm^−1^, indicating the presence of ketone and/or ester functions.

Extensive NMR measurements (^1^H, ^13^C NMR, COSY, DEPT, HSQC and HMBC, see Supporting Information File 1, Table S2) revealed the presence of a methoxy group (δ_H_ 3.71 3H, δ_C_ 52.1), an ester carbonyl (δ_C_ 170.0 , C-18) and a keto group (δ_C_ 211.3 , C-2). The ^13^C NMR spectrum of compound **5** contained a total of 21 resonances attributable to 5 methyl, 6 methylene, 5 methine and 5 quaternary carbons as indicated by a DEPT 135 experiment. Six characteristic shifts in the ^13^C NMR spectrum at δ_C_ 130.5 (C-4) and 142.8 (C-5), 121.6 (C-7) and 136.9 (C-8), 127.0 (C-11) and 135.4 (C-12) pointed towards three carbon–carbon double bonds. Together with two carbonyls (at C-2 and C-18) one RDE accountable to a ring remained, and suggested a cembrane-class diterpene.

**Figure 6:**
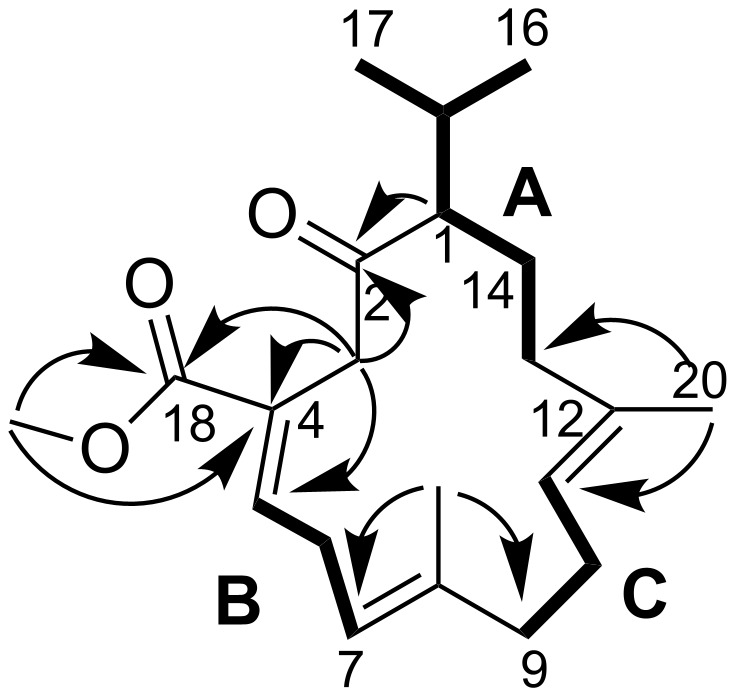
Structure of cembranoid **5**. ^1^H,^1^H spin systems (**A**, **B** and **C**) are indicated in bold, arrows show key HMBC correlations.

The proton resonances could be unambiguously assigned to those of directly attached carbons by a HSQC measurement, and afterwards the fragments of the molecule were elucidated using a COSY experiment. Thus, the COSY data showed correlations of the resonances H_3_-16, H_3_-17 and H-1 to H-15, forming an isopropyl moiety. Together with COSY correlations from H-1 over H_2_-14 to H_2_-13 spin system **A** was established . Two further smaller fragments were established via COSY correlations from H-5 to H-7 (**B**), and from H-9 to H-11 (**C**). These subunits could be assigned to a 14-membered cembrane skeleton according to couplings detected in the HMBC experiment. Key heteronuclear long range correlations for assembling the complete structure were from H_3_-20 to C-11, C-12 and C-13 connecting fragments **A** and **C**. The fragments **B** and **C** were then connected according to HMBC cross peaks of the methyl group resonance H_3_-19 with quaternary C-8 and with C-7 and C-9 (see Figure 6).

The absence of a further ^1^H,^1^H spin system required heteronuclear long range correlations for the elucidation of the remaining structural features and the closure of the cembrane ring. Methylene group CH_2_-3 exhibited HMBC correlations with resonances of the keto at C-2, sp^2^ quaternary carbon C-4 and tertiary carbon C-5 and with the ester carbonyl C-18. Due to the deshielded nature and large ^1^H-coupling constant (signals of both protons appear as doublets, *J* = 17.7 Hz at δ_H_ 3.49 and 3.64) of the ^1^H NMR resonance of the H_2_-3, the position of the methylene group between keto carbonyl C-2 and the quaternary sp^2^ carbon C-4 was very likely. The methyl ester moiety could be localized at C-4 due to long range correlation of the -OCH_3_ resonance with ester carbonyl C-18 and quaternary C-4. A HMBC cross peak between the resonances of H-1 and C-2 established the 14-membered cembranoid ring.

The *E*-geometries at olefinic double bonds Δ^7,8^ and Δ^11,12^ were easily deduced from the ^13^C NMR upfield shifts of the methyl group resonances CH_3_-19 (δ_C_ 16.0) and CH_3_-20 (δ_C_ 15.2). The deshielded resonance of H-5 (δ_H_ 7.08) indicated *E*-geometry of the olefinic double bond Δ^4,5^ [29].

**Figure 7:**
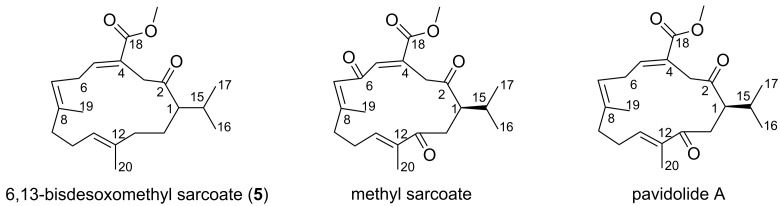
Compound **5** and the most closely related cembranoids from soft corals.

Compound **5** resembled most closely the recently reported cembranoid pavidolide A isolated from the soft coral *Sinularia pavida* [30] and a metabolite from *Sarcophyton glaucum* – methyl sarcoate reported by Ishitsuka et al. [29] (see Figure 7). Obvious differences, however were the smaller number of keto groups in compound **5** (only one at C-2 instead of two at C-2 and C-13 as in pavidolide A or three at C-2, C-6 and C-13 in methyl sarcoate). Both related molecules and compound **5** have only one stereogenic center, i.e., at C-1. The reported absolute configuration of pavidolide A ([α]_D_^20^ +124, *c* 0.25, CHCl_3_) at C-1 is *R* [30]. The specific rotation value of **5** is +3.5 and does not allow indicating any configuration for C-1 in compound **5**. Due to the instability of the substance and its rapid degradation, it was not possible to determine the absolute configuration unambiguously. Structural similarity of the compound **5** with pavidolide A and close relationship between *Sinularia* and *Sarcophyton* (food source of *P. longicirrum*) soft corals makes similar biosynthetic pathways involved into the synthesis of the cembranoid **5**, methyl sarcoate and pavidolide very likely. Thus, the configuration at C-1 in the compound **5** is proposed to be also *R*, although detailed studies are necessary to determine the stereochemistry unambiguously. Due to the close relationship to the first discovered methyl sarcoate we propose the trivial name 6,13-bisdesoxomethyl sarcoate for **5**.

Known compounds **6–8**, **10**, **11** and **13** were unambiguously identified comparing the obtained ^1^H and ^13^C NMR spectral data with the literature reports [31–36].

Repeated HPLC separation of VLC fraction 7 firstly led to two metabolites, which could not be structurally analyzed due to their instability. It was noted however, that two stable degradation products resulted and could be isolated, i.e., compounds **8** and **9**. The planar structure of **8** and **9** was established as that of isosarcophine by 1D and 2D NMR data (^1^H, ^13^C, COSY, HSQC and HMBC). Specific optical rotation measurements in chloroform (*c* 0.1 each substance) yielded [α]_D_^20^ values of +92.0 for **8** and −38.0 for **9**. Due to the close similarity of the ^1^H and ^13^C NMR data (Supporting Information File 1, Table S3) compounds **8** and **9** were supposed to be stereoisomers. NMR spectral data of compound **8** were identical with those of (+)-isosarcophine ([α]_D_^20^ +235.3) reported by Kusumi et al. [32], so **8** is established as (+)-isosarcophine. The configuration at C-2 for **8** and **9** was established with the help of CD experiments (Supporting Information File 1, Figures S22 and S27). According to Kobayashi et al. [31] (*S*) configuration at C-2 in furanocembranoids causes a negative Cotton effect at 246 nm like we obtained for compound **8**, which is thus (2*S*)-isosarcophine. The CD spectrum of **9** was the inverse of **8** and displayed a positive Cotton effect at 246 nm demonstrating that **8** and **9** are diastereomers. Thus, compound **9** is 2*R*-isosarcophine.

The cembranoid bisepoxide **12** was isolated as colorless oil, with a specific optical rotation of [α]_D_^20^ −44.6 (*c* 3.3 in chloroform). The planar structure was deduced by the interpretation of the experimental data. The obtained ^1^H and ^13^C NMR data resembled those of closely related isosarcophytoxides **10** and **11**. An obvious difference between the isosarcophytoxides and compound **12** in the ^13^C NMR spectrum was the absence of two downfield resonances resulting from the lack of a carbon–carbon double bond. Instead, two characteristic ^13^C NMR shifts at δ_C_ 66.4 and 62.3, attributable to an epoxide moiety were present. ^13^C NMR data of **12** and the specific optical rotation were identical with those reported by Bowden et al. [37], ([α]_D_^20^ –46.7, *c* 0.9 in chloroform) for the cembranoid bisepoxide isolated from the soft coral *Sarcophyton* sp., pointing towards the same structure for this secondary metabolite. However, no 2D NMR data or stereochemistry of this molecule was reported up to now. Here we include the results of 2D NMR (COSY, HMBC and NOESY; all NMR spectral data in Supporting Information File 1, Figures S32–36 and Table S4) experiments and propose the absolute configuration of bisepoxide **12**.

**Figure 8:**
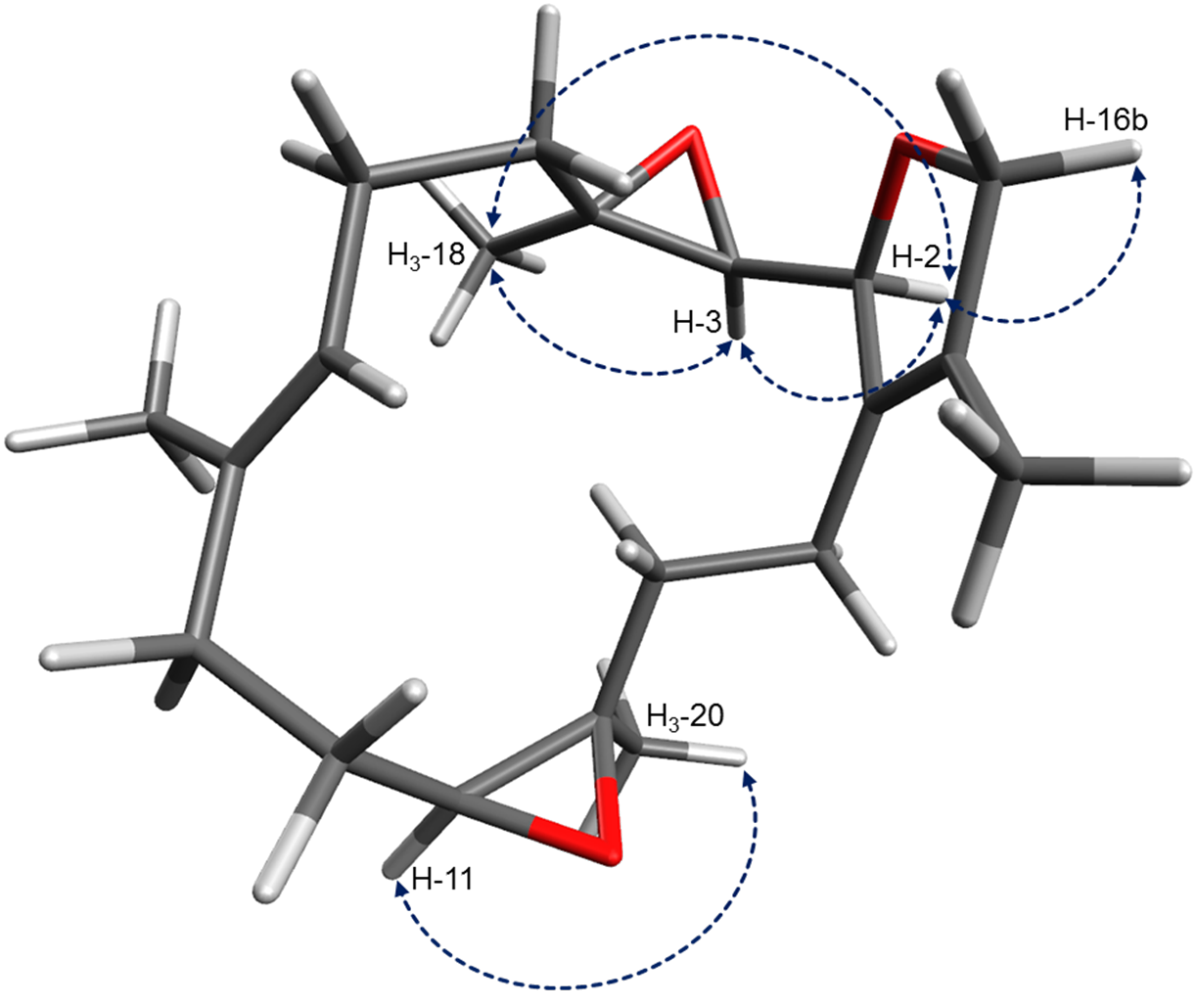
Proposed configuration and selected NOE correlations of bisepoxide **12** (key NOE correlations are indicated with blue arrows).

Bisepoxide **12** has five stereogenic centers, found at the epoxide moieties between C-3 and C-4, and C-11 and C-12, as well as C-2 of the dihydrofuran ring. Previous studies on the stereochemistry of furanocembranoids [31,36,38–39] stated a relation between the value of the specific optical rotation, the CD Cotton effect and the configuration at C-2. Thus, a large positive specific rotation value corresponds with *S* configuration at C-2, whereas negative rotation values are found for 2*R* furanocembranoids. Likewise, a negative Cotton effect at ~250 nm is observed for (2*S*)-furanocembranoids. Kobayashi et al. reported a 2*S* configuration for sarcophytonin B (**6**) which exhibited a negative Cotton effect at 247 nm and a specific optical rotation of [α]_D_^20^ +160 [31]. X-ray structure determination performed by Bernstein et al. for the closely related sarcophine (epoxide function at C-7, C-8) revealed also 2*S* configuration [38]. Sarcophine exhibited a negative Cotton effect at 246 nm. A CD measurement of bisepoxide **12** in acetonitrile did not yield any significant Cotton effect at 250 nm. However, NOE correlations are observed between the resonances of H-2, H-3 and H_3_-18 indicating the orientation of the proton H-3 and the methyl group to the same side (see Figure 8). Due to the negative optical rotation of **12** ([α]_D_^20^ −44.6), we propose the relative configuration at C-2 to be *S* (due to the additional epoxide at carbons C-3 and C-4 the CIP priority has changed) and thus, at C-3 and C-4 to be *R*. In addition, NOE correlations between H-11 and H_3_-20 indicated a cis configured epoxide at carbons C-11 and C-12. The ^1^H NMR resonance of H-11 (δ_H_ 2.88, dd, *J* = 3.3, 9.9 Hz in MeOH-*d*_4_) in the bisepoxide **12** is very similar with the resonance of H-11 measured for 2*R*-isosarcophytoxide (**11**) (δ _H_ 2.88, dd, *J* = 3.3, 9.0, Hz), indicating that **11** and **12** have the same spatial orientation at carbons C-2, C-11 and C-12 [36]. Thus, we propose the relative configuration of **12** to be 2S*, 3R*, 4R*, 11R*, 12R*. A study on a closely related cembranoid, the 3,4-epoxysarcophytoxide [40], revealed the absolute configuration at C-2, C-3 and C-4 to be *S*, *R* and *R*, respectively. According to a similar specific optical rotation measured for 3,4-epoxysarcophytoxid ([α]_D_^20^ −52.8) the absolute configuration of **12** is likely to be the same. We propose the trivial name isosarcophytobisepoxide for compound **12**.

**Figure 9:**
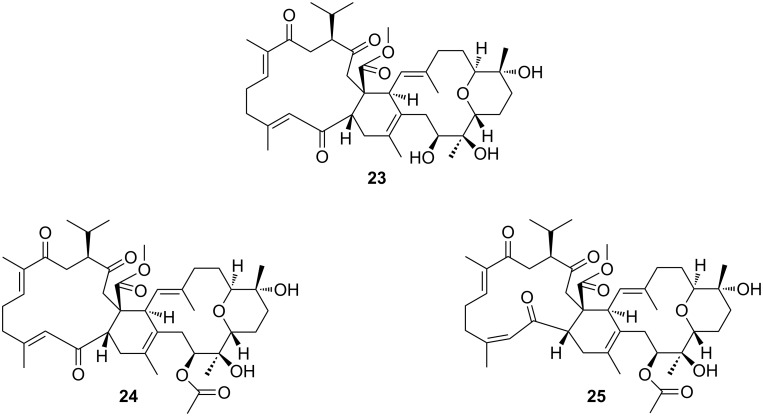
Structures of bisglaucumlids A–C (**23**–**25**).

Biscembranoid **14** was isolated as an amorphous solid, with a specific optical rotation of [α]_D_^20^ −5.0 (*c* 0.3 in MeOH). The NMR spectral data showed close resemblance with the known compounds, bisglaucumlides (**23–25**) isolated by Iwagawa et al. (see Figure 9) from the Pacific soft coral *Sarcophyton glaucum* [41]. The planar structure of compound **14** was established to be the same as that of bisglaucumlide B (**24**) by interpretation of the NMR data (^1^H NMR, ^13^C NMR, COSY, HSQC and HMBC) and comparison with the data published by Iwagawa et al. [41]. However, some ^13^C NMR resonances of the carbons in ring C and D (NMR data in Table S5, Supporting Information File 1) of **14** were significantly different from those reported for bisglaucumlide B (**24**).

In biscembranoid **14** the ^13^C NMR resonance of C-30 was shifted downfield to δ_C_ 82.1 compared with the reported value of δ_C_ 69.2 for the same carbon in bisglaucumlide B (**24**). The ^13^C NMR shift of the methyl group CH_3_-38 was more shielded (δ_C_ 16.6) than the reported resonance (δ_C_ 20.0) for **24**. The ^13^C NMR shifts of C-2 and C-21 in **14** (δ_C_ 52.4 and 43.9) also differed from **24** (δ_C_ 46.8 and 40.3, respectively). Moreover, the specific optical rotation of [α]_D_^20^ −5.0 measured for compound **14** differed significantly from the value published by Iwagawa et al. ([α]_D_ +126, *c* 0.22 in MeOH) for bisglaucumlide B (**24**) [41]. The absolute configuration of the bisglaucumlides was proposed by Iwagawa et al. according to the results of CD experiments and comparison with previously reported related molecules methyl sarcophytoate and nyalolide [29,42]. Different stereochemistry of biscembranoid **14** in the eastern part of the molecule (rings B, C and D) was thus suggested.

**Figure 10:**
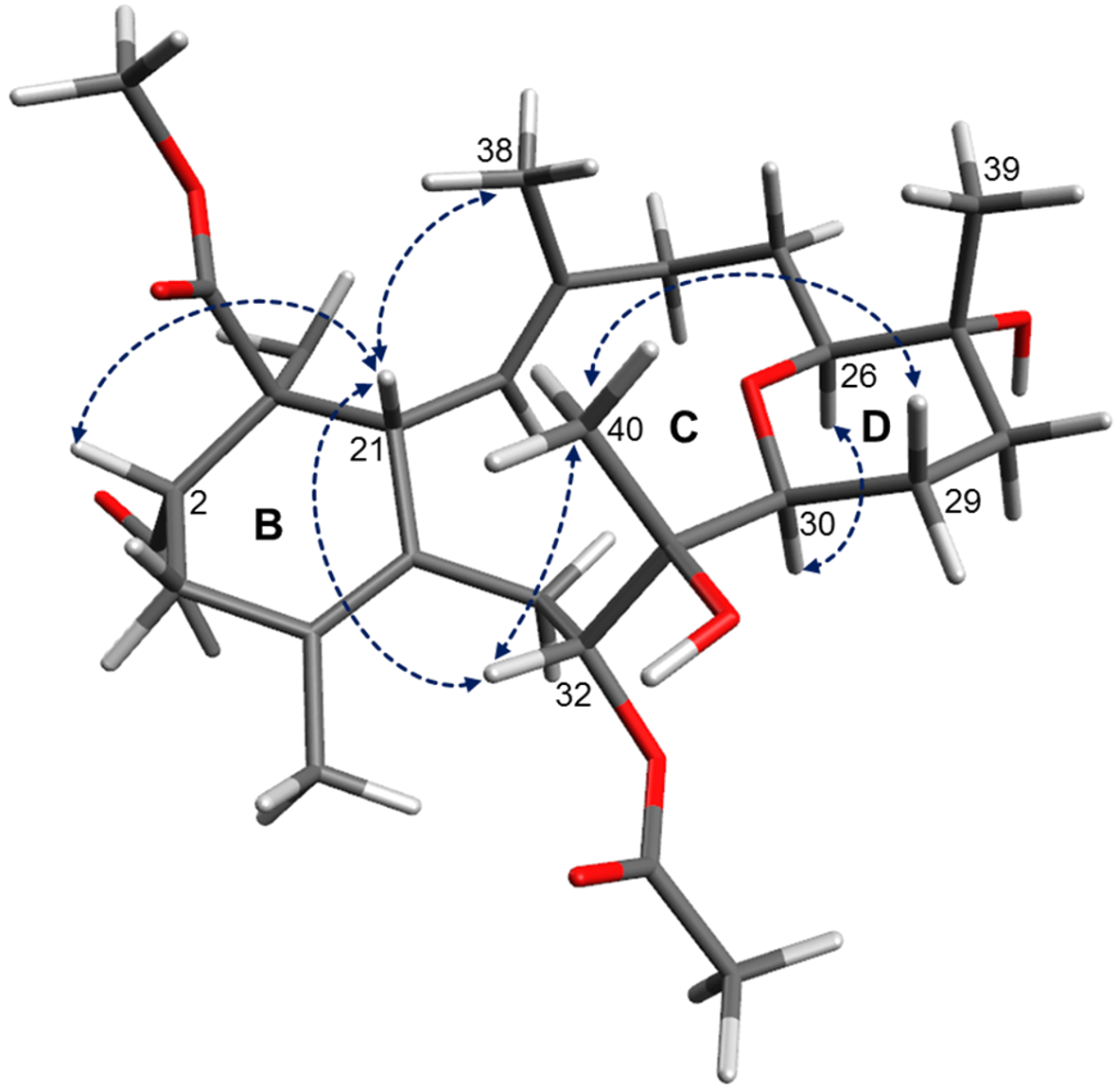
Proposed configuration of the eastern part (rings B, C and D) of isobisglaucumlides B and C (**14** and **15**; ROESY correlations are indicated with blue arrows).

A ROESY experiment showed a distinct correlation of the resonances H-26 and H-30 (see conformational fragment structure, Figure 10). The lack of an ROE correlation between H-26 and the resonance of the vicinal methyl group CH_3_-39, as well as the different ^13^C NMR shift of C-30 from the reported value for bisglaucumlide B (**24**) indicated that **14** differed in the configuration at C-30, and that both protons H-26 and H-30 are oriented to the α-face of the molecule, and the proton H-30 is axial. The methyl group CH_3_-40 is oriented to the β-face of the molecule since no ROE correlation could be observed between the latter and the α-oriented H-30. H_3_-40 has a ROESY interaction with H-29b, since the latter has a ^1^H-coupling to H-30 of 13.9 Hz it must be axial and β and this way proves the β and axial orientation of CH_3_-40. A ROE correlation between the resonance of H-32 and the H_3_-40 indicated the β-orientation of H-32, and due to the ^1^H-coupling constant of 13.9 Hz this proton must be axial. We propose the configurations in compound **14** to be *S* at C-30 and C-31, and *R* at C-32.

A further discrepancy of the biscembranoid **14** with the reported bisglaucumlide B is a ROE correlation between H-2 and H-21 indicating the β-orientation of both protons and the configuration change at C-21 from *S* to *R*.

The related biscembranoid **15** was isolated as an amorphous solid with a specific optical rotation of [α]_D_^20^ −14.0 (*c* 0.2 in MeOH). The NMR data (^1^H NMR, ^13^C NMR, COSY, HSQC, HMBC and ROESY, see Supporting Information File 1, Table S6) of **15** were very similar to those of **14**. The obvious difference was found at the ^13^C NMR resonance of the methyl group CH_3_-19. The chemical shift of δ_C_ 24.9 (compared with δ_C_ 18.6 in **14**) indicated the *Z* geometry at the carbon-carbon double bond between C-4 and C-5 in the biscembranoid **15**. This was also in accordance with the reported value by Iwagawa et al. [18] for bisglaucumlide C (**25**). The stereochemistry of the biscembranoid **15** in the eastern part of the molecule is suggested to be the same as that found in compound **14** due to the similar ROE experimental results. The difference in the specific rotation of the compound **15** [α]_D_^20^ −14.0 with the reported value for bisglaucumlide C ([α]_D_^20^ +32, in MeOH) supports the proposed stereochemistry.

We propose trivial names isobisglaucumlides B and C for compounds **14** and **15**, respectively.

### Investigation of defensive properties of isolated metabolites

**Figure 11:**
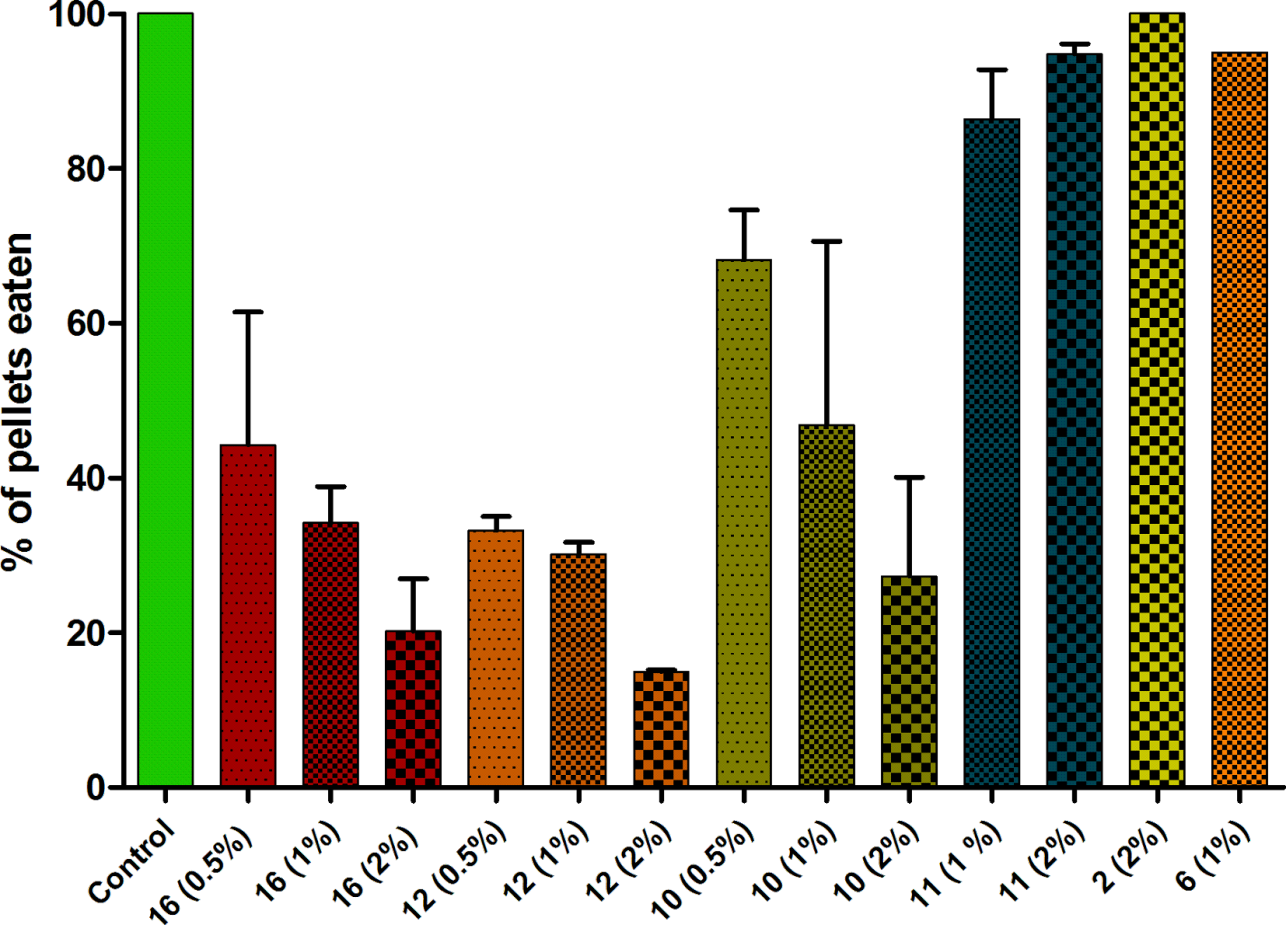
Effect of *Phyllodesmium* metabolites in different concentrations on predation by *Canthigaster solandri* (*n* = 8–40, depending on availability). Experiments were repeated twice with compounds **10–12** in all tested concentrations, twice with **16** at 1% and 2% and three times at 0.5%. Mean values with SD are displayed. Significance of deterrence was shown with Fisher´s exact test (P < 0.05 for **10**, **12** and **16**, calculated for each trial separately). Control pellets were 100% eaten for each trial.

Subsequent experiments showed the feeding deterrent activity of the major secondary metabolites in *P. longicirrum* using the omnivorous fish *Canthigaster solandri* as model predator. In preliminary assays VLC fractions 5–7 proved to have significant deterrent effects (Supporting Information File 1, Figure S57). After the isolation of pure metabolites the deterrent properties could be traced down to few metabolites. (2*S*)-isosarcophytoxide (**10**) and bisepoxide **12** were already deterrent at 0.5% of dry mass, and higher concentrations (1.0% and 2.0% of dry mass) resulted in stronger effects (see Figure 11). Surprisingly, the stereoisomer of **10**, 2*R*-isosarcophytoxide (**11**) showed no significant deterrence at concentrations up to 2.0% of dry mass. No significant activity could be attributed to the structurally related sarcophytonin B (**6**) at a concentration up to 1.0% of dry mass. Polyhydroxylated steroid **2** also showed no antifeedant activity at the concentration 2.0% of dry mass.

## Discussion

### 1. Chemical constituents of *P. longicirrum*

In this study a wide range of secondary metabolites was isolated from a single specimen of *P. longicirrum*. These belong to the polyhydroxylated steroids **1**–**4**, cembranoid diterpenoids **5**–**13** and biscembranoid tetraterpenes **14** and **15**, as well as the rare chatancin-type diterpenes **16**–**19**.

Noteworthy is the new unusual secosteroid **1** with a side chain as found in gorgosterol. Steroids with such a side chain were first described from the gorgonian coral *Plexaura flexuosa* [43]. Later on, the so-called gorgosterols were isolated from marine sediments, diatoms and most importantly from dinoflagellate cultures [44–46]. Besides inhabiting the water column, dinoflagellates of the genus *Symbiodinium* live in symbiosis as the so named zooxanthellae with various soft corals, i.e., *Sarcophyton* spp. and are also hosted by *P. longicirrum*. The secosteroid **1** isolated in this study could possibly originate either from the dinoflagellate symbionts of *Sarcophyton*, or is a product of the biosynthetic activity of the dinoflagellates within *P. longicirrum*. One possibility to explain the occurrence of chemically closely related gorgosterols in different soft coral clades is the presence of the same putative gorgosterol-producing *Symbiodinium* within their body. Overall, only few secosterols with gorgosterol side chain were reported, e.g., by Morris et al. [21], Naz et al. [22] and Anta et al. [24] and the ecological role of these compounds remains to be explored.

It cannot be ruled out completely that secosteroid **1** is an oxidation product, i.e., artifact of the secosteroid reported by Naz et al. [22] (see Figure 4). In this case, the epoxide ring at C-5 and C-6 would have undergone a cleavage to yield **1** during chromatographic procedures. The analysis of HRMS–UPLC data however, showed the presence of signals attributable to the secosteroid **1** prior the extensive HPLC (see Supporting Information File 1, Figure S53). This observation provides evidence that compound **1** is indeed a natural product, if not the epoxide moiety was cleaved already during the storage of the specimen in ethanol.

The new cembranoid 6,13-bisdesoxomethyl sarcoate (**5**) resembles most closely the methyl sarcoate described by Ishitsuka et al. [29] from soft coral *Sarcophyton glaucum* and the pavidolide A (6-desoxomethyl sarcoate) isolated from *Sinularia pavida* [30]. Methyl sarcoate is reported to be a possible biosynthetic precursor of several biscembranoids, e.g., methyl sarcophytoate [47], a metabolite from *S. glaucum* and methyl neosartortuate from *S. tortuosum* [48]. The biscembranoids are believed to originate as products from Diels–Alder cycloaddition between two cembranoid units. Besides methyl sarcoate, only methyl tetrahydrosarcoate and isosarcophytonolide D are reported to act as dienophile (western part) in over 60 described biscembranoids [49]. The western parts in the bisglaucumlides [18,41] and isobisglaucumlides isolated in this study (**14** and **15**) correspond with the structure of methyl sarcoate. To our best knowledge, there are no literature reports on biscembranoids having 6,13-bisdesoxomethyl sarcoate (**5**) or pavidolide A as building blocks. First evidence pointing towards the existence of such biscembranoids provides the UPLC–HRMS investigation in this study. As mentioned in the results part, characteristic *m*/*z* values were detected in the chromatograms of the fractions VLC 6 and 7 (Supporting Information File 1, Figures S54–56) attributable to biscembranoids incorporating compound **5** as the western part and the eastern part as found in the isobisglaucumlides **14** and **15** (M + H *m*/*z* 711.39). A further possible metabolite with *m*/*z* of 669.44 (M + H) would be one with the eastern part bearing a hydroxy instead of the acetate moiety at C-32 (as found in bisglaucumlide A (**23**) [18]). This assignment relying on the MS data only is surely somewhat speculative.

In our former study in 2014, relying on UPLC-HRMS data only, we assumed that isosarcophines **8** and **9** and sarcophytonin B (**6**) could be present in the *P. longicirrum* extract. In the current investigation we were able to isolate these metabolites and demonstrate the informative value of the preliminary UPLC–HRMS analysis. Further investigations on Alcyonacean and/or *Phyllodesmium* chemistry may thus lead to the isolation and full characterization of the putative biscembranoids.

Furanocembranoids are reported to be unstable and to quickly autoxidize in the presence of light [16]. In our study, isosarcophines **8** and **9** were isolated after repeated chromatography of purified, but labile compounds, which thus remained unidentified. Additionally, during the purification of the isosarcophytoxides **10** and **11** peaks most probably attributable to the isosarcophines **8** and **9** appeared in the chromatograms, demonstrating the oxidation of the isosarcophytoxides to the isosarcophines. Reports on such conversions are found in the literature, e.g., Kusumi et al. described the isolation of isosarcophine **8** from *Sinularia mayi* and chemical conversion of the isosarcophytoxide **10** into **8** by exposing the DCM solution of **10** to light, supporting our assumption that the isosarcophines **8** and **9** are artifacts [32].

### 2. Secondary metabolome of *P. longicirrum* with regard to metabolites known from *Sarcophyton*, especially in *S. glaucum*

Several *Sarcophyton* species (Octocorallia) were reported to contain the identical metabolites as isolated in this study from *P. longicirrum*. Indeed, the majority of the known compounds was firstly found in *S. glaucum* (**2**, **4**, **10**–**11**, **13** [27,35–36]). Other *Sarcophyton* species reported to contain *P. longicirrum* metabolites are *S*. *subviride*, i.e., compound **3** [28] and *S*. *cherbonnieri*, i.e., compound **7** [33]. However, two of our *P. longicirrum* compounds, sarcophytonin B (**6**) and isosarcophytobisepoxide (**12**) were first described from unidentified *Sarcophyton* species [31,37]. Concerning the new natural products from *P. longicirrum*, they can be regarded as derivatives of *S. glaucum* secondary metabolites, e.g., **1**, **5**, **14**, **15** and the chatancin-type diterpenes **16**–**19**.

Samples of the same *Sarcophyton* species collected at different times and habitats can differ greatly in their chemistry [39,50]. Other closely related soft corals, such as *Lobophytum* spp., also contain cembranoids, e.g., the isosarcophytoxides **10** and **11** [36] as well as a numerous biscembranoid compounds [51]. Distinct patterns of chemotypes in *Sarcophyton* species were demonstrated in a study by Tanaka et al. [52]. In this latter study, the largest diversity of secondary metabolites was found for *S. glaucum*, compared with the moderate chemical diversity found in *S. trocheliophorum*. The latter was the observed soft coral preyed upon by *P. longicirrum* and investigated by Coll et al. [13]. The isolation of only three metabolites **20**–**22** (Figure 2) in their study would correlate with the results of Tanaka et al. [52], showing the less complex metabolome of *S. trocheliophorum*. Exact identification of soft corals is, however still difficult and misidentification cannot be ruled out.

**Figure 12:**
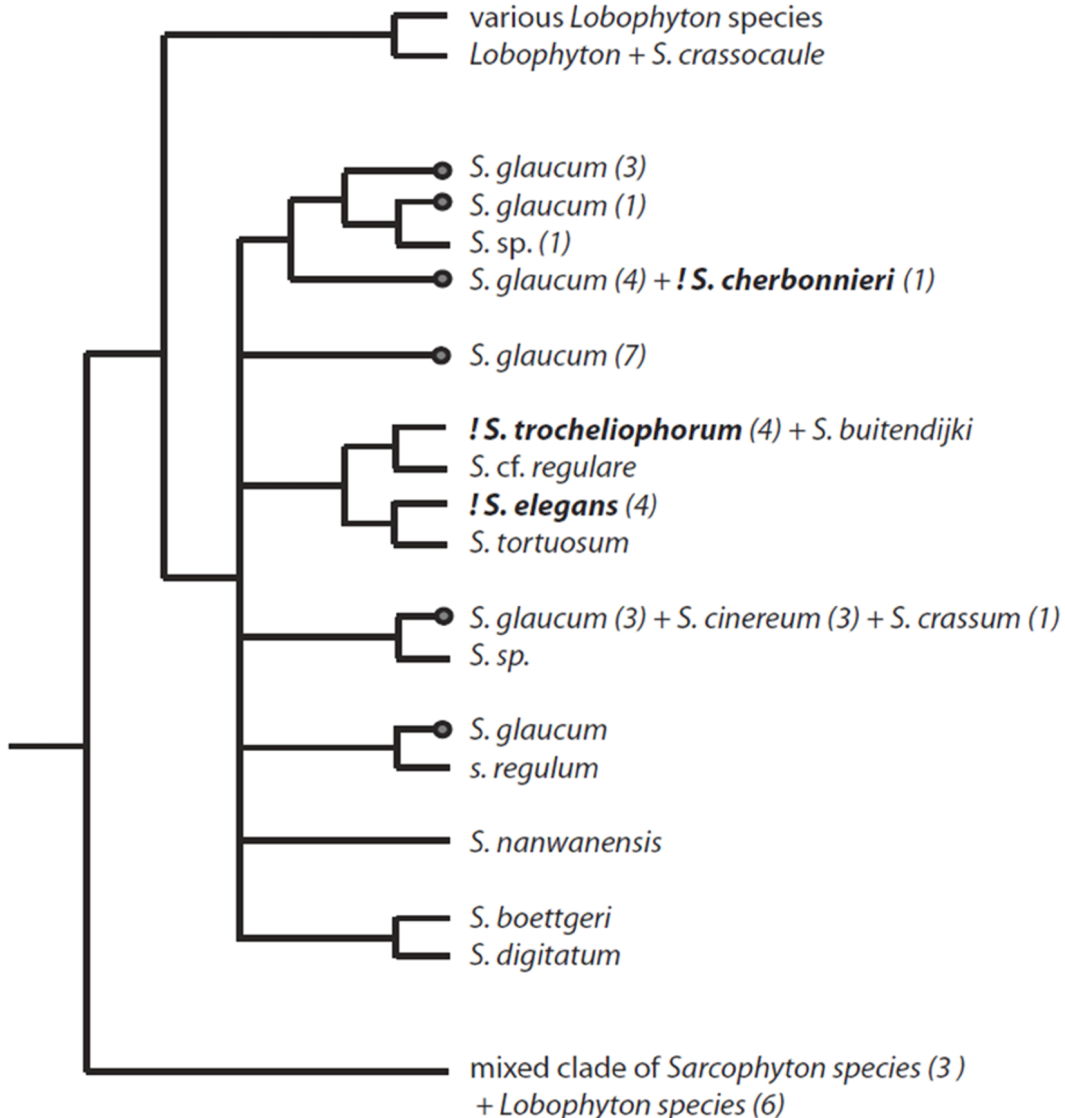
Phylogenetic tree of octocorals relevant as putative food sources for *Phyllodesmium* spp. Phylogram of *Sarcophyton* and *Lobophytum*, based on a consensus phylogram of McFadden et al. [54]. Only the *Sarcophyton* clade with species investigated with regard to secondary metabolites are given in detail. The two other clades from the original phylogram are only indicated*; Lobophytum* and the mixed clade consisting of *Sarcophyton* and *Lobophytum* species. Numbers indicate the number of specimens that represent the respective branch. Note that the single specimen of *S. cherbonnieri* groups with 4 specimens of *S. glaucum*. *S. glaucum* is not monophyletic, but is represented with several independent clades. The dots at the terminal branches of *S. glaucum* in the tree indicate that secondary metabolites are described from this species, but it is not known, from which clade. Species in bold indicate that secondary metabolites were described.

An explanation for the highest metabolome diversity of *S. glaucum* can lie in the taxonomic impediment of this particular species. The phylogenetic analysis by McFadden et al. clearly showed that the relationship and systematics of the soft coral genus *Sarcophyton* is not resolved (see Figure 12) [53]. *S. glaucum* seems to be a species complex with at least 6 different clades. Furthermore one of the former investigated species (*S. subviride*, compound **3**) is considered to be synonymous with *S. glaucum* [53]. A retrospective assignment of the investigated *S. glaucum* specimens and the identified metabolites mentioned above is not possible. Interestingly, *S. cherbonnieri* wherefrom compound **7** was first described, shows no genetic difference to four specimens identified as *S. glaucum*. The available studies on *S. cherbonnieri* secondary metabolites [33–34,52] resulted in the isolation of a range of secondary metabolites, some of which were previously described from *S. glaucum*. These results show that chemotaxonomy might help in effective species delimitation.

The occurrence of the secosteroid **1** which is a derivative of a compound isolated from the Gorgonian *Pseudopterogorgia americana* may raise further questions about the food preferences of *P. longicirrum*. The soft corals (Alcyonacea) comprise several suborders and families including Alcyoniidae within Alcyoniina, and Gorgoniidae within Holaxonia. Phylogenetic analyses based on molecular markers and applying maximum likelihood methods, as well as parsimony analyses by McFadden et al. [54] do not clarify the relationship of the morphologically, as well as chemically different Alcyoniidae (including *Sarcophyton*) and Gorgoniidae (including *Pseudopterogorgia* within Alcyonacea). Nevertheless, feeding on *Pseudopterogorgia* by *P. longicirrum* seems extraordinary and very unusual. The habitus of this gorgoniid is so different – fragile with very narrow branches – compared to the mushroom shape of *Sarcophyton*. Moreover, no typical gorgonian diterpenes (e.g., pseudopterosines, [55]) were isolated from *P. longicirrum*. As stated before, it is more likely that the secosterol is produced by zooxanthellae independently from its host and/or the *Sarcophyton* soft corals produce a wider variety of secondary metabolites as known.

Altogether, the types of the majority of the isolated metabolites provide circumstantial evidence that the investigated *P. longicirrum* specimen fed mainly, if not exclusively, on *S. glaucum* which is a common soft coral in the Indo-West Pacific coral reefs and has been observed at Lizard Island. In contrast, the animal collected from a different place in the GBR and studied by Coll et al. [13] preyed upon *S. trocheliophorum*, which is according to McFaddens and co-workers phylogenetic research [53] found in the different clade from the *S. glaucum* species complex (see Figure 12). We can state that the highly diverse metabolome of the herein studied *P. longicirrum* probably results from the different chemotypes among *S. glaucum* which might reflect the cryptic speciation as well as the wide metabolite spectrum of the *S. glaucum* complex. Whether a cryptic speciation also occurs in *P. longicirrum* by specializing on certain chemotypes within *Sarcophyton* clades remains to be clarified.

### 3. Defensive role of *P. longicirrum* metabolites

The coral reef is a harsh environment with a high predation pressure, especially for slow moving and soft bodied animals like *P. longicirrum*. In the past, studies were conducted trying to shed light on the ecology of soft corals, especially with regard to the protection against predators. A pioneering work was done by Neeman and coworkers in 1974 [56]. There, one of the first isolated cembranoid diterpenes from *S. glaucum* – sarcophine – was reported to be a defensive agent in *Sarcophyton* colonies. Sarcophine, the main toxic substance of the investigated soft coral, was shown to be ichthyotoxic in lethality assays using the freshwater fish *Gambusia affinis*, and therefore believed to be at least involved in the chemical defense. Later investigations provided toxicity ranking among the Alcyonarian soft corals. *Sarcophyton* species together with *Sinularia*, *Lemnalia*, *Lobophytum* and *Nephthea* soft corals showed the highest ichthyotoxicity levels [57–58]. The study on the defensive strategies of soft corals carried out by La Barre et al. focused on feeding deterrence of aqueous coral extracts against *G. affinis* [59]. The remarkable result was that the fish deterrent activity and ichthyotoxicity not necessarily correlated, demonstrating the complexity of such ecological studies. The comprehensive article by Pawlik provides deeper insights into the problematics concerning the determination of the defensive role of marine natural products [60].

Defense strategy using sequestered soft coral metabolites has been discussed for the members of the genus *Phyllodesmium*, however, there are only few studies supporting this hypothesis. Besides our earlier study [12] reporting a feeding deterrent activity of 4-oxochatancin (**16**), only a study by Slattery et al. [15] could show an antifeedant effect of a secondary metabolite sequestered by *Phyllodesmium*. In the latter study, acetoxypukalide, which was sequestered by *P. guamensis* from its prey corals *Sinularia* spp., successfully deterred the omnivorous pufferfish *Canthigaster solandri* under laboratory conditions at 0.5% of dry mass in artificial food. The concentration chosen was at least an order of magnitude lower, than found in the body tissues of *P. guamensis*.

Determination of the exact percentage of secondary metabolites in the body tissues of *P. longicirrum* in our study was experimentally not possible, since the single specimen investigated herein was stored in ethanol (90%) immediately after collection. Taking into account the comparable physiology and the close relationship of *P. guamensis* and *P. longicirrum*, we have chosen a concentration of 0.5% of the respective substance in the dry weight of the artificial food as lowest level (according to the study by Slattery et al. [15]) for pure compounds in laboratory feeding assays using *C. solandri*.

The major metabolites (2*S*)-isosarcophytoxide (**10**) and bisepoxide **12** were significant deterrent at 0.5% of the fish diet calculated for the dry mass. The deterrence was more pronounced at higher concentrations (1 and 2%) (see Figure 11). 4-oxochatancin (**16**), as reported earlier showed comparable activity [12]. Remarkable, however, is the inactivity of the (2*R*)-isosarcophytoxide (**11**) up to 2% of dry pellet mass, a mere stereoisomer of the active metabolite **10**. Such a dependence on stereochemistry may result from a specific interaction with taste receptors in *C. solandri* lips, oral cavity and pharyngeal part. The importance of stereochemistry for taste response was shown in studies using alanine as a ligand for taste receptors from fish [61–62]. Investigations on the effects of cembranoid diterpenes on receptors are published, e.g., nicotinic receptors by Ferchmin et al. [63].

An important chemical feature of deterrent cembranoid metabolites is the epoxy-moiety. Among the tested cembranoids the strongest deterrent effect was found for the isosarcophytobisepoxide **12**, following by (2*S*)-isosarcophytoxide (**10**). The assayed furanocembranoid sarcophytonin B (**6**) could not exhibit reproducible effect, even though one probe was highly deterrent. Probable spontaneous oxidation of **6** to potentially active epoxide-bearing metabolites, e.g*.*, (2*S*)-isosarcophytoxide (**10**) or isosarcophines **8** and **9** may have led to this observation. Deterrent abilities of the latter, however, are still not determined due to insufficiently available amounts.

Isolated steroid compounds **1**–**4** in this study are minor metabolites compared to the diterpenes present in the investigated specimen (e.g., **10**–**12** and **16**). The ecological role (defensive or ichthyotoxic) of polyhydroxylated steroids could not yet been assigned in the marine environment [59]. Here, the only assayed steroidal compound was **2**, but found to be inactive at 2% of dry mass. The ecological significance or role of this compound class in *P. longicirrum* remains unexplored.

Fish may represent the main, however not the only potential predation threat to slugs. The omnivorous Echinodermata, Crustacea and Cephalopoda could also consider *Phyllodesmium* as possible prey. Whether the compounds, proven defensive against fish predation in this study function as deterrents towards other organisms, has yet to be shown. Even if the furanocembranoids represent the protection against omnivorous fish, it is possible that some of the numerous secondary metabolites found in this study could be useful against a wider range of predators.

In summary, chemical investigation of a single large *P. longicirrum* specimen resulted in isolation of 19 secondary metabolites of terpenoid origin. Taking the metabolites detected by UPLC–HRMS analysis also into account, *P. longicirrum* demonstrates an unprecedented level of secondary metabolite diversity. The herein studied *P. longicirrum* sequesters its secondary metabolites most probably from the chemistry-rich *S. glaucum* species complex, in contrary to a previously reported investigated *P. longicirrum*. The defensive role of the major diterpenoid constituents (**10**, **12** and **16**) as feeding deterrent agents against tropical omnivorous fish *C. solandri* was shown in laboratory assays, providing further strong evidence for the use of chemical protection strategy within the scarcely investigated aeolidoidean genus *Phyllodesmium*.

## Experimental

### General experimental procedures

Optical rotations were measured with a Jasco DIP 140 polarimeter. ECD spectra were taken on a Jasco J-810 CD spectropolarimeter. UV and IR spectra were obtained using Perkin-Elmer Lambda 40 and Perkin-Elmer Spectrum BX instruments, respectively. All NMR spectra were recorded in MeOH-*d*_4_ using Bruker Avance 300 DPX, Bruker Avance 500 DRX or Bruker Ascend 600 (with cryoprobe Prodigy) spectrometers, respectively. Spectra were referenced to residual solvent signals with resonances at δ_H/C_ 3.35/49.0. LC–ESIMS was performed using an Agilent 1100 system with an API 2000 Triple Quadrupole LC/MS/MS with ESI source (Applied Biosystems/MDS Sciex) and a photodiode array detector (PDA). HRMS–ESI were recorded on a LTQ Orbitrap mass spectrometer. UPLC–HRMS analysis was performed on a Thermo Scientific Qexactive mass spectrometer with HESI source, Phenomenex Kinetex C_18_ column (150 mm × 4.6 mm, 2.6 µm, 100 Å).

A Grace Reveleris X2 system equipped with 12 g Reveleris C_18_ column was used for flash chromatography. HPLC was performed either on a Merck Hitachi HPLC system equipped with a L-6200A pump, a L-4500A PDA detector, a D-6000A interface with D-7000A HSM software, a Rheodyne 7725i injection system or on a Waters HPLC system equipped with a 1525µ dual pump, a 2996 PDA detector, Breeze software and a Rheodyne 7725i injection system. A Waters Atlantis T3 C_18_ column (250 mm × 4.6 mm; 5 µm), Macherey-Nagel Nucleoshell C_18_ column (250 mm × 4.6 mm; 5 µm), Knauer Eurospher C_18_ column (250 mm × 8 mm; 5 µm) and Phenomenex Luna 5 µm C_18_(2) 100A column (250 mm × 10 mm; 5µm) were used.

### Sample

The sample of *P. longicirrum* was collected 2008 during a field trip to Lizard Island (Great Barrier Reef, Australia) by H. Wägele. The specimen (Phlo08-LI) was stored in ethanol (96%) until further extraction and processing in the laboratories at the University of Bonn.

### Extraction and isolation

The extraction procedure was analogous as described in [11]. The ethanolic storage solution was combined with the MeOH extract (3 × 150 mL) of the slug biomass and the solvents were evaporated. After liquid–liquid separation of the methanolic crude extract (4.5 g) between 100 mL H_2_O and 3 × 100 mL ethyl acetate (EtOAc), EtOAc solubles (2.2 g) were fractionated by vacuum liquid chromatography (VLC) over Polygoprep 60–50 C_18_ stationary phase (Macherey-Nagel) using gradient elution from 20:80 (MeOH/H_2_O) to 100% MeOH to yield 11 fractions. 100 mL of the mobile phase was used for each fraction. Fractions 3–8 (6 mg, 16 mg, 207 mg, 763 mg, 696 mg, 150 mg, respectively) were analyzed with the UPLC–HRMS system using the following solvent gradient program: A. water + 0.1% formic acid and B. acetonitrile + 0.1% formic acid; 5% B 0–2 min, 5–95% B 2–14 min, 95% B 14–17 min, 95–5% B 17–22 min. The column oven was adjusted to 30 °C. Complex chromatograms obtained from VLC fractions 5–8 by UPLC–HRMS analysis indicated the presence of a wide range of secondary metabolites. These VLC fractions were thus subsequently submitted for further chromatographic separation.

VLC fraction 7 (690 mg) was further separated by normal phase VLC using silica gel (60Å, 70–230 mesh, 63–200 µm) and dichloromethane (DCM):acetone gradient from 100% DCM to 100% acetone (100 mL of eluent each fraction, 20% steps) to yield 6 fractions (7.1–7.6). HPLC of fractions 7.5 and 7.6 (MeOH/H_2_O 83:17, 0.9 mL/min, column: Waters Atlantis T3) resulted in the isolation of the new secosterol **1** (1.4 mg) along with the known steroid compounds **2** (2.5 mg), **3** (1.0 mg) and **4** (1.5 mg).

The new cembranoid diterpene **5** was isolated after RP–HPLC separation of VLC fraction 8 (MeOH/H_2_O 90:10, Phenomenex Luna column, 2 mL/min). Seven fractions (8.1–8.7) were obtained, fraction 8.2 (17.4 mg) was further purified using Atlantis T3 column (MeOH/H_2_O 78:22, 0.9 mL/min ) yielding 1.5 mg of **5**.

Sarcophytonin B (**6**) (14.0 mg), furanocembranoid **7** (2.0 mg), isosarcophines **8** and **9** (each 2.0 mg), isosarcophytoxides **10** (13.0 mg) and **11** (12.0 mg) were isolated from fraction VLC 7.2 (530 mg) after repeated normal phase VLC with a silica gel having a smaller particle size (60 Å, particle size < 63 µm, mesh < 230; DCM/acetone gradient from 100% DCM to 100% acetone in 10% steps, 50 mL of eluent each fraction) and subsequent RP–HPLC separation (MeOH/H_2_O 75:25, 0.9 mL/min, Macherey-Nagel Nucleoshell column).

VLC fraction 6 was separated on a Sephadex LH-20 column with MeOH as eluent to yield 11 fractions S1–11, using 100 mL MeOH per fraction. The major fraction S7 (412 mg) was separated on RP flash chromatography (MeOH:H_2_O 60:40, 30 mL/min; Reveleris C_18_ column, 12 g) to yield 28 mg of cembranoid bisepoxide **12**. Cembranoid **13** (1.0 mg) was obtained after the separation of fraction S8 (24 mg) on RP–HPLC (Waters Atlantis T3 C_18_ column; 70:30 MeOH/H_2_O mobile phase, 0.8 mL/min). Separation of fraction S5 (60 mg) using RP–HPLC (MeOH/H_2_O 70:30, Macherey-Nagel Nucleoshell column, 0.9 mL/min) led to the isolation of two biscembranoids **14** (4.0 mg) and **15** (2.0 mg).

**(3β,5α,6β)-Trihydroxy-9-oxo-9,11-secogorgostan-11-ol** (**1**): amorphous solid; [α]_D_^20^ −21.0 (*c* 0.1, CHCl_3_); UV (MeOH) λ_max_ (ε): 245 (190) nm; IR (ATR) ν_max_: 3360, 2958, 2925, 2871, 1697, 1468, 1370, 1165, 1029, 974, 669 cm^−1^; ^1^H and ^13^C NMR (Supporting Information File 1, Table S1); HRMS–ESI (*m*/*z*): [M + Na]^+^ calcd for C_30_H_52_O_5_Na, 515.3712; found, 515.3694.

**6,13-Bisdesoxomethyl sarcoate** (**5**): colorless oil; [α]_D_^20^ +3.5 (*c* 0.09, CHCl_3_); UV (MeOH) λ_max_ (ε): 204 (510) nm; IR (ATR) ν_max_: 3360, 2853, 1700, 1679, 1459, 1377, 1205, 1137, 633 cm^−1^; ^1^H and ^13^C NMR (Supporting Information File 1, Table S2); HRMS–ESI (*m*/*z*): [M + Na]^+^ calcd for C_21_H_32_O_3_Na, 355.2249; found, 355.2244.

**(2*R*,11*R*,12*R*)-Isosarcophine** (**9**): colorless oil, [α]_D_^20^ −38.0 (*c* 0.1 in CHCl_3_); UV (MeOH) λ_max_ (ε): 204 (11587), 260 (sh) (1587) nm; IR (ATR) ν_max_: 3445, 2924, 1748, 1677, 1440, 1385, 1093, 997 cm^−1^; ^1^H and ^13^C NMR (Supporting Information File 1, Table S3); ESIMS (*m*/*z*): [M + H]^+^ 317.1.

**(2*S*,3*R*,4*R*,11*R*,12*R*)-Isosarcophytobisepoxide** (**12**): colorless oil, [α]_D_^20^ −44.6 (*c* 3.3 in CHCl_3_); UV (MeOH) λ_max_ (ε): 206 (5716) nm; IR (ATR) ν_max_: 3431, 2925, 2854, 1711, 1457, 1378, 1267, 1247, 1205, 1142, 995 cm^−1^; ^1^H and ^13^C NMR (Supporting Information File 1, Table S4); HRMS–ESI (*m*/*z*): [M + H]^+^ calcd for C_20_H_31_O_3_, 319.2268; found, 319.2222.

**Isobisglaucumlide B** (**14**): amorphous solid, [α]_D_^20^ −5.0 (*c* 0.3 in MeOH); UV (MeOH) λ_max_ (ε): 230 (1148) nm; IR (ATR) ν_max_: 3420, 2927, 2361, 1706, 1669, 1436, 1373, 1205, 1087, 761 cm^−1^; ^1^H and ^13^C NMR (Supporting Information File 1, Table S5); HRMS–ESI (*m*/*z*): [M + Na]^+^ calcd for C_43_H_62_O_10_Na, 761.4235; found, 761.4194.

**Isobisglaucumlide C** (**15**): amorphous solid, [α]_D_^20^ −14.0 (*c* 0.2 in MeOH); UV (MeOH) λ_max_ (ε): 231 (1407) nm; IR (ATR) ν_max_: 3424, 2928, 2359, 1707, 1614, 1435, 1372, 1204, 1085, 1027, 773 cm^−1^; ^1^H and ^13^C NMR (Supporting Information File 1, Table S6); HRMS–ESI (*m*/*z*): [M + Na]^+^ calcd for C_43_H_62_O_10_Na, 761.4235; found, 761.4177.

Spectral data of literature reported metabolites **2–4**, **6–8** and **13** are found in Supporting Information File 1.

Isolation and structure elucidation of four new unusual chatancin-type diterpenes **16**–**19** from the same *P. longicirrum* specimen was reported in our recent publication [13].

### Chemical defense

To evaluate defensive properties of *P. longicirrum* secondary metabolites, feeding assays with the pufferfish *Canthigaster solandri* were carried out under laboratory conditions. This fish is a generalist feeder in the tropical Pacific living sympatric with the main food of *P. longicirrum* and thus being a putative predator of *P. longicirrum*. It was also used previously as a model predator by Rohde et al. [64], Rohde and Schupp [65]. The *C. solandri* were kept in separate 70 L flow-through tanks and fed regularly days before feeding assay in order not to change the feeding preference patterns. The artificial diet was made of 0.3 g sodium alginate and 0.5 g squid powder (if available) or commercial dry fish food filled up with purified water up to 10 g. The mixture was stirred vigorously and shortly heated up in the microwave oven. The tested fractions or pure compounds were pre-solved in a drop of ethanol in an Eppendorf tube and homogenized subsequently with 1 mL of artificial diet. After pouring the semi-liquid diet into 0.25 M calcium chloride solution using a syringe, the solidified strip was washed with sea water and cut into suitable pellets. For control only a drop of ethanol was mixed with the artificial diet. Since any color differences between control and treated pellets were detectable, no additional coloring was necessary. A control and treated pellet were offered sequentially to each *C. solandri*. If the treated pellet was rejected or spit out at least three times, a second control pellet was offered to confirm that fish had not ceased feeding. A rejection was only scored when both controls were eaten. *Fisher´s exact test* was used to show the significance of the reduced palatability of treated pellets compared to control pellets. Preliminary experiments with fractions VLC 5–7 were performed with concentration levels below estimated natural occurrence in living *P. longicirrum* (VLC 5 at 2.5%, VLC 6 at 5%, VLC 7 at 1.2% of dry artificial food mass).

After obtaining and characterizing the pure metabolites from the VLC fractions tested previously, the feeding assays were repeated. Isolated secondary metabolites were chosen for the assays depending on the amounts and compound class. Major metabolites were tested twice or three times at each different concentration to enhance the statistical power. The assays were performed with compounds **2** (at 2% of dry mass), **6** (three times at 0.5%, twice at 1% of dry mass), **10** (2× at 0.5%, 1% and 2% of dry mass), **11** (2× at 1% and 2% of dry mass), **12** (twice at 0.5%, 1% and 2% of dry mass) and as previously published with **16** (3× at 0.5%, 2× at 1% and 2% of dry mass). The number of available fish individuals involved in the assays varied from 8 to 50.

## Supporting Information

File 1 Spectroscopic data and other relevant information.
