# Supplementary material for: Secondary metabolome and its defensive role in the aeolidoidean Phyllodesmium longicirrum, (Gastropoda, Heterobranchia, Nudibranchia)
Source: Beilstein J Org Chem. 2017 Mar 13;13:502–19. doi: 10.3762/bjoc.13.50 (PMC5372768; doi:10.3762/bjoc.13.50)
Supplement: File 1 — Spectroscopic data and other relevant information. [file Beilstein_J_Org_Chem-13-502-s001.pdf]

Supporting Information
for
Secondary metabolome and its defensive role in the aeolidoidean *Phyllodesmium longicirrum*, (Gastropoda, Heterobranchia, Nudibranchia)

Alexander Bogdanov[1], Cora Hertzer[1], Stefan Kehraus[1], Samuel Nietzer[2], Sven Rohde[2], Peter J. Schupp[2], Heike Wägele[3], Gabriele M. König*[,1]

Address: [1]Institute for Pharmaceutical Biology, University of Bonn, Nussallee 6, 53115 Bonn, Germany, [2]Institute for Chemistry and Biology of the Marine Environment (ICBM), Carl-von-Ossietzki University Oldenburg, Schleusenstraße 1, 26382 Wilhelmshaven, Germany and [3]Zoologisches Forschungsmuseum Alexander Koenig, Adenauerallee 160, 53113 Bonn, Germany.

Email: Gabriele M. König - g.koenig@uni-bonn.de
*Corresponding author

Spectroscopic data and other relevant information

TABLE OF CONTENTS

Figure S1. ^{1}H NMR spectrum of 3β,5α,6β-trihydroxy-9-oxo-9,11-secogorgostan-11-ol (**1**) in MeOH-d_4 3
Figure S2. ^{13}C NMR spectrum of 3β,5α,6β-trihydroxy-9-oxo-9,11-secogorgostan-11-ol (**1**) in MeOH-d_4 3
Figure S3. ^{1}H NMR spectrum of 3β,5α,6β-trihydroxy-9-oxo-9,11-secogorgostan-11-ol (**1**) in pyridine-d_5 4
Table S1. NMR data of 3β,5α,6β-trihydroxy-9-oxo-9,11-secogorgostan-11-ol (**1**) in MeOH-d_4 5
Figure S4. UV spectrum of 3β,5α,6β-trihydroxy-9-oxo-9,11-secogorgostan-11-ol (**1**) in MeOH 6
Figure S5. IR spectrum of 3β,5α,6β-trihydroxy-9-oxo-9,11-secogorgostan-11-ol (**1**) 6
Figure S6. ^{1}H NMR spectrum of steroid **2** in MeOH-d_4 7
Figure S7. ^{13}C NMR spectrum of steroid **2** in MeOH-d_4 7
Figure S8. ^{1}H NMR spectrum of steroid **3** in MeOH-d_4 8
Figure S9. ^{13}C NMR spectrum of steroid **3** in MeOH-d_4 8
Figure S10. ^{1}H NMR spectrum of steroid **4** in MeOH-d_4 9
Figure S11. ^{13}C NMR spectrum of steroid **4** in MeOH-d_4 9
Figure S12. ^{1}H NMR spectrum of 6,13-bisdesoxomethyl sarcoate (**5**) in MeOH-d_4 10
Figure S13. ^{13}C NMR spectrum of 6,13-bisdesoxomethyl sarcoate (**5**) in MeOH-d_4 10
Table S2. NMR data of 6,13-bisdesoxomethyl sarcoate (**5**) in MeOH-d_4 11
Figure S14. UV spectrum of 6,13-bisdesoxo-methyl sarcoate (**5**) in MeOH 12
Figure S15. IR spectrum of 6,13-bisdesoxo-methyl sarcoate (**5**) 12
Figure S16. ^{1}H NMR spectrum of sarcophytonin B (**6**) in MeOH-d_4 13
Figure S17. ^{13}C NMR spectrum of sarcophytonin B (**6**) in MeOH-d_4 13
Figure S18. ^{1}H NMR spectrum of compound **7** in MeOH-d_4 14
Figure S19. ^{13}C NMR spectrum of compound **7** in MeOH-d_4 14
Figure S20. ^{1}H NMR spectrum of (2*S*)-isosarcophine (**8**) in MeOH-d_4 15
Figure S21. ^{13}C NMR spectrum of (2*S*)-isosarcophine (**8**) in MeOH-d_4 15

Figure S22. ECD spectrum of (2*S*)-isosarcophine (**8**) in MeOH 16
Figure S23. ^{1}H NMR spectrum of (2*R*)-isosarcophine (**9**) in MeOH-d_4 17
Figure S24. ^{13}C NMR spectrum of (2*R*)-isosarcophine (**9**) in MeOH-d_4 17
Figure S25. UV spectrum of (2*R*)-isosarcophine (**9**) 18
Figure S26. IR spectrum of (2*R*)-isosarcophine (**9**) 18
Figure S27. ECD spectrum of (2*R*)-isosarcophine (**9**) in MeOH 19
Table S3. ^{1}H and ^{13}C NMR data of 2*S*-isosarcophine (**8**) and 2*R*-isosarcophine (**9**) in MeOH-d_4 20
Figure S28. ^{1}H NMR spectrum of (2*S*)-isosarcophytoxide (**10**) in MeOH-d_4 21
Figure S29. ^{13}C NMR spectrum of (2*S*)-isosarcophytoxide (**10**) in MeOH-d_4 21
Figure S30. ^{1}H NMR spectrum of (2*R*)-isosarcophytoxide (**11**) in MeOH-d_4 22
Figure S31. ^{13}C NMR spectrum of (2*R*)-isosarcophytoxide (**11**) in MeOH-d_4 22
Figure S32. ^{1}H NMR spectrum of 2*S*,3*R*,4*R*,11*R*,12*R*-isosarcophytobisepoxide (**12**) in MeOH-d_4.... 23
Figure S33. ^{13}C NMR spectrum of 2*S*,3*R*,4*R*,11*R*,12*R*-isosarcophytobisepoxide (**12**) in MeOH-d_4... 23
Figure S34. IR spectrum of 2*S*,3*R*,4*R*,11*R*,12*R*-isosarcophytobisepoxide (**12**) 24
Figure S35. UV spectrum of 2*S*,3*R*,4*R*,11*R*,12*R*-isosarcophytobisepoxide (**12**) in MeOH 24
Figure S36. ECD spectrum of 2*S*,3*R*,4*R*,11*R*,12*R*-isosarcophytobisepoxide (**12**) in acetonitrile (c 0.01 mg/ml) 25
Table S4. NMR Data of 2*S*,3*R*,4*R*,11*R*,12*R*-isosarcophytobisepoxide (**12**) in MeOH-d_4 26
Figure S37. ^{1}H NMR spectrum of cembranoid (**13**) in MeOH-d_4 27
Figure S38. ^{13}C NMR spectrum of cembranoid (**13**) in MeOH-d_4 27
Figure S39. ^{1}H NMR spectrum of isobisglaucumlide B (**14**) in MeOH-d_4 28
Figure S40. ^{13}C NMR spectrum of isobisglaucumlide B (**14**) in MeOH-d_4 28
Table S5. NMR data of isobisglaucumlide B (**14**) in MeOH-d_4 29
Figure S41. UV spectrum of isobisglaucumlide B (**14**) in MeOH 30
Figure S42. IR spectrum of isobisglaucumlide B (**14**) 30
Figure S43. ^{1}H NMR spectrum of isobisglaucumlide C (**15**) in MeOH-d_4 31
Figure S44. ^{13}C NMR spectrum of isobisglaucumlide C (**15**) in MeOH-d_4 31
Table S6. NMR data of isobisglaucumlide C (**15**) in MeOH-d_4. 32
Figure S45. UV spectrum of isobisglaucumlide C (**15**) in MeOH 33
Figure S46. IR spectrum of isobisglaucumlide C (**15**) 33
Table S7A. Secondary metabolites of *P. longicirrum* isolated and identified in the current investigation in correlation with UPLC-HRMS data 34
Table S7B. Secondary metabolites reported from various *Sarcophyton* spp 35
Figure S47. UPLC-HRMS chromatogram of VLC3 37
Figure S48. UPLC-HRMS chromatogram of VLC4 38
Figure S49. UPLC-HRMS chromatogram of VLC5 39
Figure S50. UPLC-HRMS chromatogram of VLC6 40
Figure S51. UPLC-HRMS chromatogram of VLC7 41
Figure S52. UPLC-HRMS chromatogram of VLC8 42
Figure S53. Abundance of 3β,5α,6β-trihydroxy-9-oxo-9,11-secogorgostan-11-ol (**1**) (*m*/*z* 493.39 M+H) in VLC 7 43
Figure S54. Abundance of *m/z* value of 711.39 in the fraction VLC 6 44
Figure S55. Abundance of *m/z* value of 711.39 in the fraction VLC 7 45
Figure S56. Abundance of *m/z* value of 669.44 in the fraction VLC 7 46
Figure S57. Preliminary results of feeding deterrence assay. 47

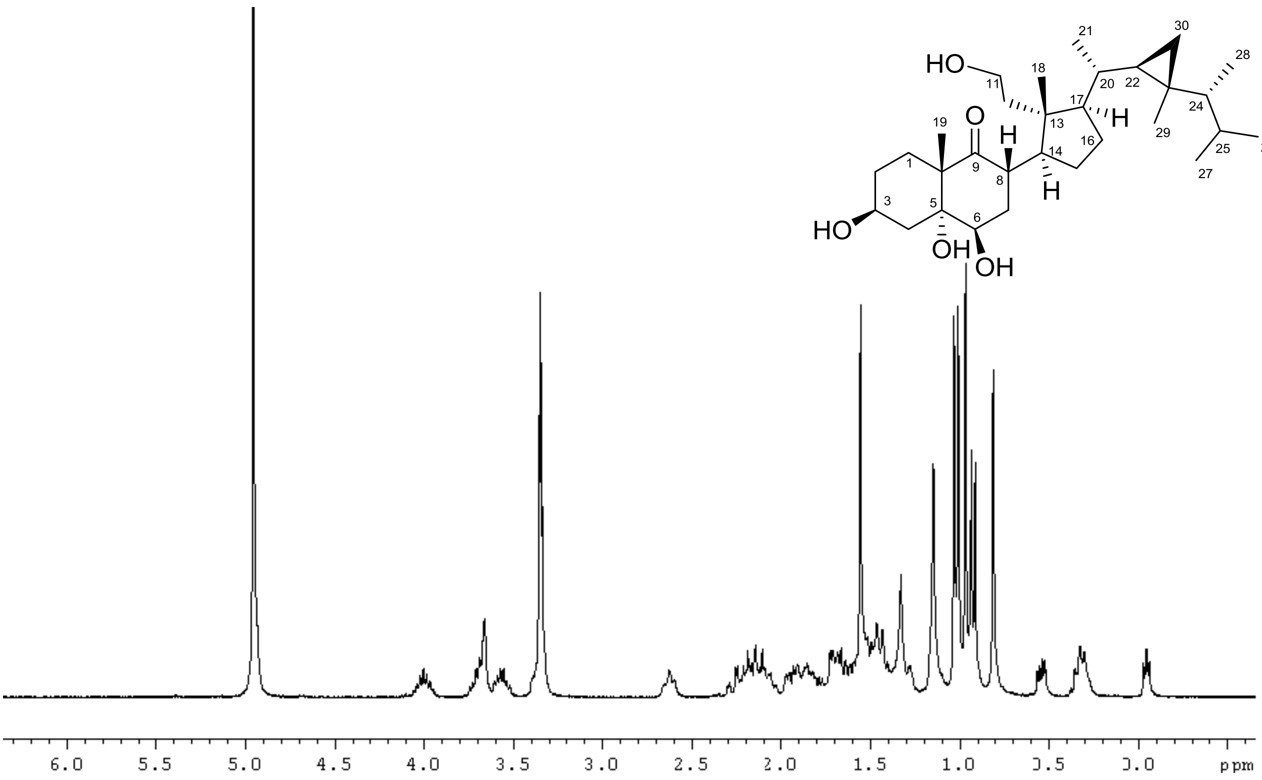

Figure S1. ^{1}H NMR spectrum of 3β,5α,6β-trihydroxy-9-oxo-9,11-secogorgostan-11-ol (**1**) in MeOH-d_4

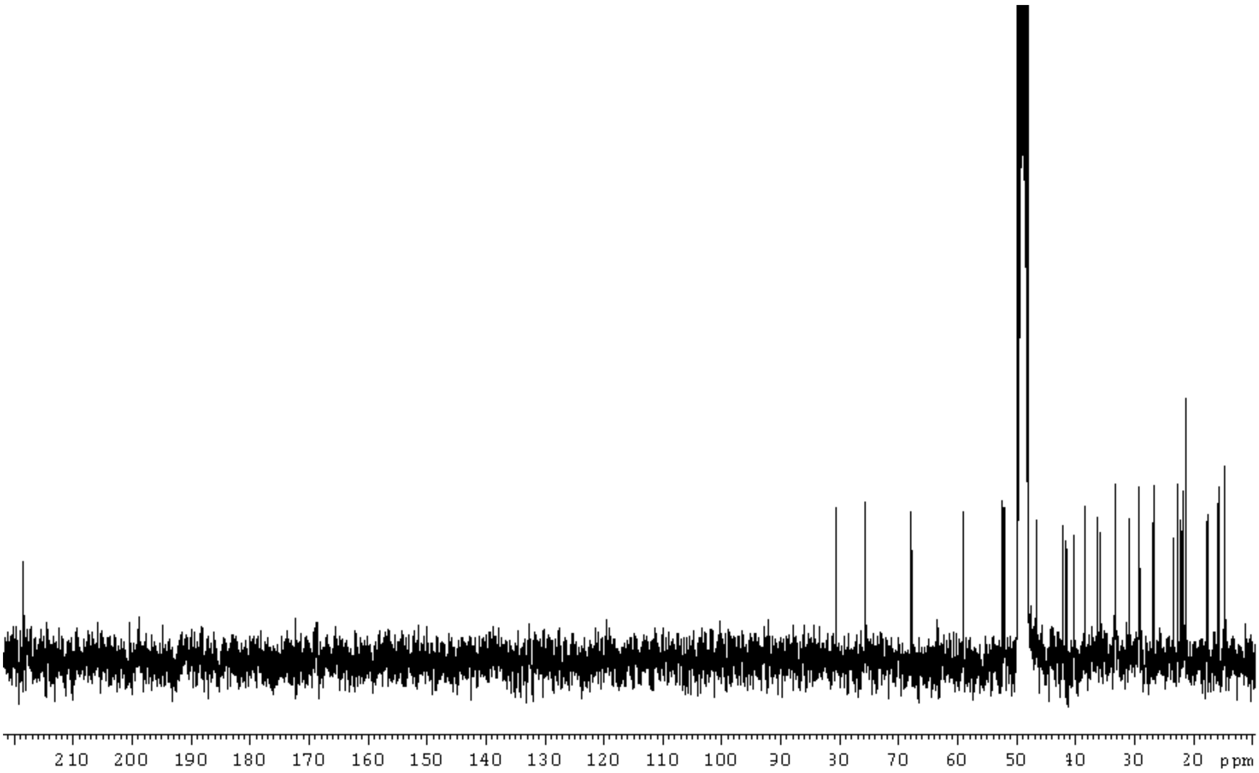

Figure S2. ^{13}C NMR spectrum of 3β,5α,6β-trihydroxy-9-oxo-9,11-secogorgostan-11-ol (**1**) in MeOH-d_4

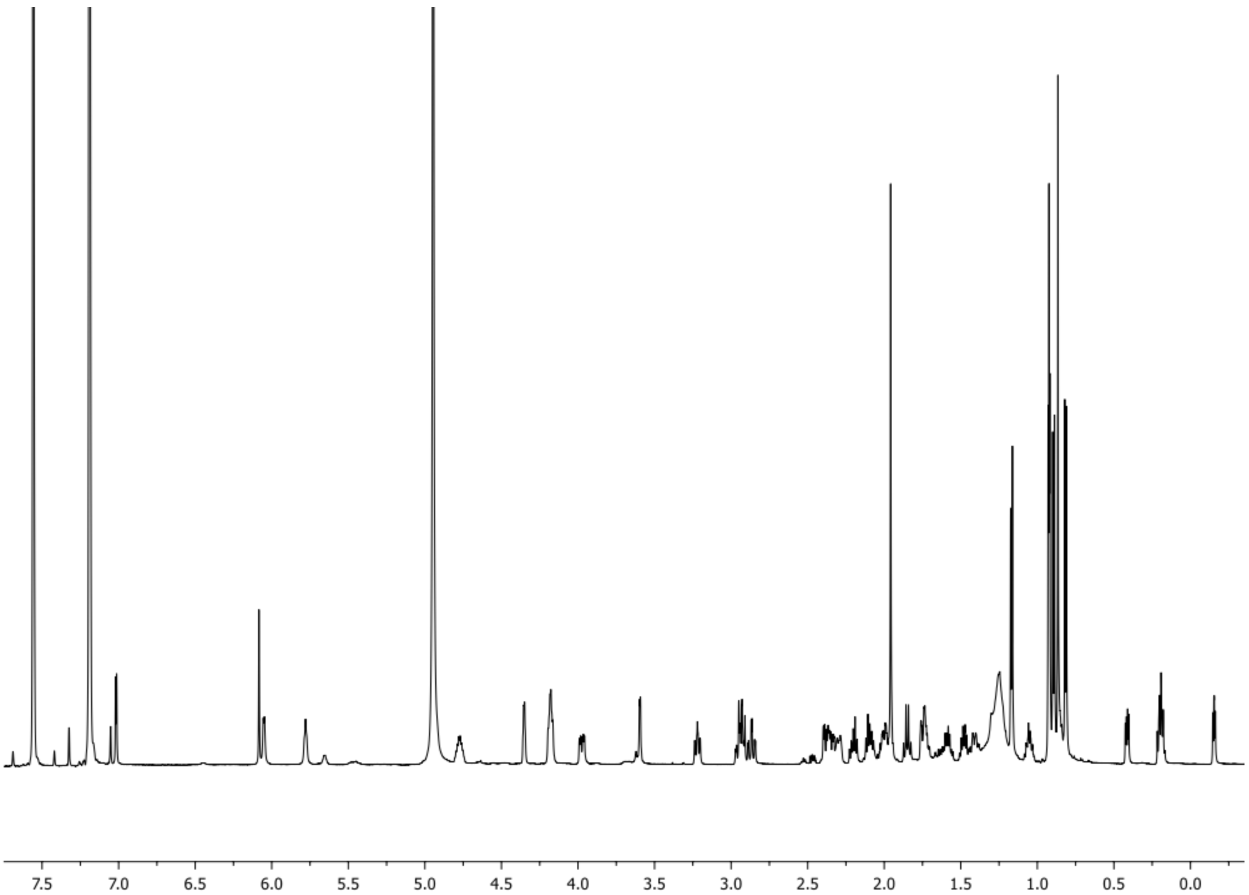

Figure S3. ^{1}H NMR spectrum of 3β,5α,6β-trihydroxy-9-oxo-9,11-secogorgostan-11-ol (**1**) in pyridine-d_5

Table S1. NMR data of 3β,5α,6β-trihydroxy-9-oxo-9,11-secogorgostan-11-ol (**1**) in MeOH-d_4

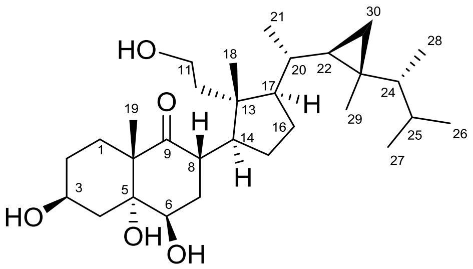

C [a]	δ_H (mult, *J* in Hz)	δ_C [b]	COSY	HMBC	NOESY
1	α 2.18, m	29.2, CH_2	H-1β, H_2-2		H-3
	β 1.31, m		H-1α, H_2-2		H_3-19
2	α 1.88, m	31.1, CH_2	H-2β, H_2-1		
	β 1.52, m		H-2α, H_2-1		
3	4.00, tt (6.0, 12.0)	68.0, CH	H_2-2, H_2-4		H-1α, H-2α, H-4α
4	β 2.15, t (12.0)	40.4, CH_2	H-3, H-4α	C-5, C-6	H_3-19
	α 1.70, m		H-3, H-4β	C-5, C-6	H-3, H-6
5		80.7, C			
6	3.66, m	75.7, CH	H_2-7		H-4α, H_2-7
7	a 2.25, m	35.9, CH_2	H-6, H-7b, H-8	C-9	
	b 1.95, m		H-6, H-7a, H-8	C-9	
8	3.36, m	38.6, CH	H_2-7, H-14	C-9	H-14, H_3-18, H_3-19
9		218.4, C			
10		52.6, C			
11	a 3.69, m	59.1, CH_2	H-11b, H_2-12		
	b 3.56, dt (5.5, 10.6)		H-11a, H_2-12		
12	a 1.82, m	41.7, CH_2	H_2-11, H-12b		
	b 1.55, m		H_2-11, H-12a		
13		46.8, C			
14	2.64, m	42.2, CH	H-8, H_2-15		H-11b, H-17
15	1.46, m	23.4, CH_2	H_2-16		
16	a 2.08, m	29.3, CH_2	H_2-15, H-16b		
	b 1.44, m		H_2-15, H-16a		H_3-29
17	1.71, m	52.1, CH	H_2-16, H-20		
18	0.81, s	17.7, CH_3		C-12, C-13, C-14, C-17	H-4β, H-8, H_3-21
19	1.56, s	21.4, CH_3		C-1, C-5, C-9, C-10	H-8
20	1.12, m	36.3, CH	H-17, H_3-21, H-22		
21	1.15, brs	21.4, CH_3	H-20	C-17, C-20, C-22	H_3-18, H-22, H-30a
22	0.32, m	33.3, CH	H-20, H_2-30		
23		26.9, C			
24	0.34, m	52.2, CH	H-25, H_3-28	C-23, C-25	
25	1.62, m	33.4, CH	H_3-26, H_3-27		
26	0.93, d (7.0)	21.9, CH_3	H-25	C-24, C-25, C-27	
27	1.02, d (6.6)	22.7, CH_3	H-25	C-24, C-25, C-26	
28	1.02, d (6.6)	15.8, CH_3	H-24	C-23, C-24, C-25	
29	0.97, s	14.7, CH_3	H_2-30	C-22, C-23, C-24, C-30	H-30b
30	a 0.54, dd (4.4, 9.3)	22.2, CH_2	H-22, H_3-29, H-30b	C-22, C-23, C-24, C-29	
	b -0.05, dd (4.4, 5.7)		H-22, H_3-29, H-30a	C-22, C-23, C-24, C-29	

[a] All assignments are based on extensive 1D and 2D NMR measurements (COSY, HSQC, HMBC).

[b] Multiplicities determined by DEPT.

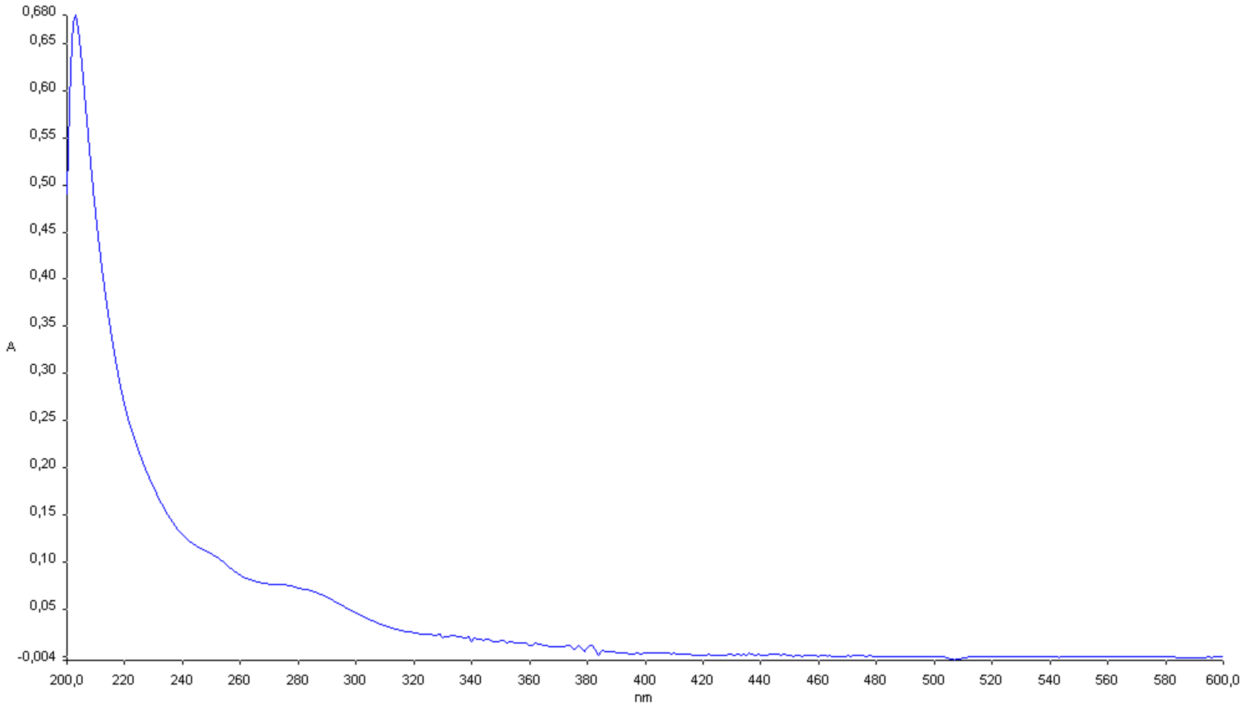

Figure S4. UV spectrum of 3β,5α,6β-trihydroxy-9-oxo-9,11-secogorgostan-11-ol (1) in MeOH

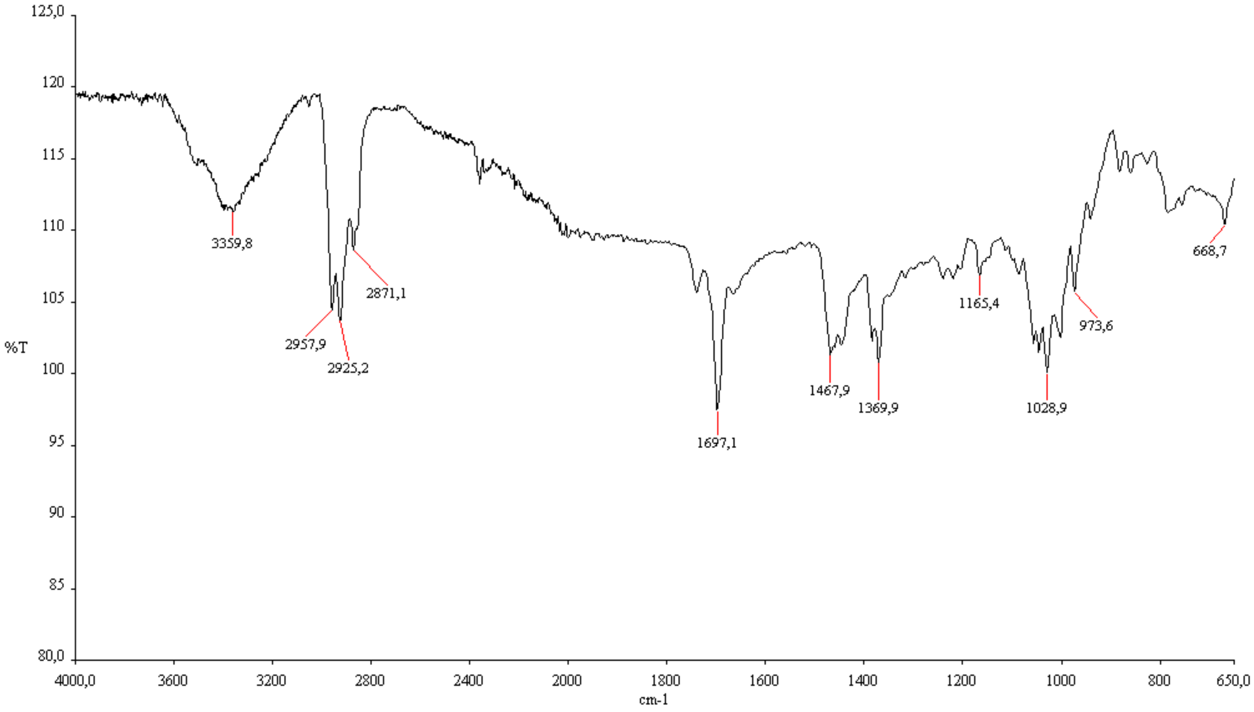

Figure S5. IR spectrum of 3β,5α,6β-trihydroxy-9-oxo-9,11-secogorgostan-11-ol (**1**)

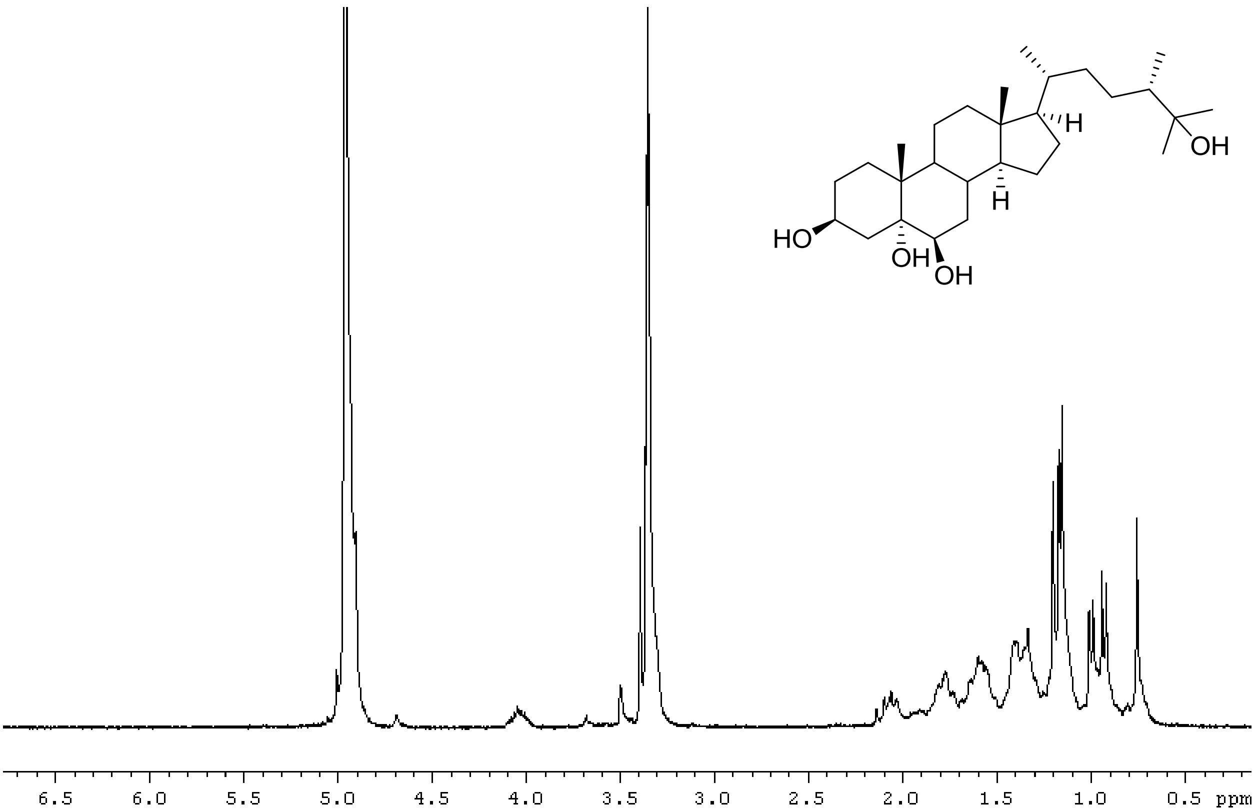

Figure S6. ^{1}H NMR spectrum of steroid **2** in MeOH-d_4

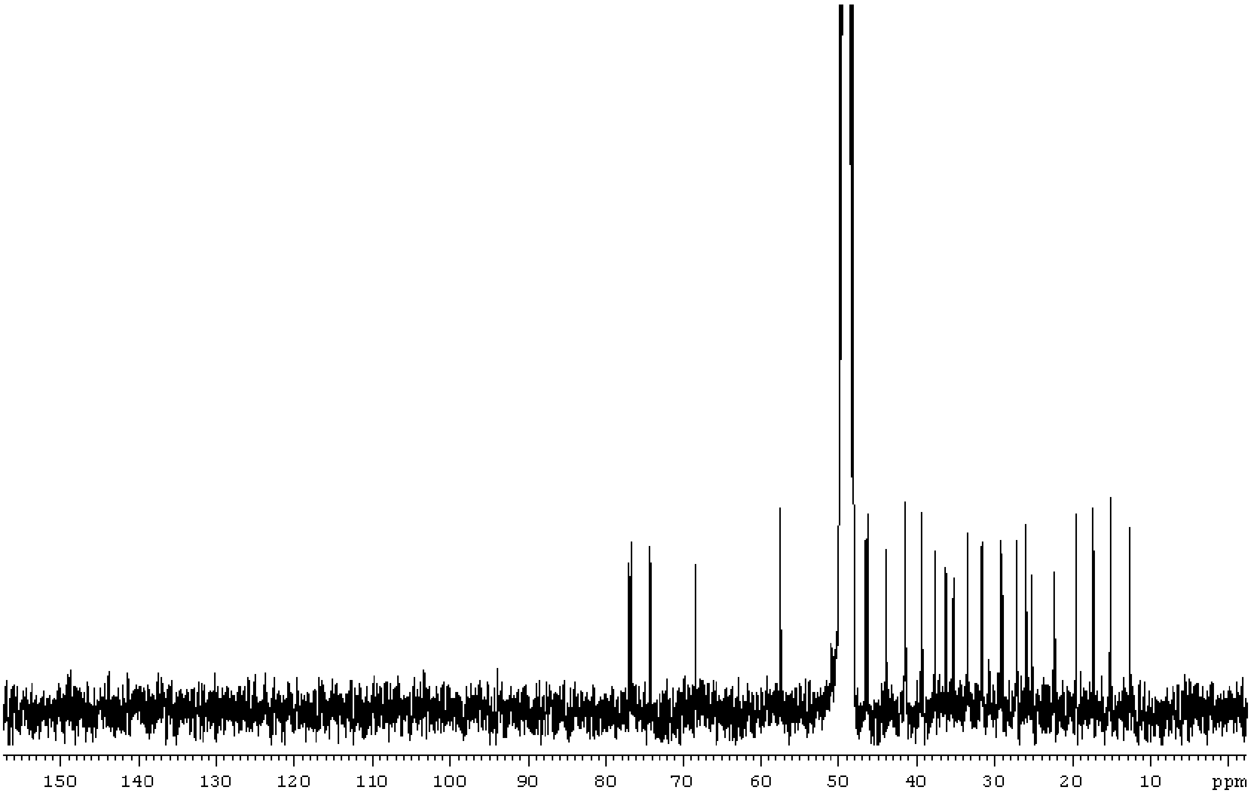

Figure S7. ^{13}C NMR spectrum of steroid **2** in MeOH-d_4

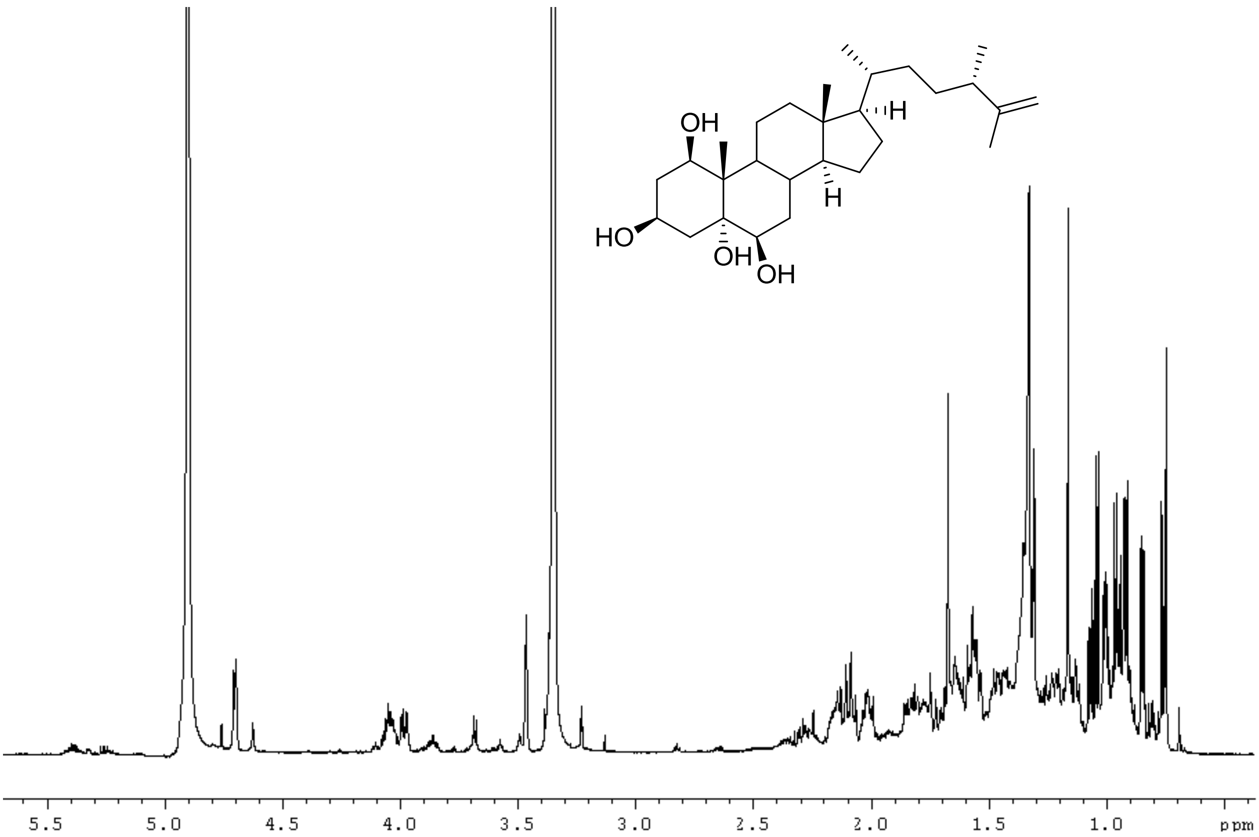

Figure S8. ^{1}H NMR spectrum of steroid **3** in MeOH-d_4

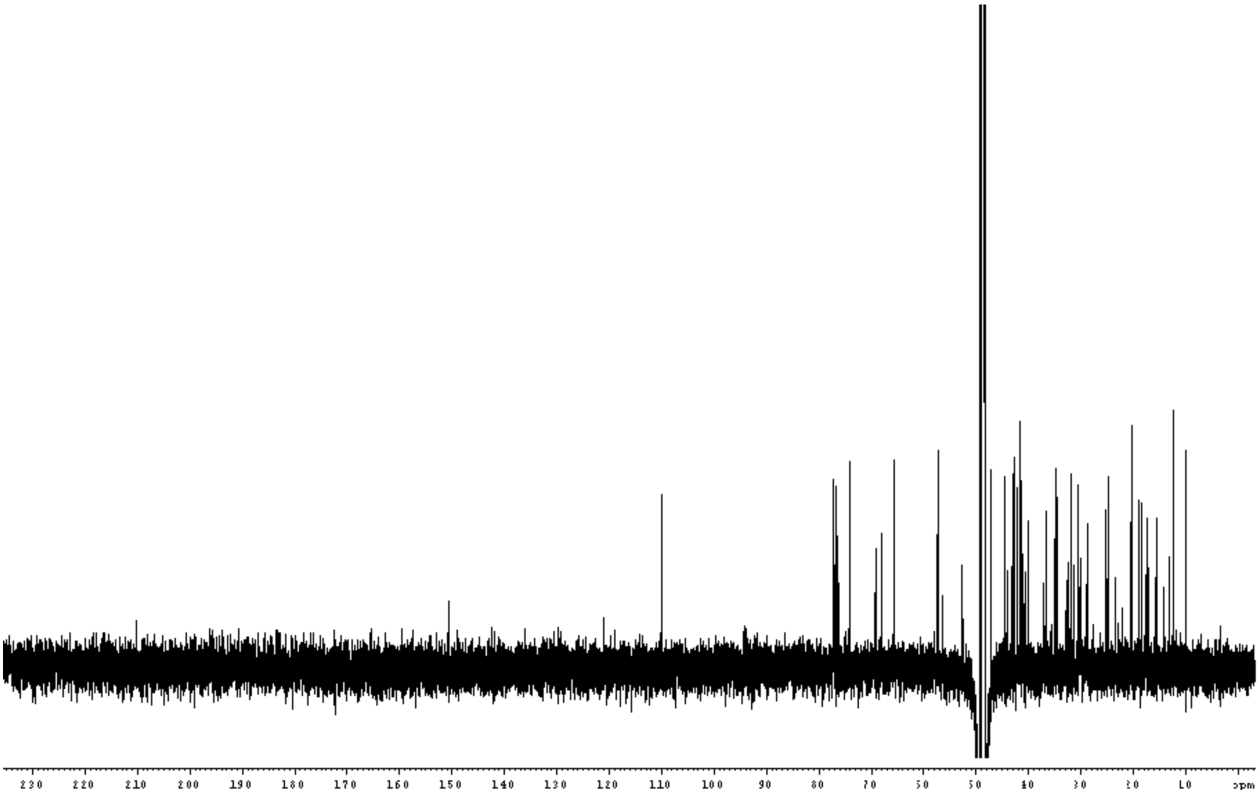

Figure S9. ^{13}C NMR spectrum of steroid **3** in MeOH-d_4

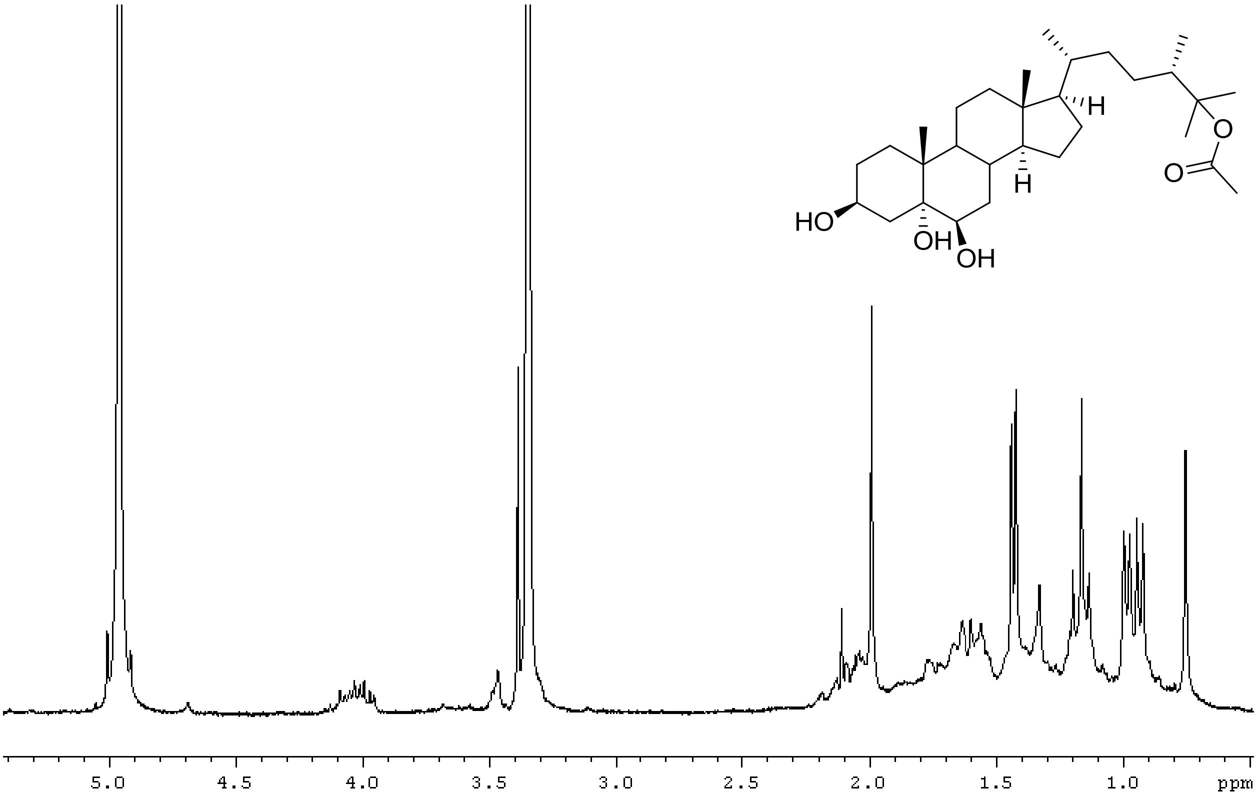

Figure S10. ^{1}H NMR spectrum of steroid **4** in MeOH-d_4

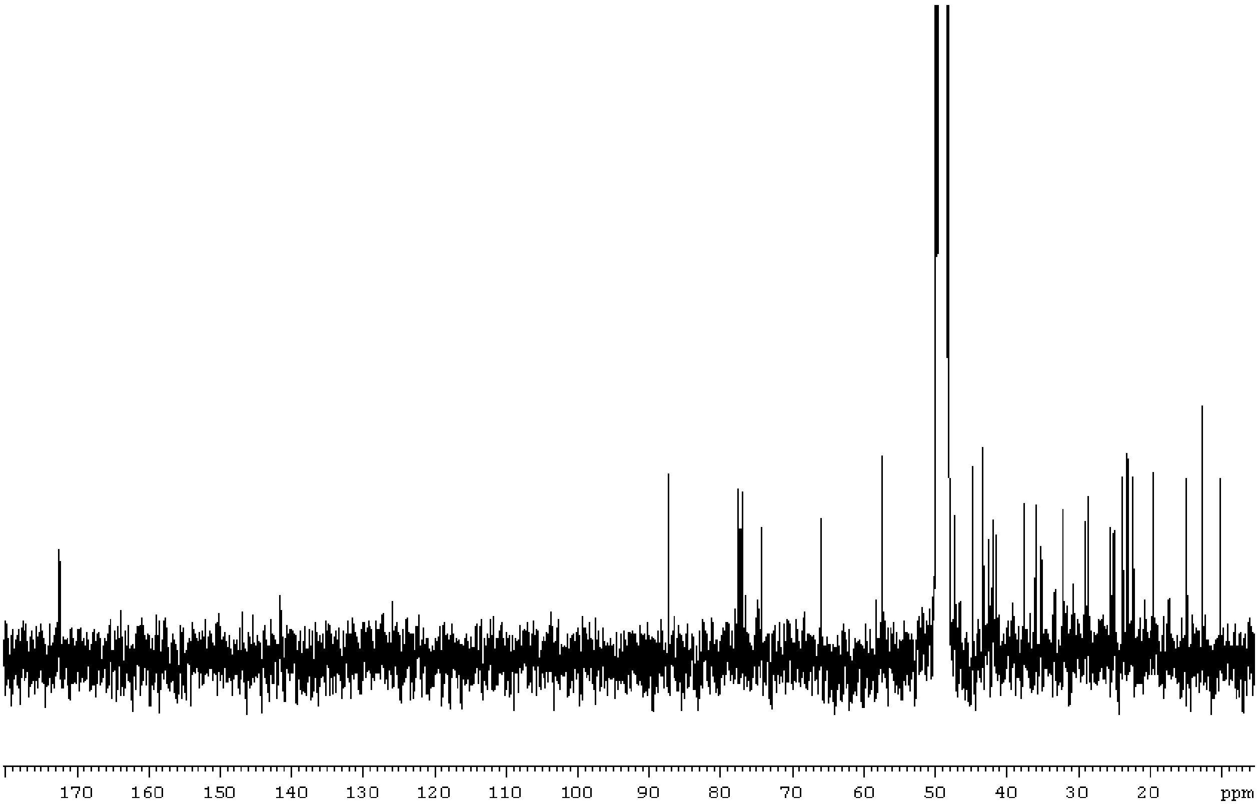

Figure S11. ^{13}C NMR spectrum of steroid **4** in MeOH-d_4

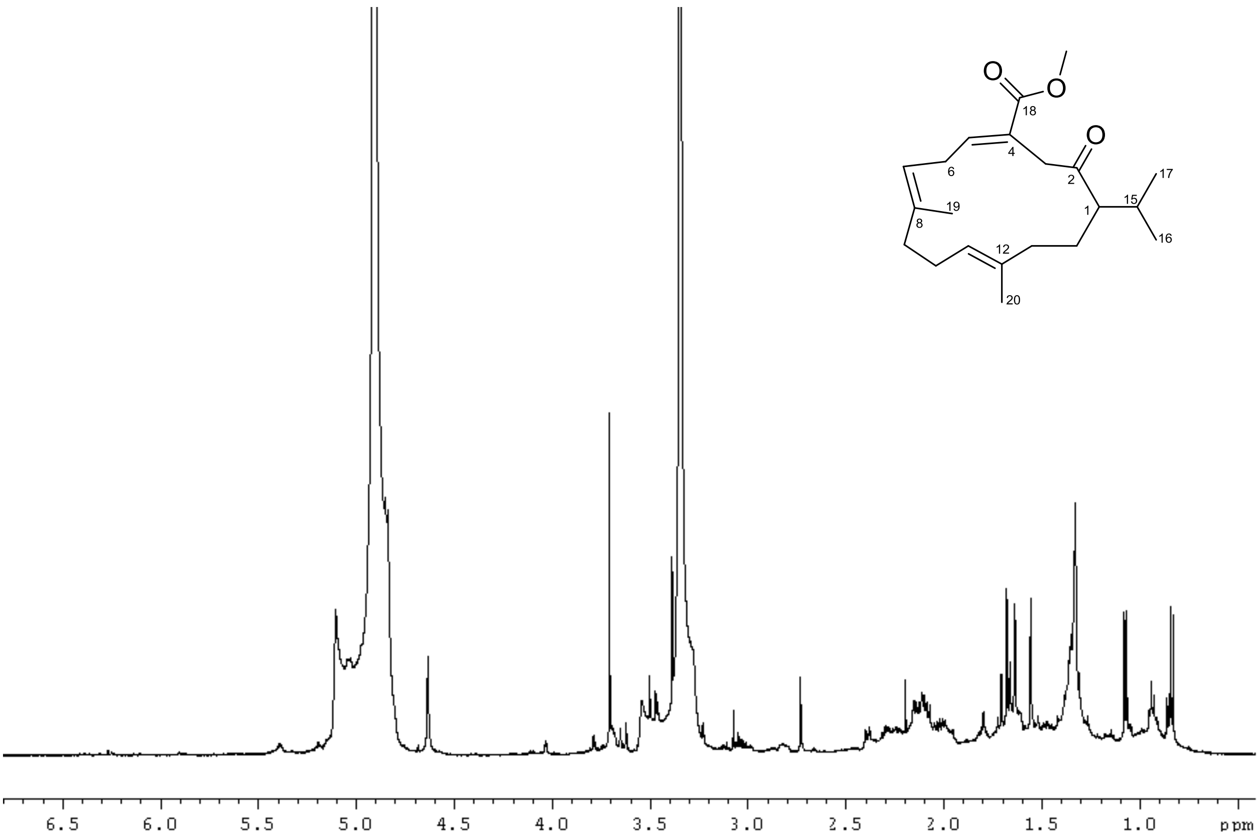

Figure S12. ^{1}H NMR spectrum of 6,13-bisdesoxomethyl sarcoate (**5**) in MeOH-d_4

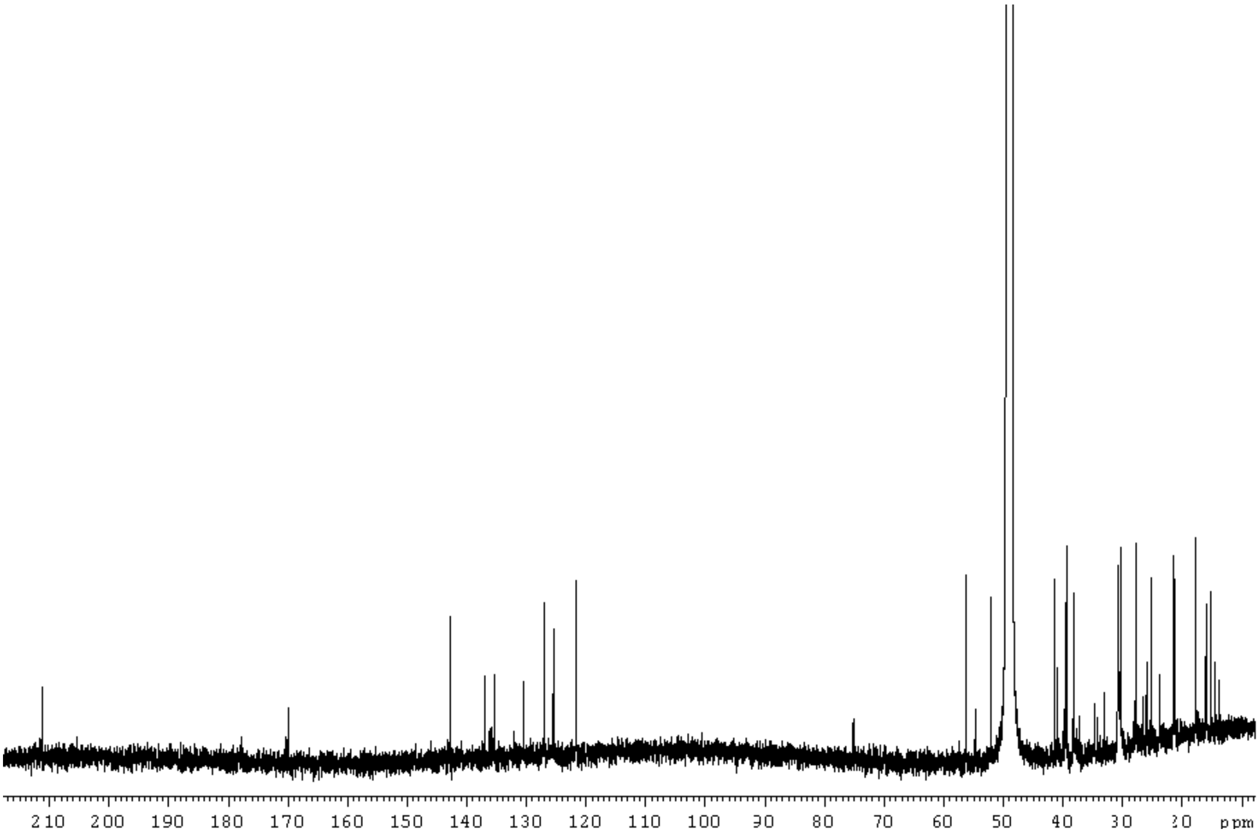

Figure S13. ^{13}C NMR spectrum of 6,13-bisdesoxomethyl sarcoate (**5**) in MeOH-d_4

Table S2. NMR data of 6,13-bisdesoxomethyl sarcoate (**5**) in MeOH-d_4

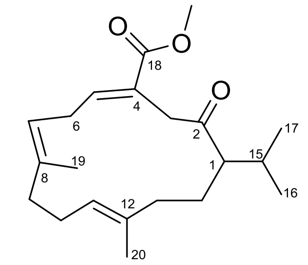

C [a]	δ_H (mult, J in Hz)	δ_C [b]	COSY	HMBC
1	2.39, dt (10.3, 3.3)	56.2, CH	H-14a, H-15	C-2, C-14, C-15, C-16, C-17
2		211.3, C		
3	a 3.64, d (17.7)	41.3, CH_2	H-3b, H-5	C-2, C-4, C-5, C-18
	b 3.49, d (17.7)		H-3a, H-5	C-2, C-4, C-5, C-18
4		130.5, C		
5	7.08 ddd (1.5, 7.0, 8.2)	142.8, CH	H_2-6, H-3b	C-18
6	a 3.05, m	27.8, CH_2	H-5, H-6b, H-7	C-4, C-5, C-7, C-8
	b 2.83, m		H-5, H-6b, H-7	C-4, C-5, C-7, C-8
7	5.05, m	121.6, CH	H-3a, H_2-6	
8		136.9, C		
9	2.16, m	39.5, CH	H_2-6	
10	a 2.26, m	25.1, CH_2	H_2-9, H-10b, H-11	
	b 2.14, m		H_2-9, H-10a, H-11	
11	4.81, m	127.0, CH	H_2-10, H_3-20	
12		135.4, C		
13	a 2.11, m	38.2, CH_2	H-13b, H-14b	C-1, C-12, C-14
	b 1.99, m		H-13a, H-14b	C-1, C-12, C-14
14	a 1.81, m	21.2, CH_2	H-14b	C-1, C-2, C-12, C-13, C-15, C-16, C-17
	b 1.47, m		H_2-13, H-14a	
15	2.31, m	30.4, CH	H-1, H-16, H-17	C-2
16	0.84, d (7.0)	17.8, CH_3	H-15	C-1, C-15, C-17
17	1.08, d (7.0)	21.4, CH_3	H-15	C-1, C-15, C-16
18		170.0, C		
19	1.68, br s	16.0, CH_3	H-7	C-7, C-8, C-9
20	1.56, br s	15.2, CH_3	H-11	C-11, C-12, C-13
OMe	3.71, s	52.1, CH_3		C-4, C-18

[a] All assignments are based on extensive 1D and 2D NMR measurements (COSY, HSQC, HMBC).
[b] Multiplicities determined by DEPT.

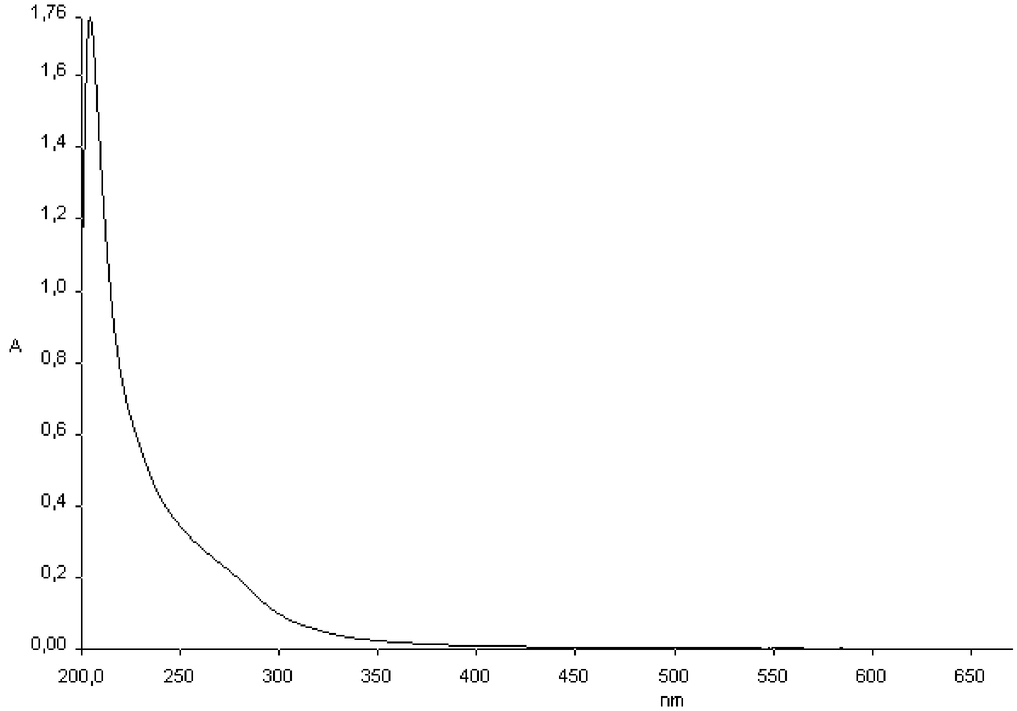

Figure S14. UV spectrum of 6,13-bisdesoxomethyl sarcoate (**5**) in MeOH

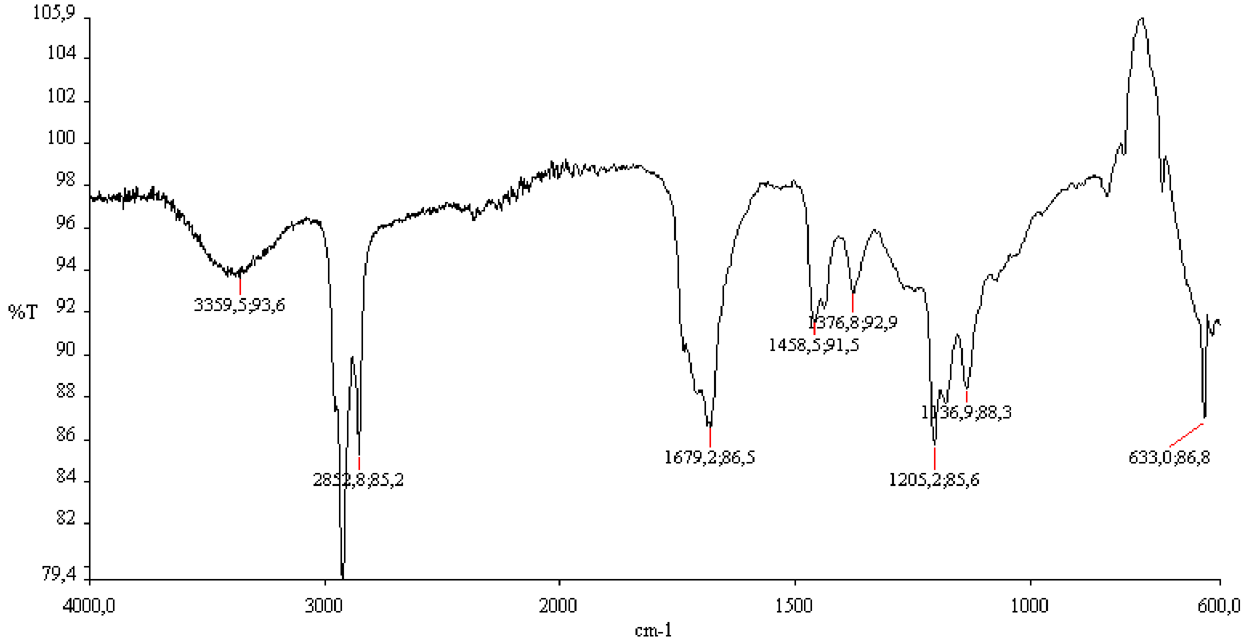

Figure S15. IR spectrum of 6,13-bisdesoxomethyl sarcoate (**5**)

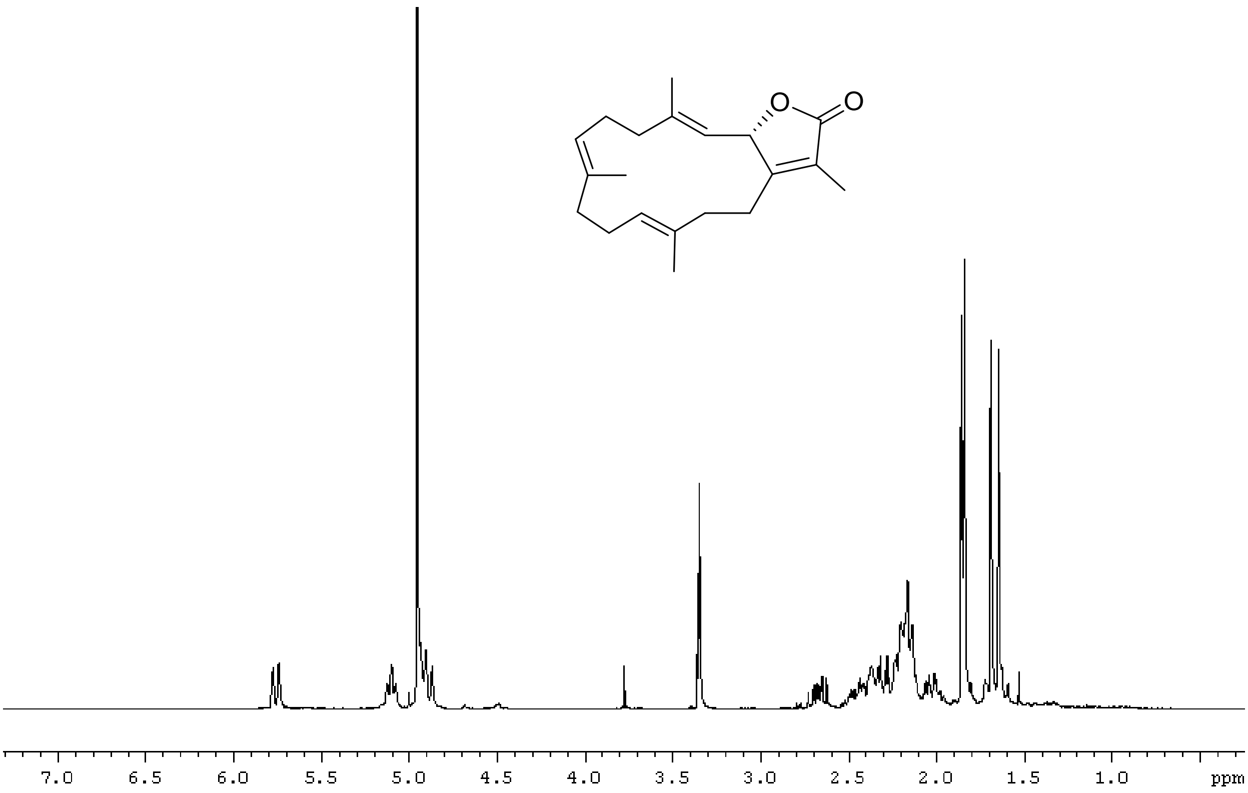

Figure S16. ^{1}H NMR spectrum of sarcophytonin B (**6**) in MeOH-d_4

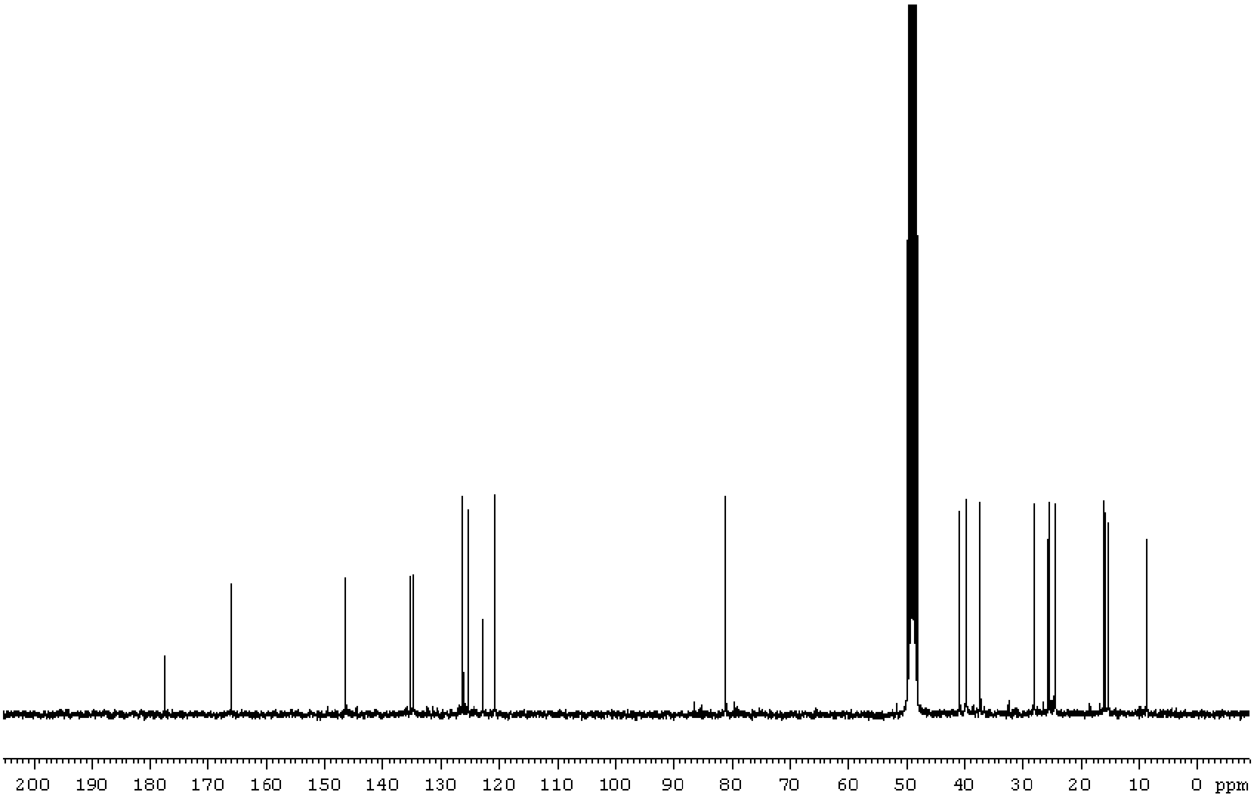

Figure S17. ^{13}C NMR spectrum of sarcophytonin B (**6**) in MeOH-d_4

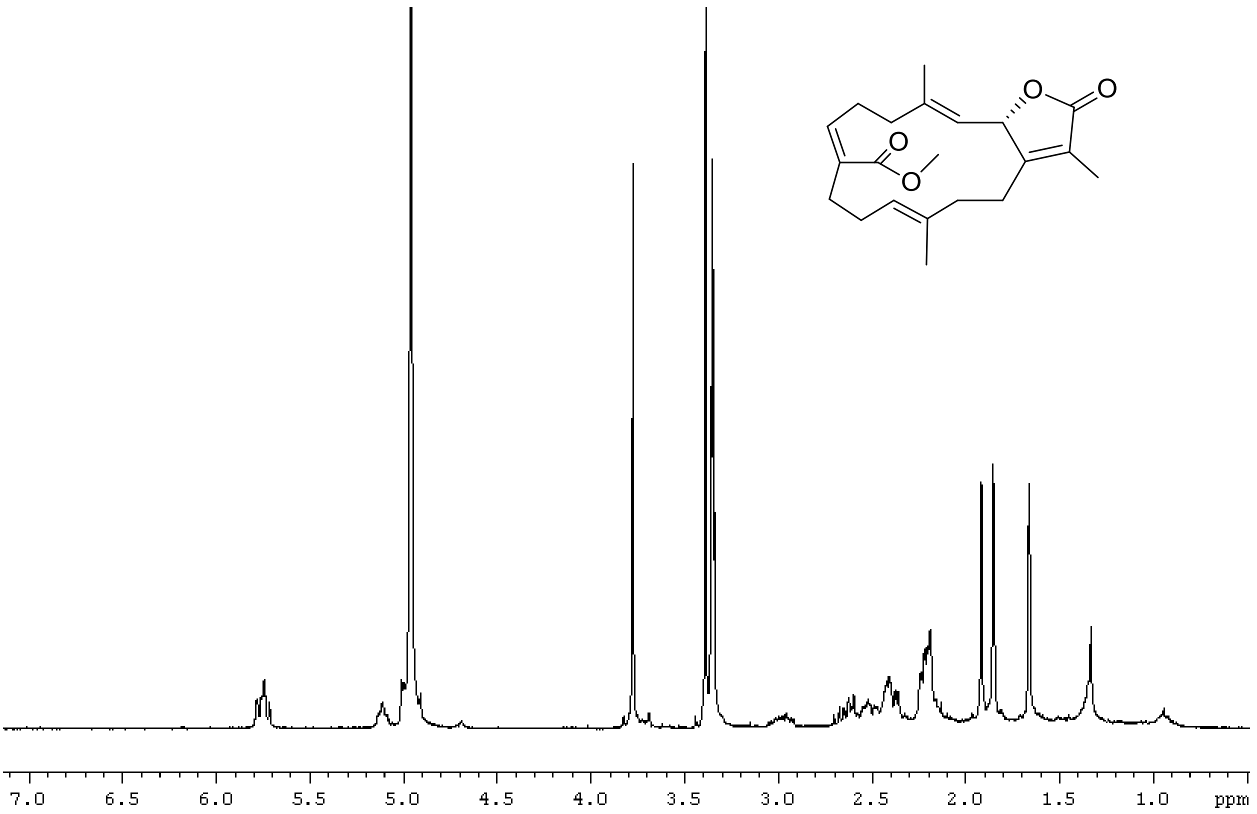

Figure S18. ^{1}H NMR spectrum of compound **7** in MeOH-d_4

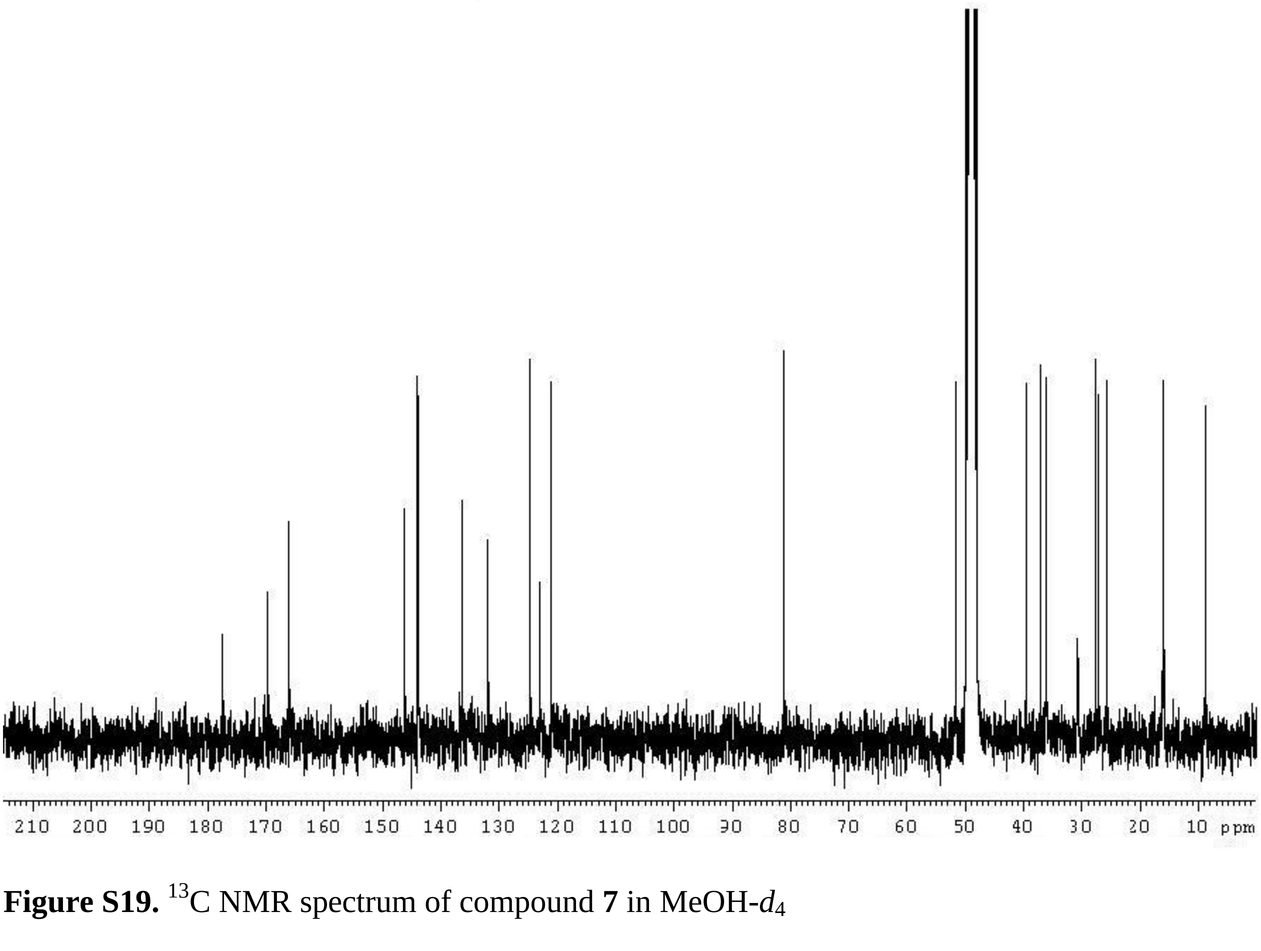

Figure S19. ^{13}C NMR spectrum of compound **7** in MeOH-d_4

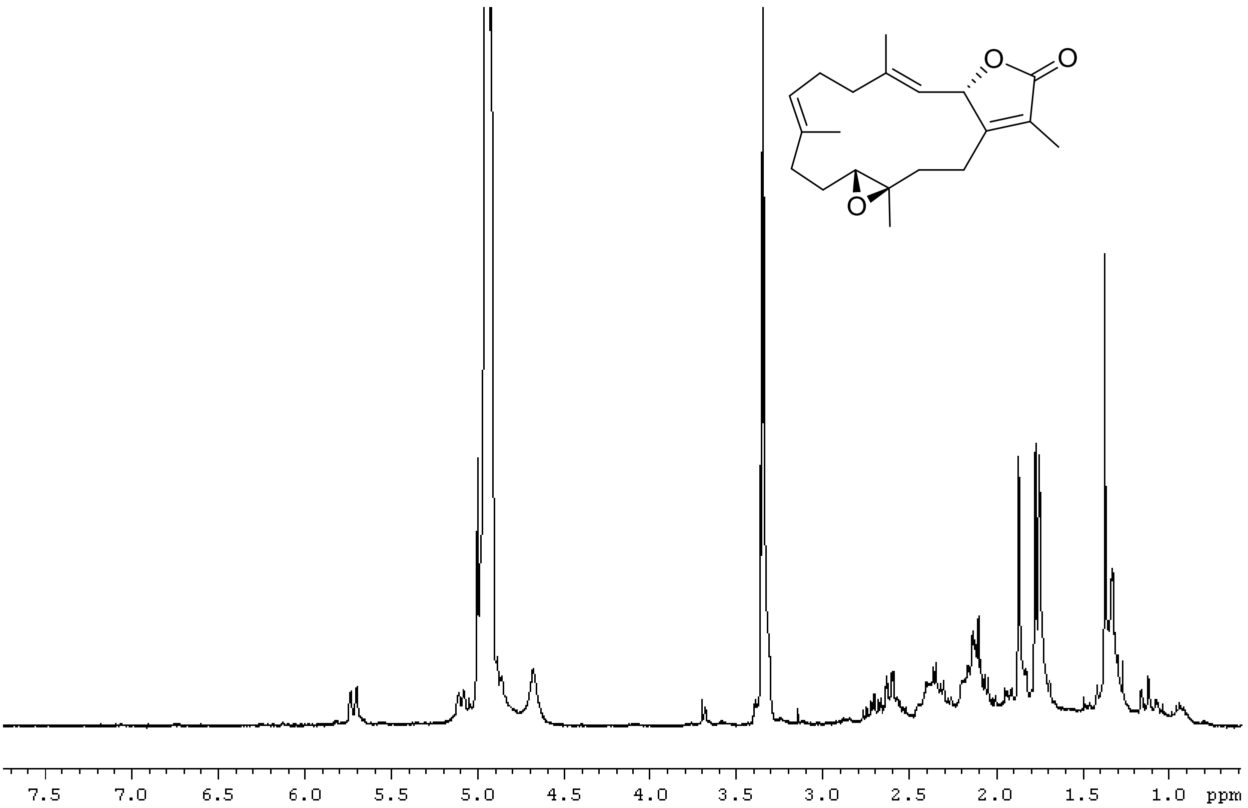

Figure S20. ^{1}H NMR spectrum of (2*S*)-isosarcophine (**8**) in MeOH-d_4

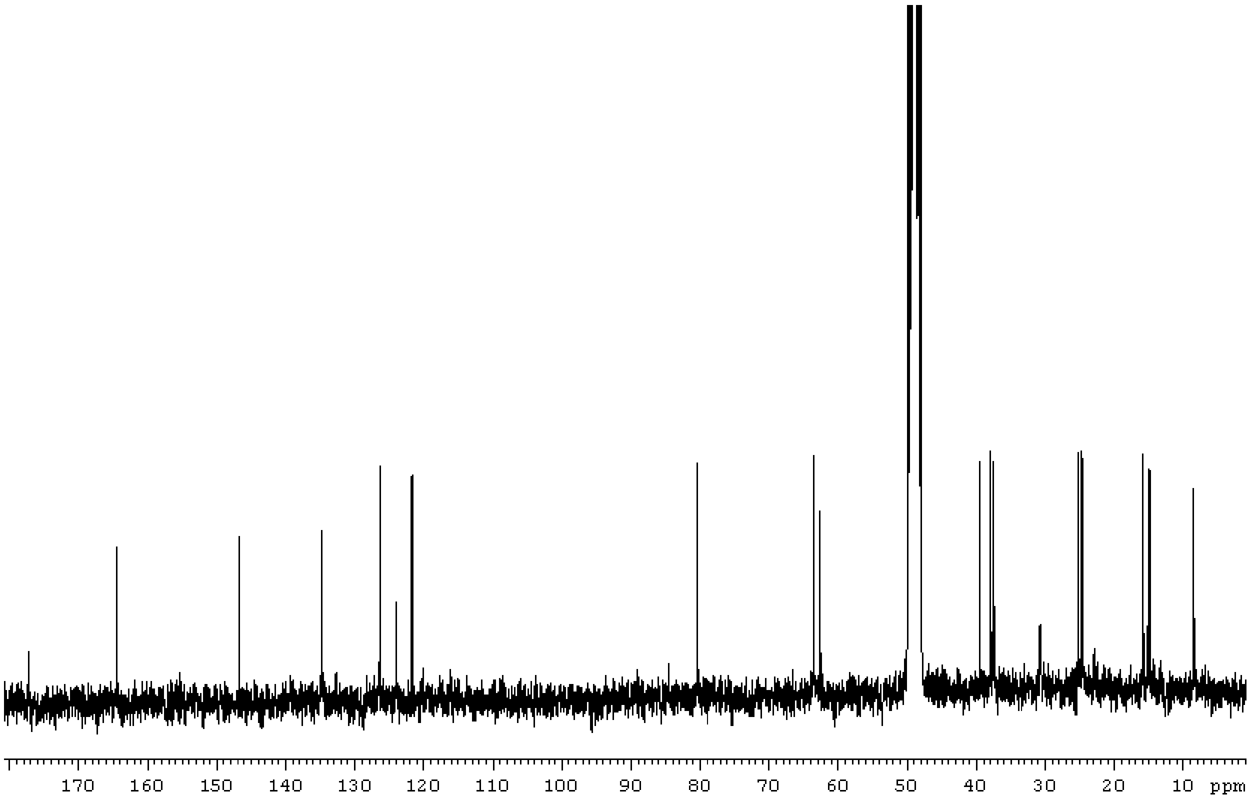

Figure S21. ^{13}C NMR spectrum of (2*S*)-isosarcophine (**8**) in MeOH-d_4

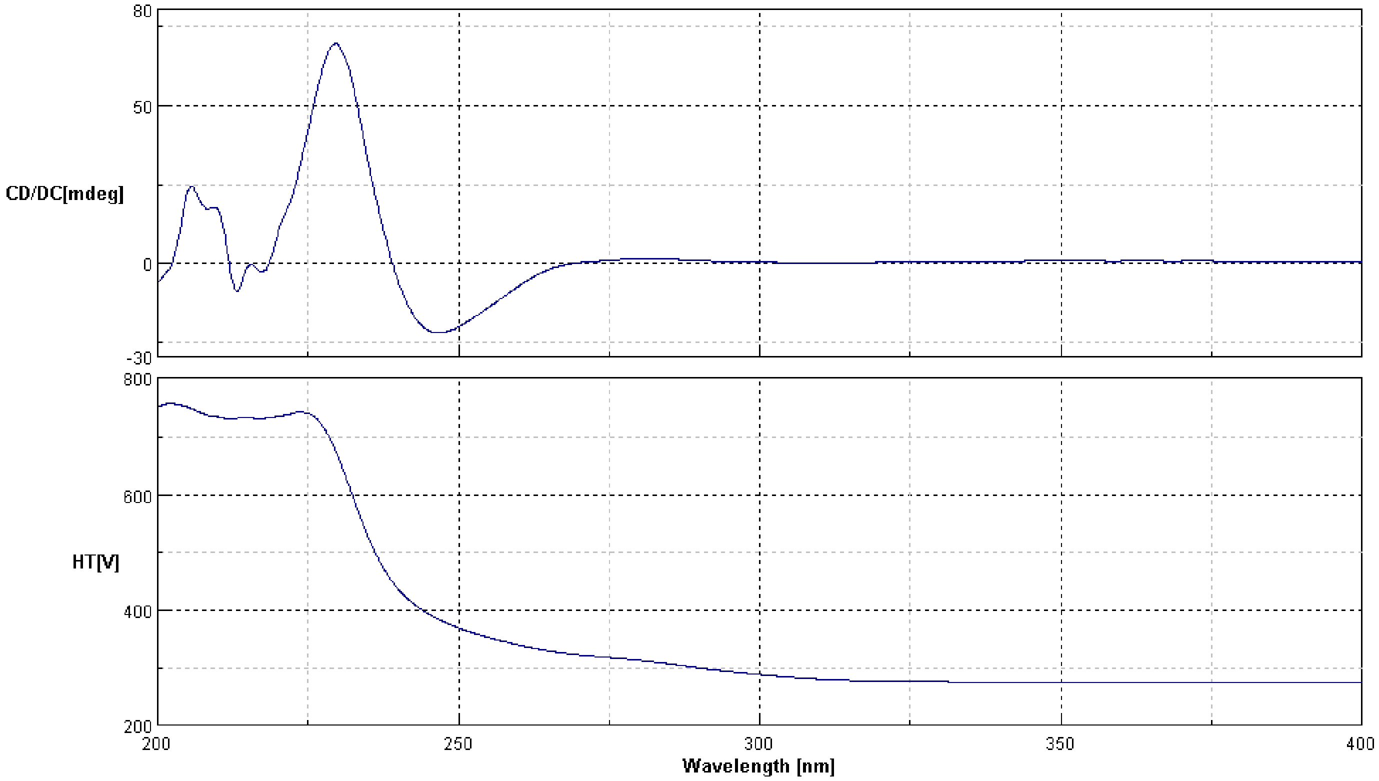

Figure S22. ECD spectrum of (2*S*)-isosarcophine (**8**) in MeOH

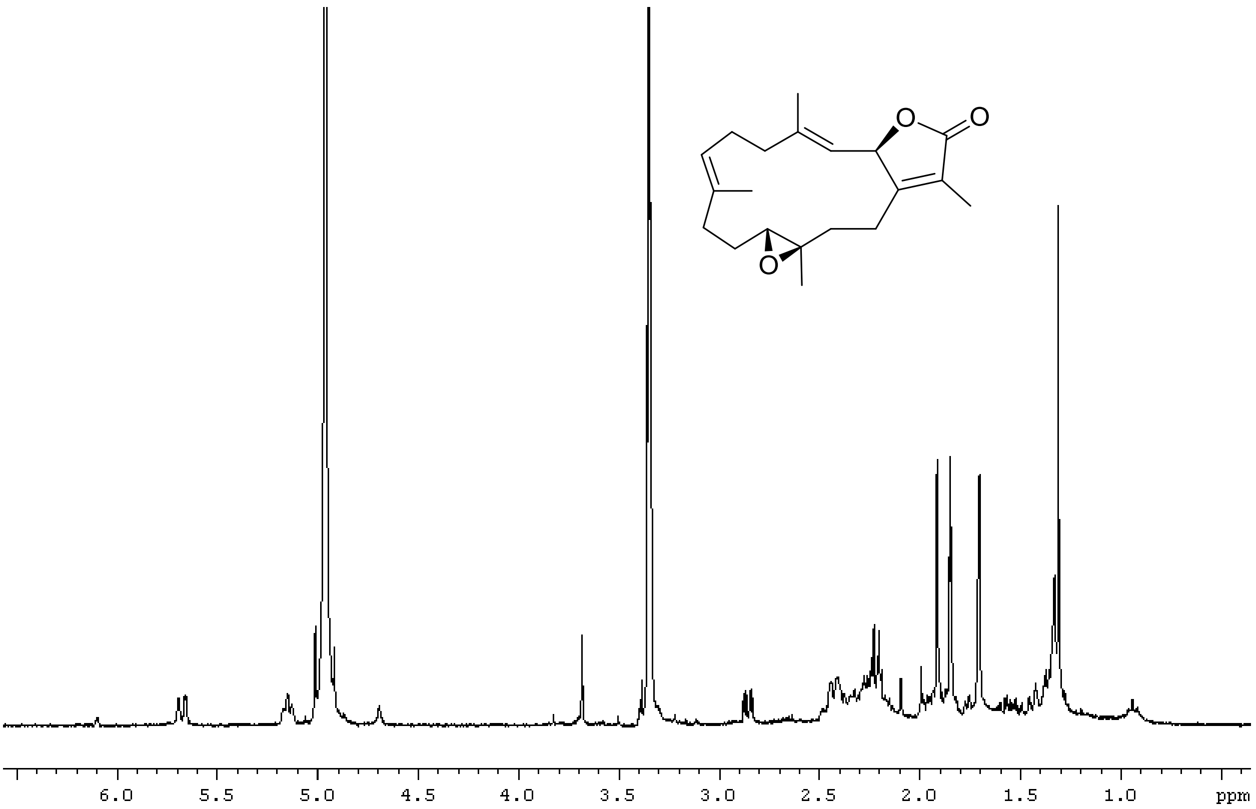

Figure S23. ^{1}H NMR spectrum of (2*R*)-isosarcophine (**9**) in MeOH-d_4

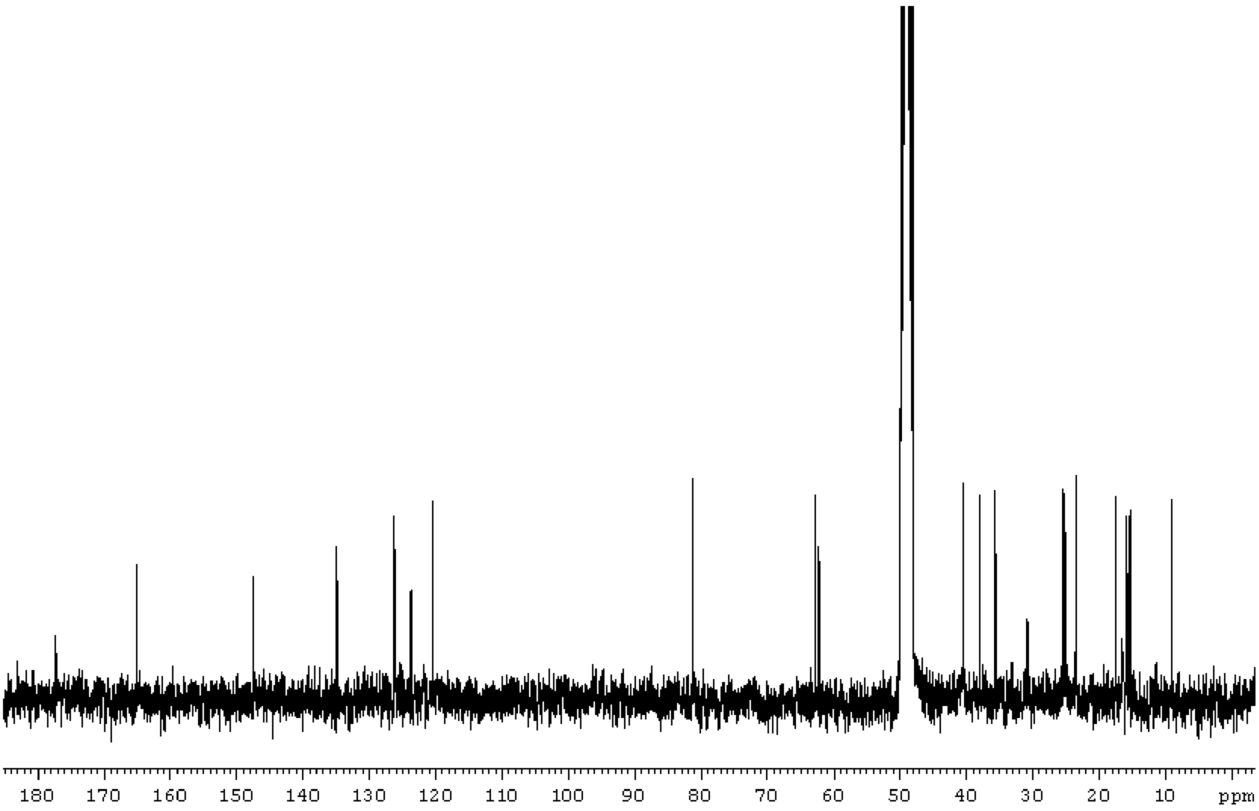

Figure S24. ^{13}C NMR spectrum of (2*R*)-isosarcophine (**9**) in MeOH-d_4

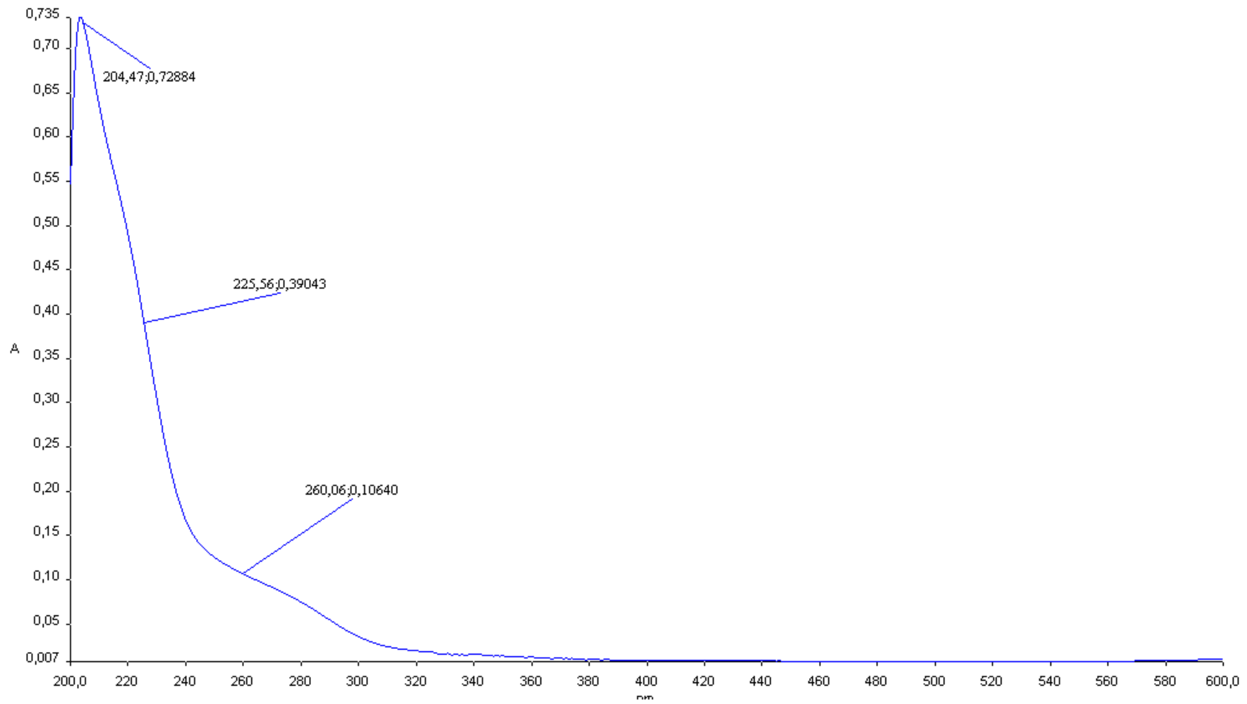

Figure S25. UV spectrum of (2*R*)-isosarcophine (**9**)

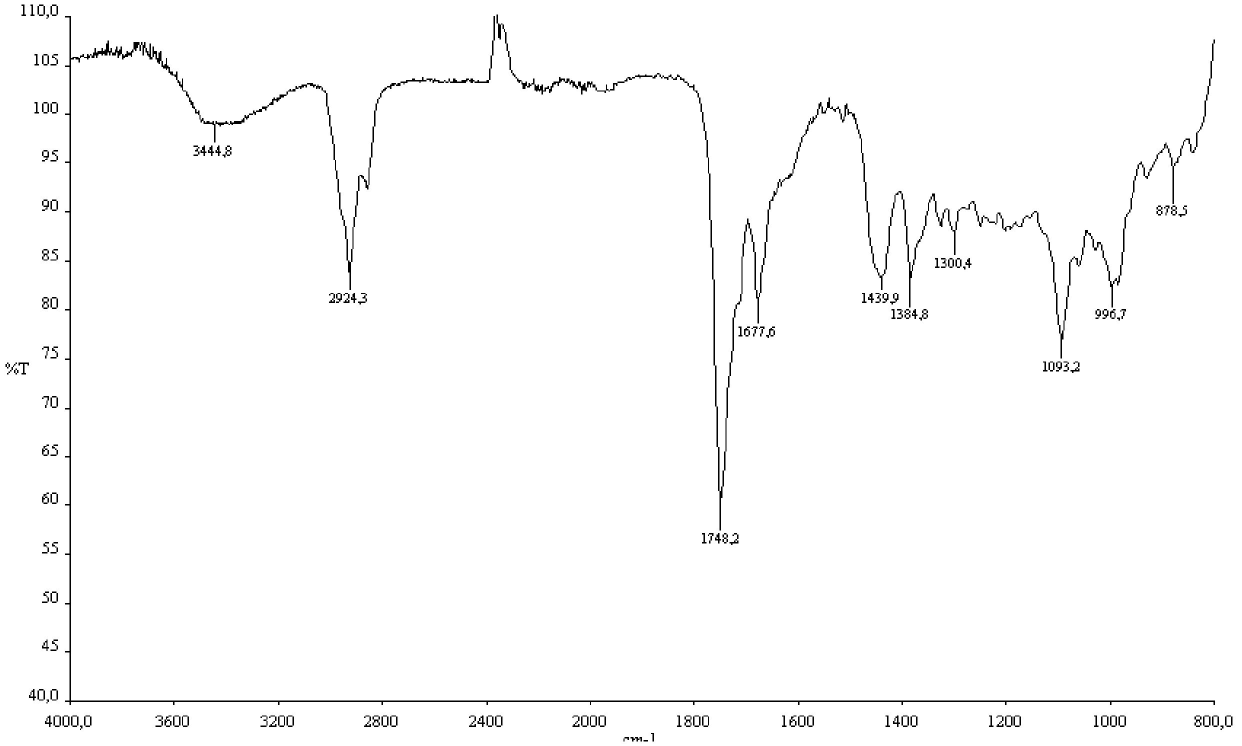

Figure S26. IR spectrum of (2*R*)-isosarcophine (**9**)

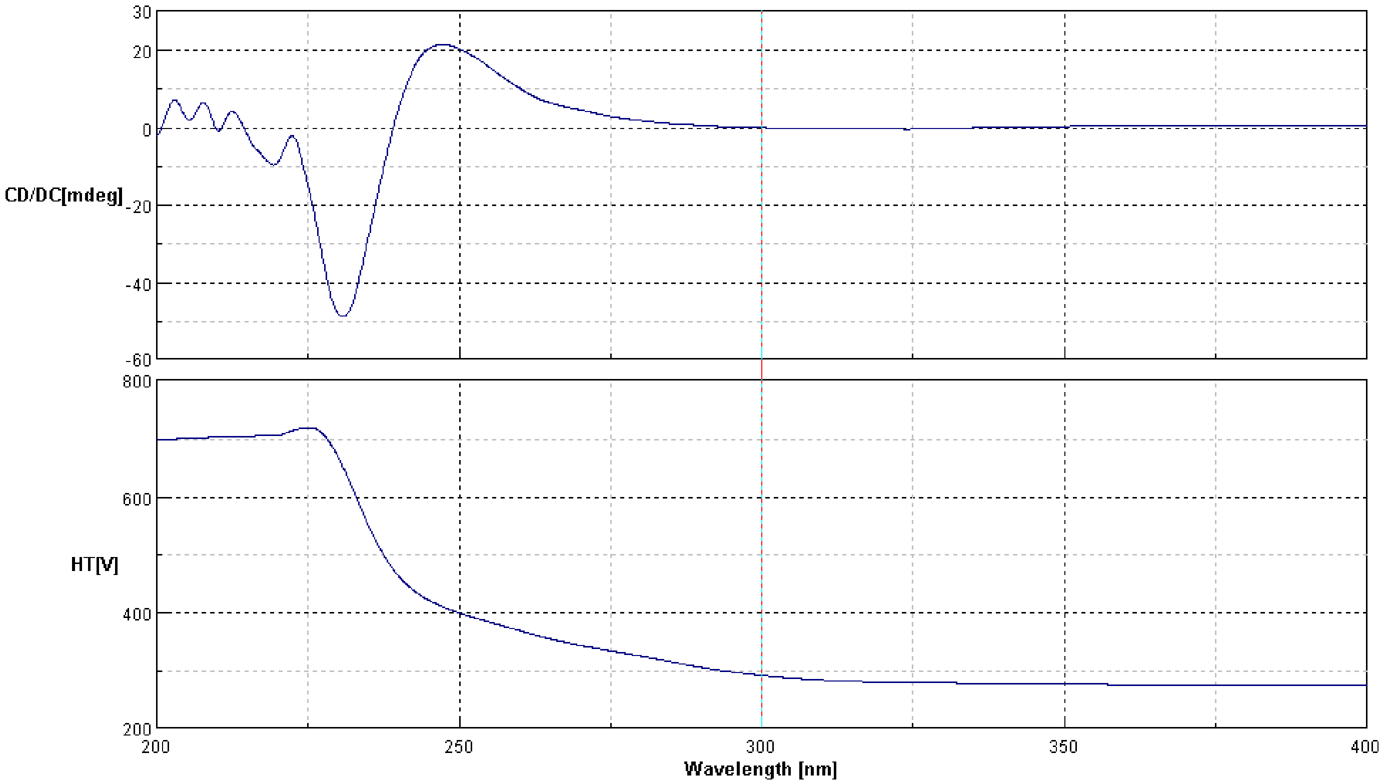

Figure S27. ECD spectrum of (2*R*)-isosarcophine (**9**) in MeOH

Table S3. ^{1}H and ^{13}C NMR data of 2*S*-isosarcophine (**8**) and 2*R*-isosarcophine (**9**) in MeOH-d_4

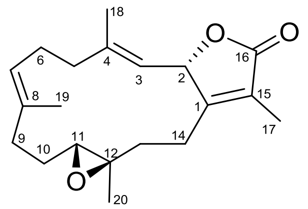

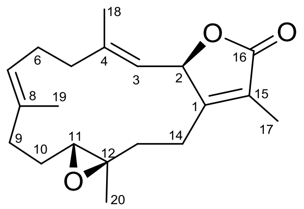

	2*S*-isosarcophine (**8**)		2*R*-isosarcophine (**9**)	
C [a]	δ_H (mult, *J* in Hz)	δ_C [b]	δ_H (mult, *J* in Hz)	δ_C [b]
1		164.3, C		165.0, C
2	5.72, dd (1.5, 9.9)	80.4, CH	5.67, dd (1.9, 9.9)	81.3, CH
3	4.91, d (9.9)	121.7, CH	4.95, d (9.9)	120.4, CH
4		146.6, C		147.6, C
5	a 2.41, m<br>b 2.30, m	39.5, CH_2	a 2.41, m<br>b 2.22, m	40.5, CH_2
6	a 2.57, m<br>b 2.18, m	25.2, CH_2	a 2.42, m<br>b 2.32, m	25.5, CH_2
7	5.10, br d (9.6)	126.4, CH	5.15, m	126.2, CH
8		134.8, C		135.0, C
9	a 2.36, m<br>b 2.12, m	37.6, CH_2	a 2.27, m<br>b 2.17, m	37.9, CH_2
10	a 2.14, m<br>b 1.34, m	24.6, CH_2	a 1.93, m<br>b 1.55, m	25.1, CH_2
11	2.61, dd (2.5, 11.0)	63.6, CH	2.86, dd (3.5, 8.8)	62.8, CH
12		62.7, C		62.3, C
13	a 2.12, m<br>b 1.13, m	38.0, CH_2	a 2.21, m<br>b 1.42, m	35.6, CH_2
14	a 2.72, m<br>b 2.16, m	24.8, CH_2	a 2.44, m<br>b 2.21, m	23.5, CH_2
15		123.9, C		123.9, C
16		177.2, C		177.3, C
17	1.87, br s	8.5, CH_3	1.85, br s	9.0, CH_3
18	1.78, br s	15.2, CH_3	1.91, br s	15.8, CH_3
19	1.76, br s	14.8, CH_3	1.71, br s	15.3, CH_3
20	1.37, s	15.9, CH_3	1.31, s	17.5, CH_3

[a] All assignements are based on extensive 1D and 2D NMR measurements (COSY, HSQC, HMBC).
[b] Multiplicities determined by DEPT.

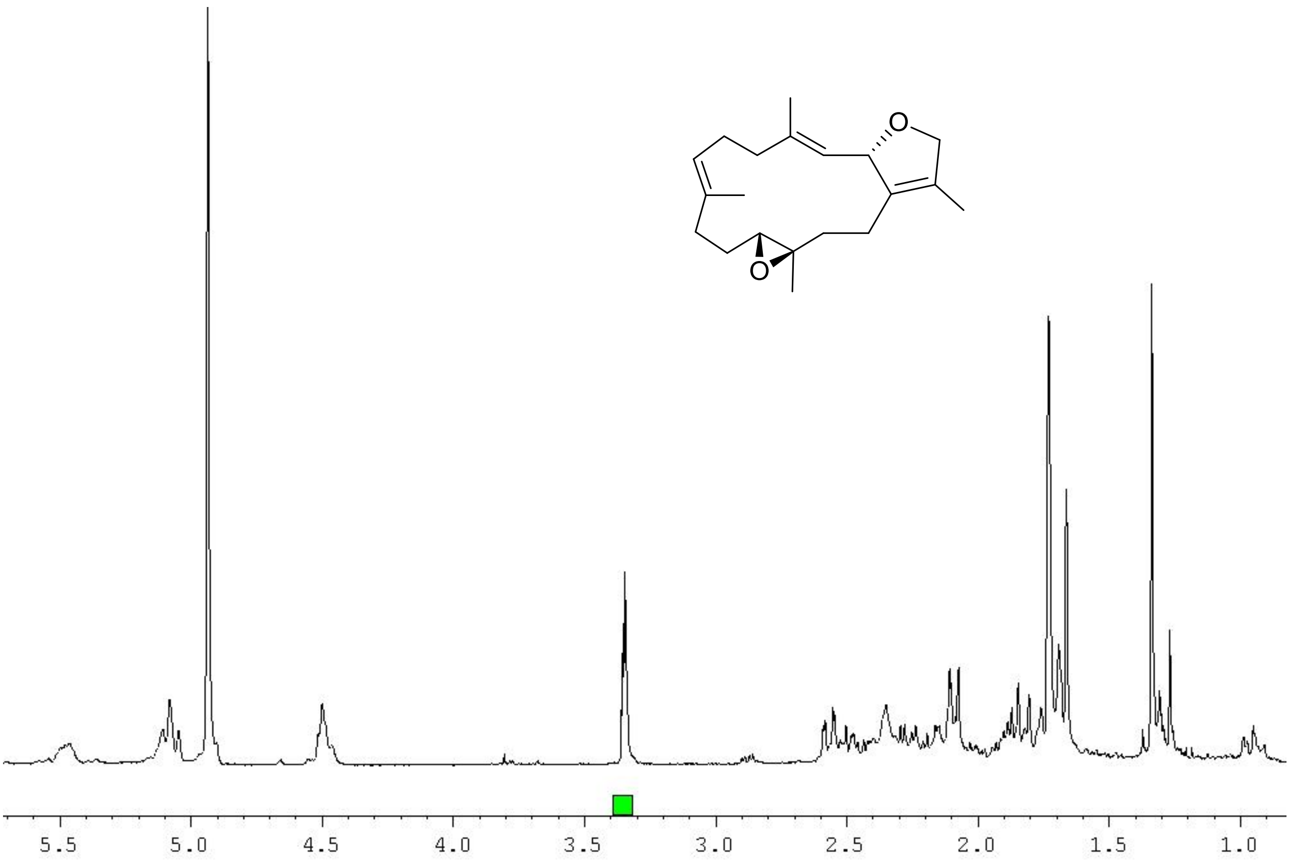

Figure S28. ^{1}H NMR spectrum of (2*S*)-isosarcophytoxide (**10**) in MeOH-d_4

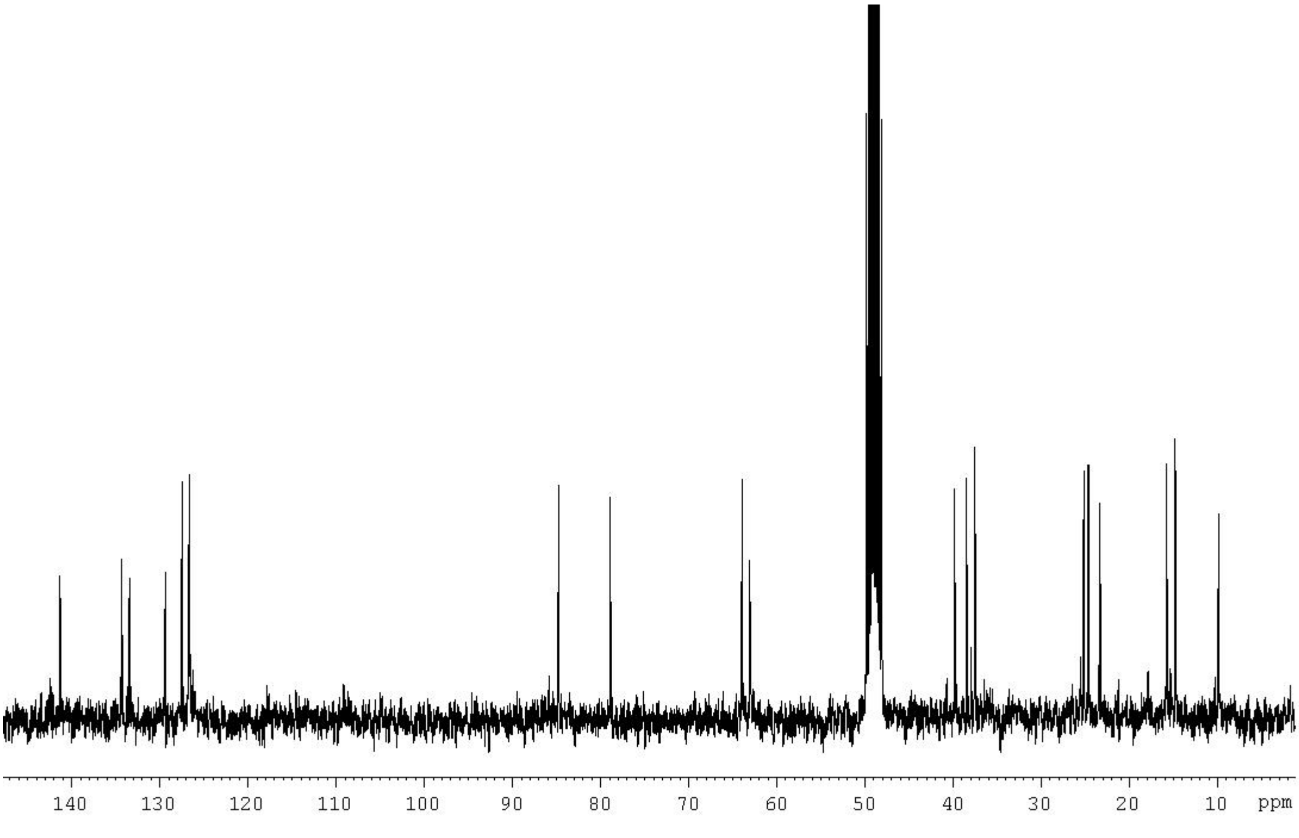

Figure S29. ^{13}C NMR spectrum of (2*S*)-isosarcophytoxide (**10**) in MeOH-d_4

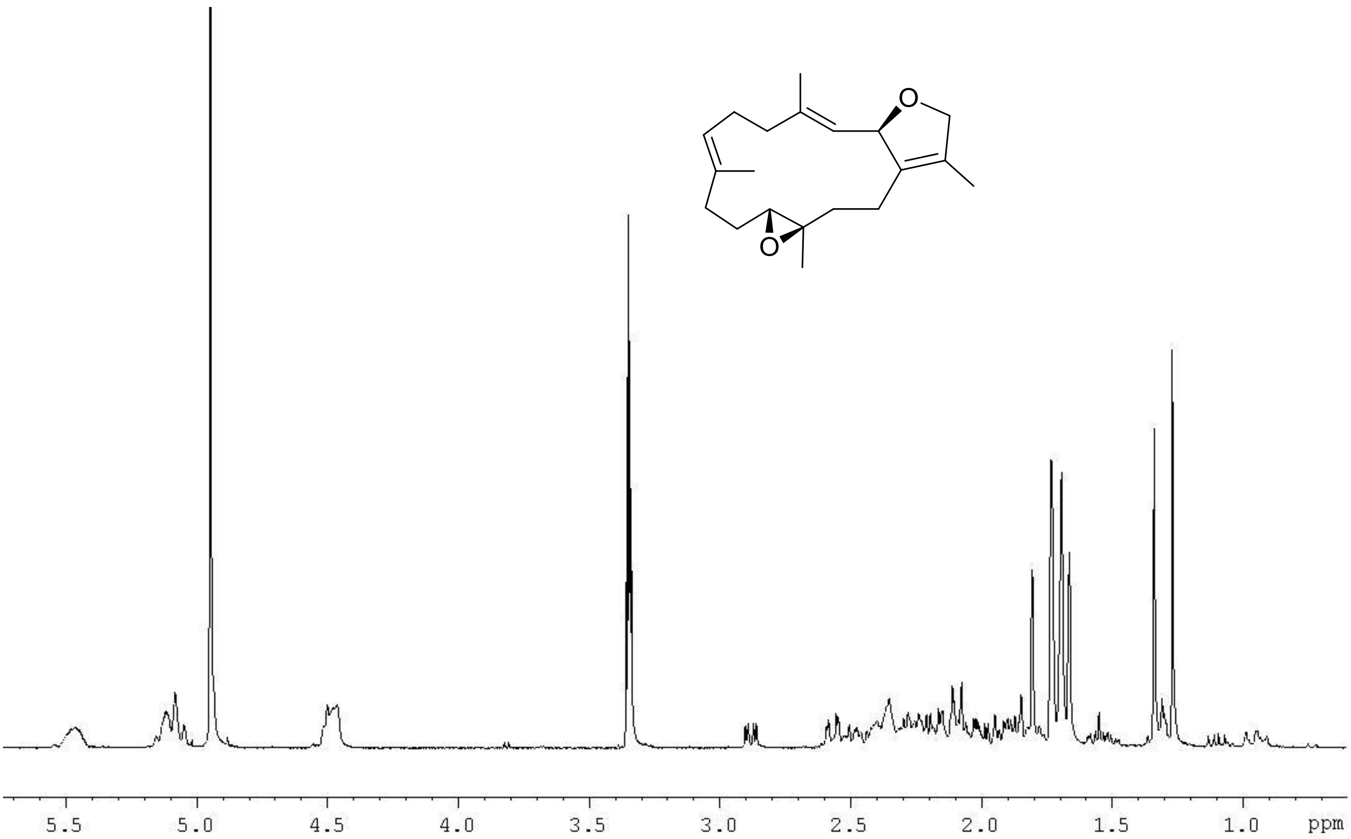

Figure S30. ^{1}H NMR spectrum of (2*R*)-isosarcophytoxide (**11**) in MeOH-d_4

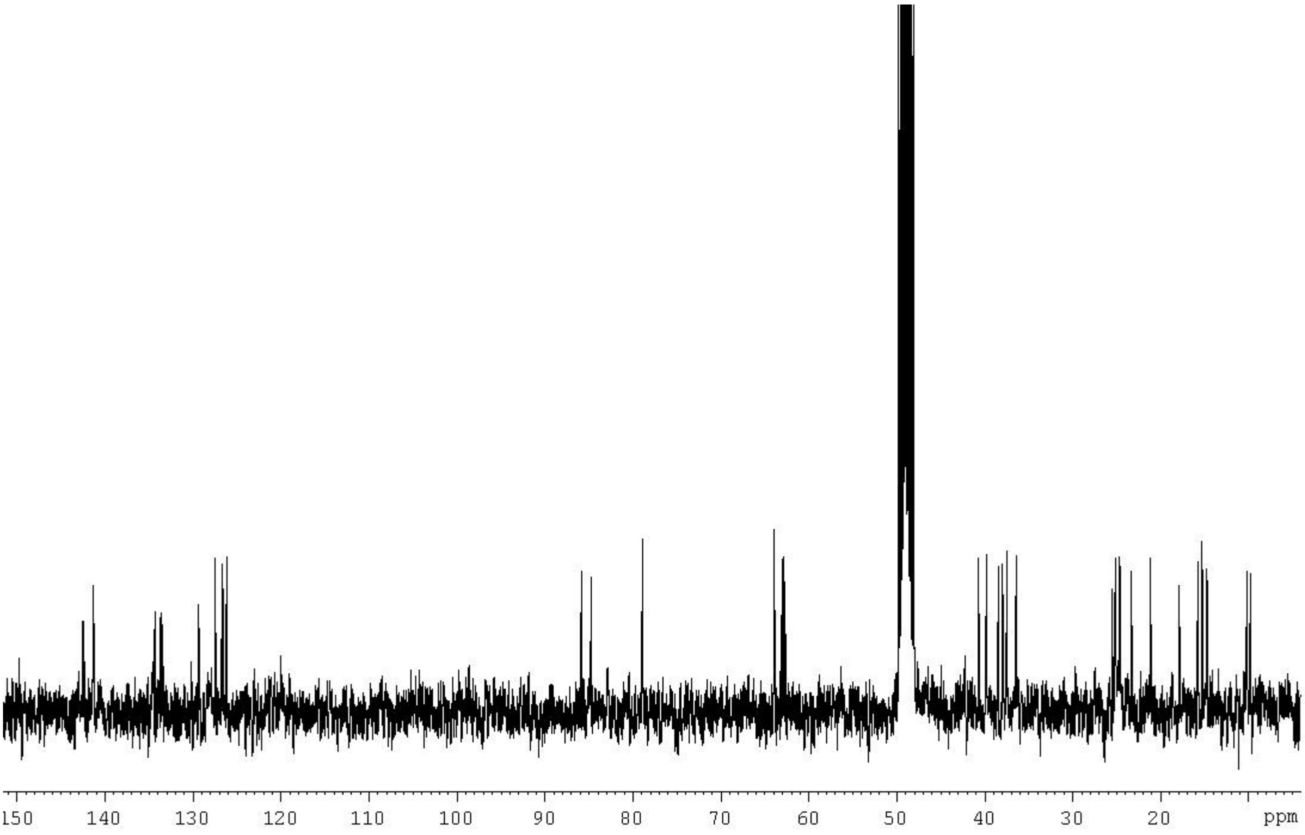

Figure S31. ^{13}C NMR spectrum of (2*R*)-isosarcophytoxide (**11**) in MeOH-d_4

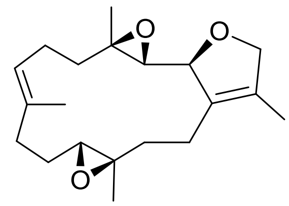

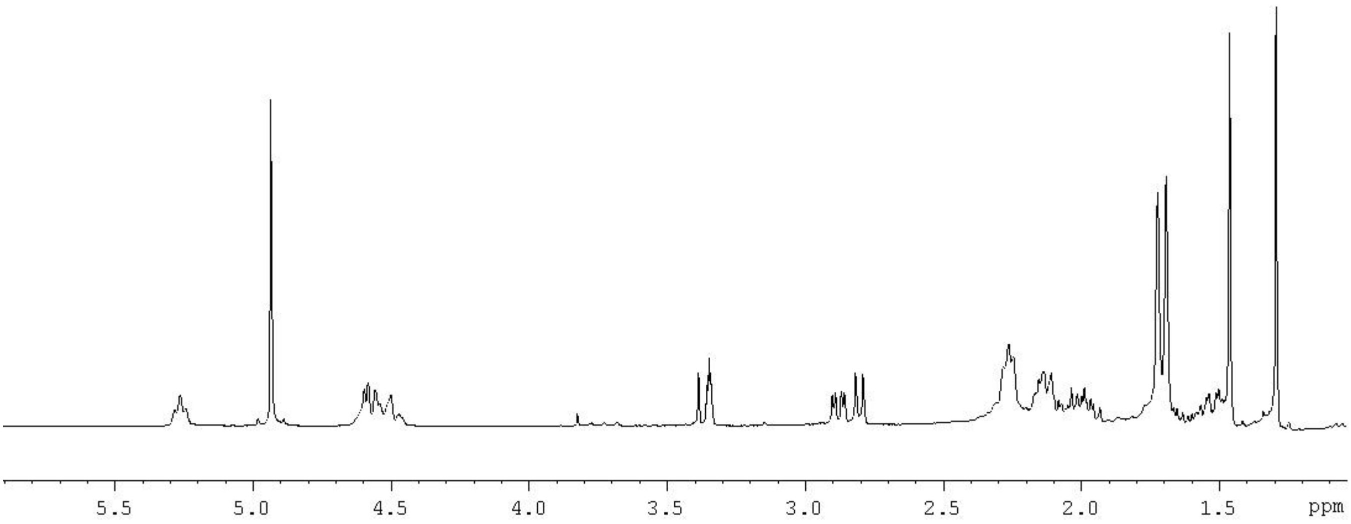

Figure S32. ^{1}H NMR spectrum of 2*S*,3*R*,4*R*,11*R*,12*R*-isosarcophytobisepoxide (**12**) in MeOH-d_4

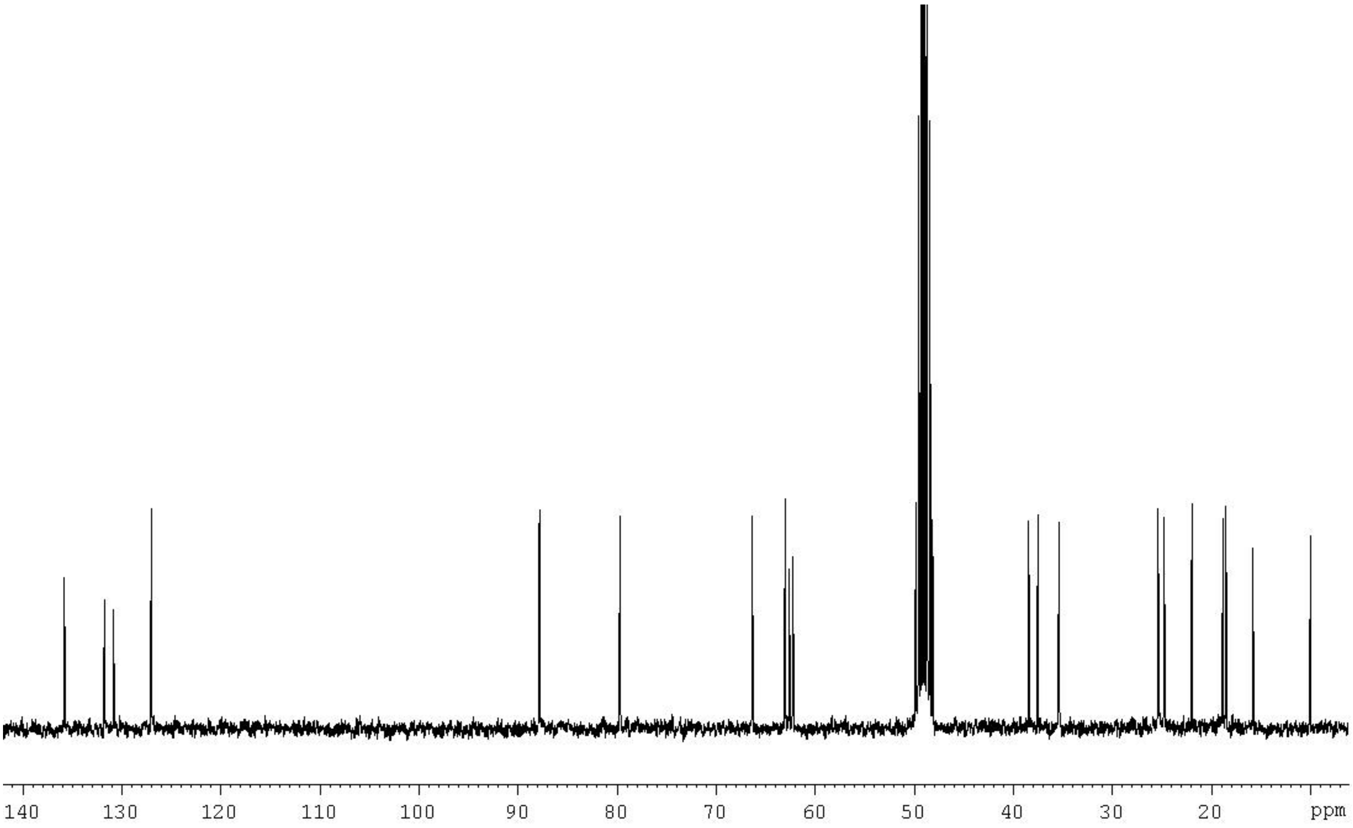

Figure S33. ^{13}C NMR spectrum of 2*S*,3*R*,4*R*,11*R*,12*R*-isosarcophytobisepoxide (**12**) in MeOH-d_4

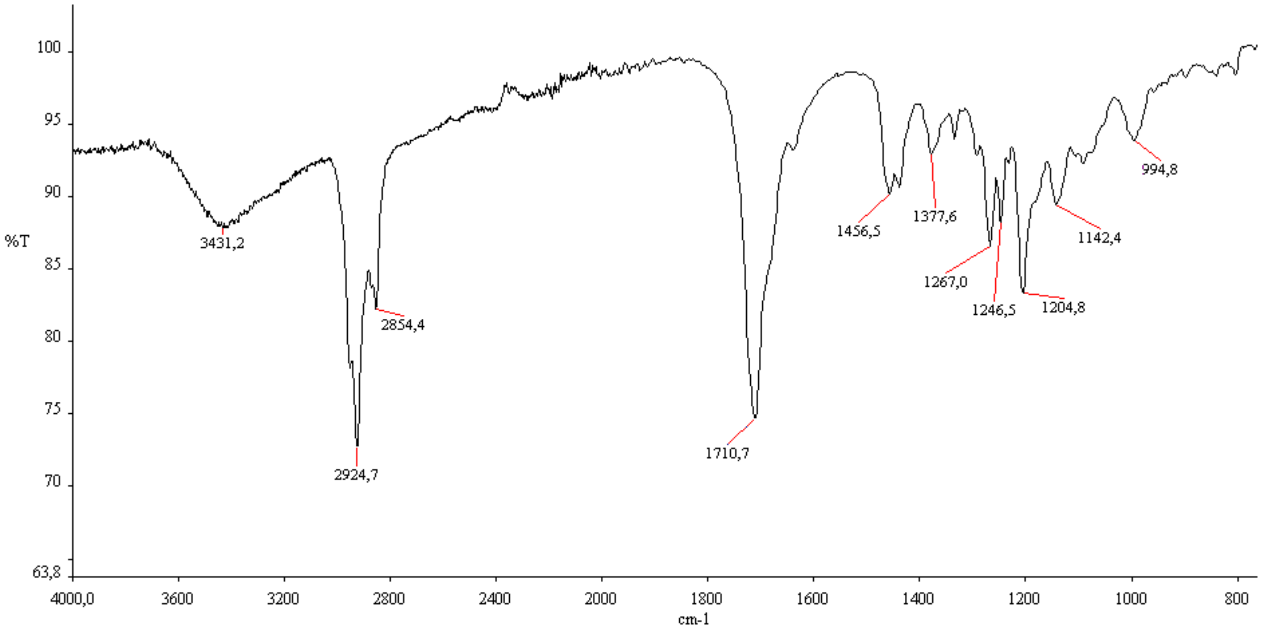

Figure S34. IR spectrum of 2*S*,3*R*,4*R*,11*R*,12*R*-isosarcophytobisepoxide (**12**)

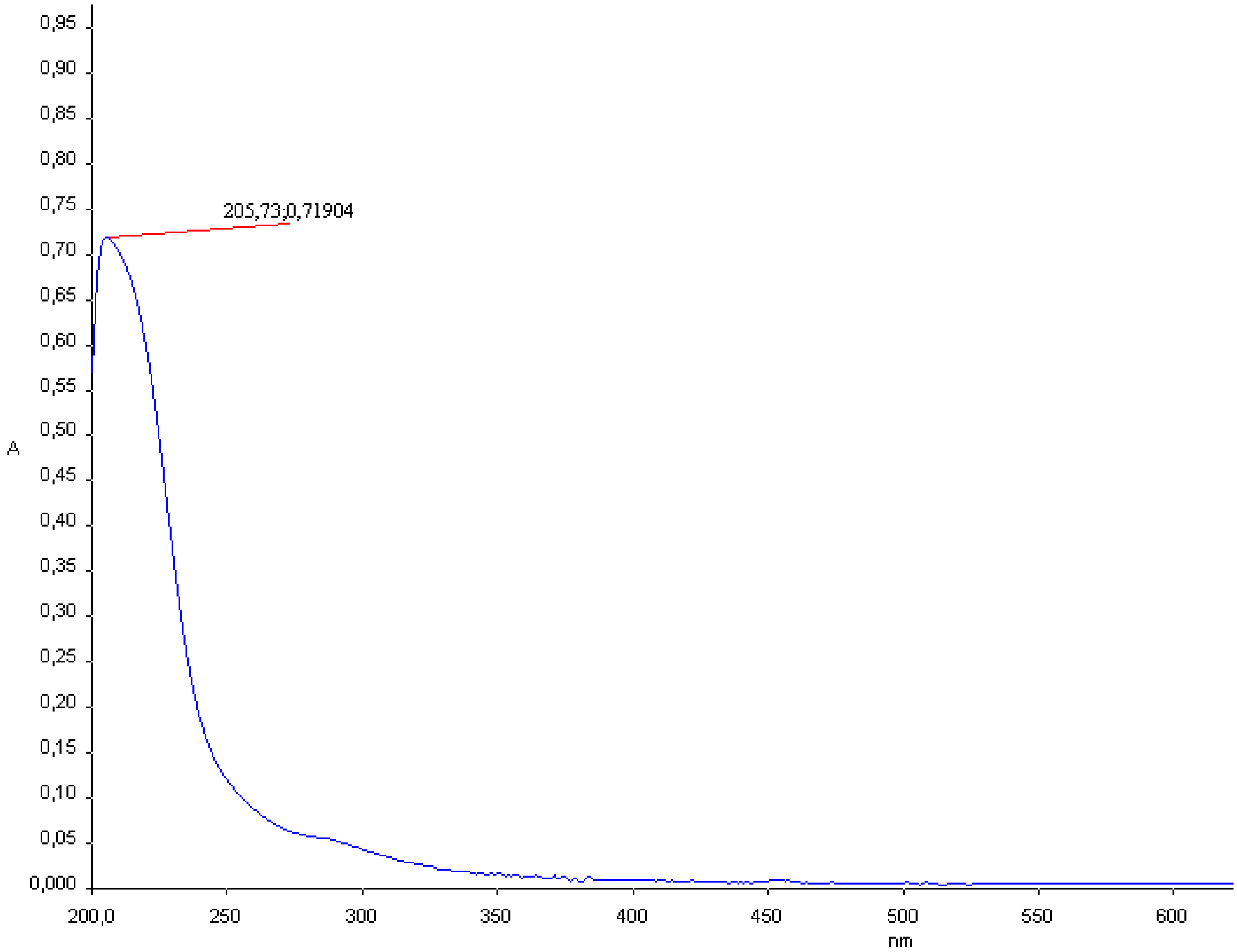

Figure S35. UV spectrum of 2*S*,3*R*,4*R*,11*R*,12*R*-isosarcophytobisepoxide (**12**) in MeOH

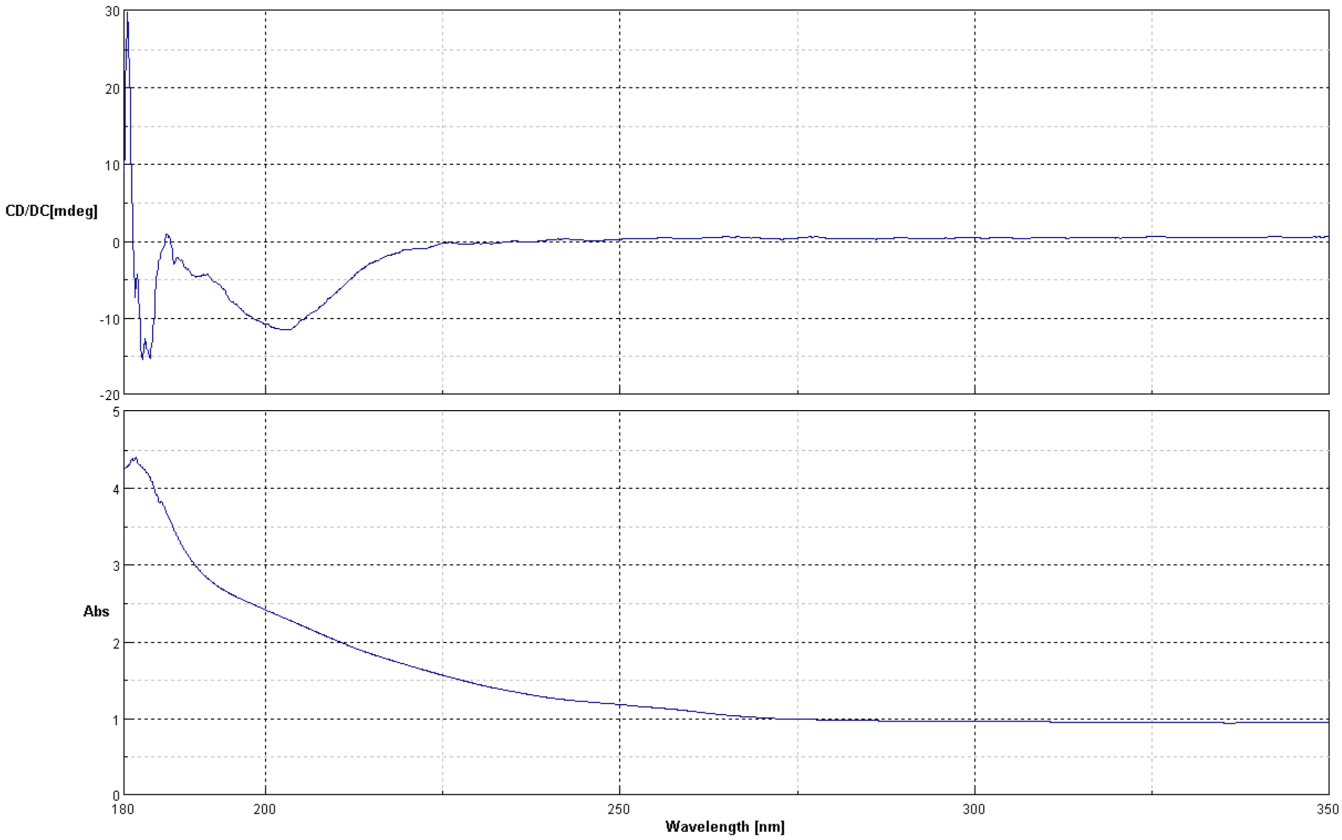

Figure S36. ECD spectrum of 2*S*,3*R*,4*R*,11*R*,12*R*-isosarcophytobisepoxide (**12**) in acetonitrile (*c* 0.01 mg/ml)

Table S4. NMR Data of 2*S*,3*R*,4*R*,11*R*,12*R*-isosarcophytobisepoxide (**12**) in MeOH-d_4

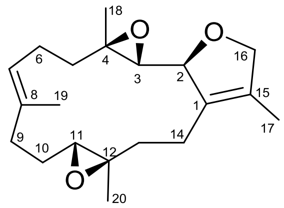

C [a]	δ_H (mult, *J* in Hz)	δ_C [b]	COSY	HMBC	NOESY
1		130.9, C			
2	4.59, d (8.0)	87.9, CH			H-3, H_3-18,
3	2.81, d (8.0)	66.4, CH	H-5	C-2, C-4, C-5, C-18	H-2, H-7, H-16a, H_3-18
4		62.3, C			
5	a 2.12, m<br>b 1.49, m	38.4, CH_2	H-5b, H_2-6<br>H-5a, H_2-6	C-18<br>C-18	
6	2.27, m	24.8, CH_2	H_2-5, H-7	C-4, C-5, C-7, C-8	
7	5.26, t (6.2)	127.1, CH	H_2-6, H_3-19		H-3, H_2-6, H_2-9, H_3-18
8		135.8, C			
9	2.26, m	37.5, CH_2	H_2-6		
10	2.03, m<br>1.56, m	25.4, CH_2	H_2-9, H-10b, H-11<br>H_2-9, H-10a, H-11		
11	2.88, dd (3.3, 9.9)	63.1, CH	H_2-10		H-7, H_3-19, H_3-20
12		62.6, C			
13	a 1.98, m<br>b 1.69, m	35.4, CH_2	H-13a, H_2-14<br>H-13b, H_2-14	C-1, C-14	
14	2.13, m	22.0, CH_2	H_2-13	C-1, C-2, C-13, C-15	
15		131.8, C			
16	a 4.56, m<br>b 4.50, m	79.7, CH_2	H-15, H-16b<br>H-15, H-16a	C-1, C-3, C-15<br>C-1, C-3, C-15	H-2<br>H_3-17
17	1.72, br s	10.0, CH_3	H-15	C-1, C-15, C-16	
18	1.46, s	18.5, CH_3		C-3, C-4, C-5	
19	1.69, br s	15.8, CH_3		C-7, C-8, C-9	
20	1.30, s	18.8, CH_3	H-11	C-11, C-12, C-13	H_2-10, H-11(w), H_2-13, H_2-14

[a] All assignments are based on extensive 1D and 2D NMR measurements (COSY, HSQC, HMBC).
[b] Multiplicities determined by DEPT.

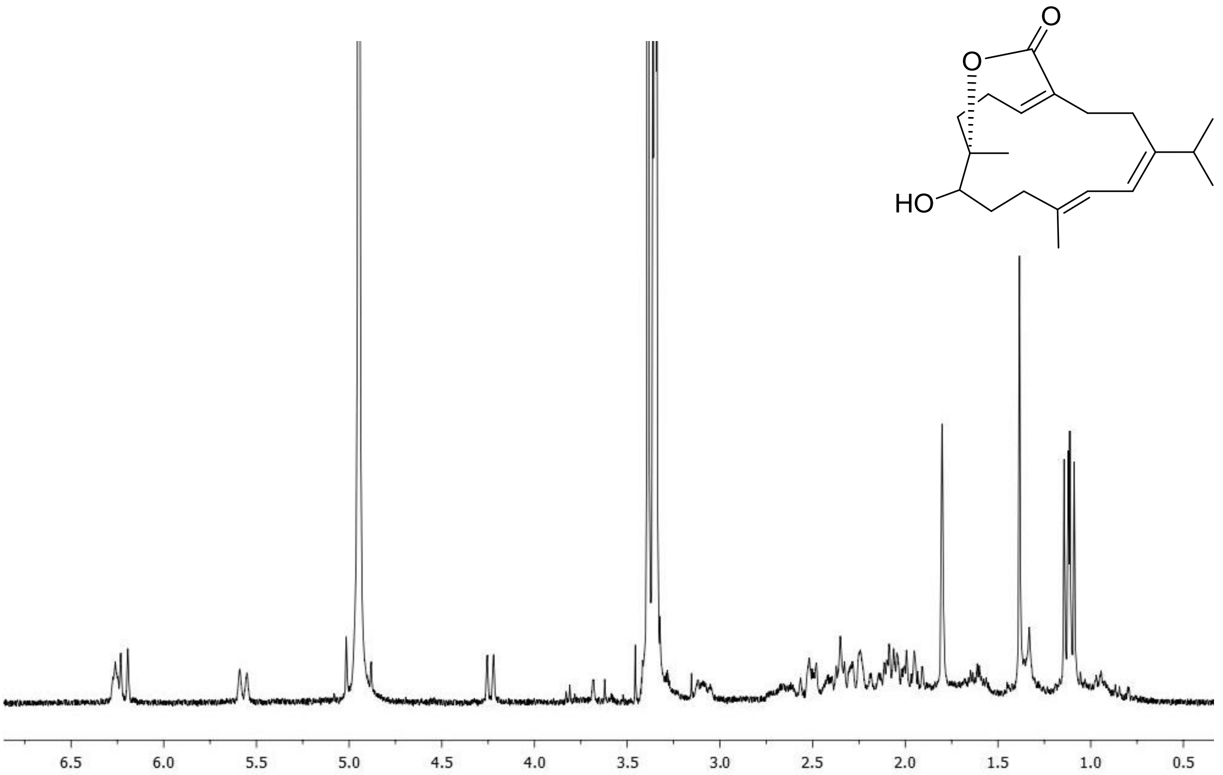

Figure S37. ^{1}H NMR spectrum of cembranoid (**13**) in MeOH-d_4

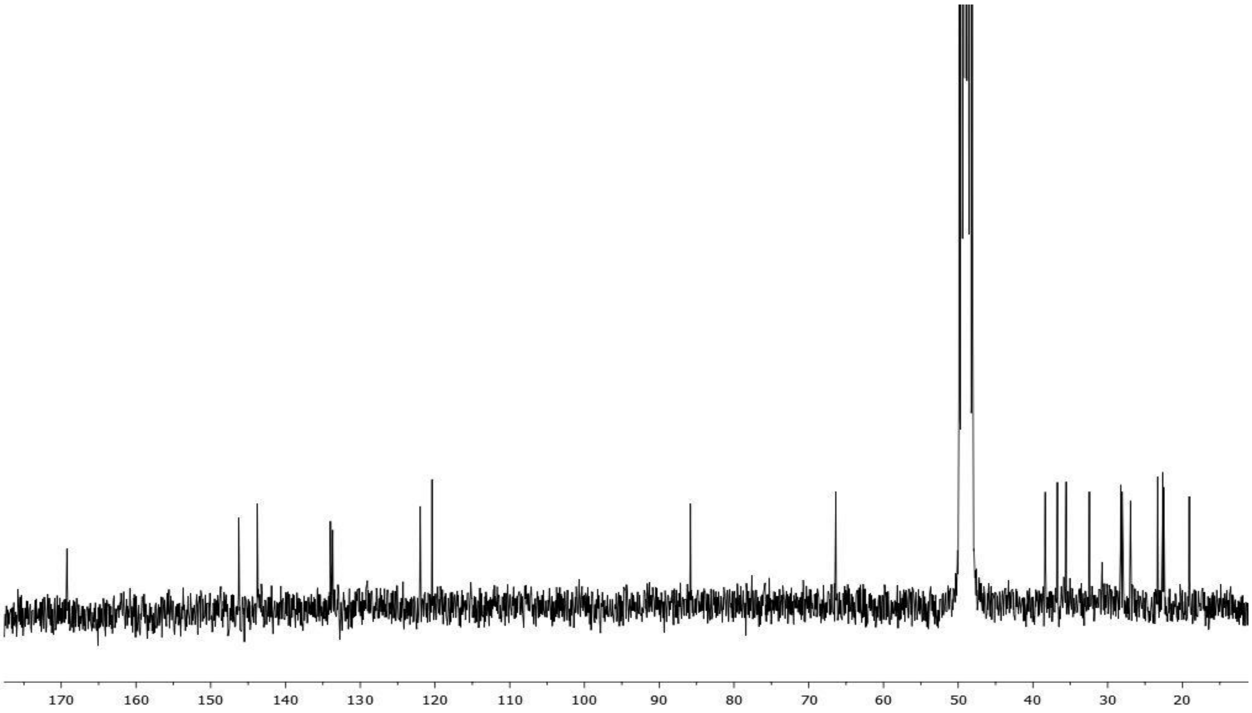

Figure S38. ^{13}C NMR spectrum of cembranoid (**13**) in MeOH-d_4

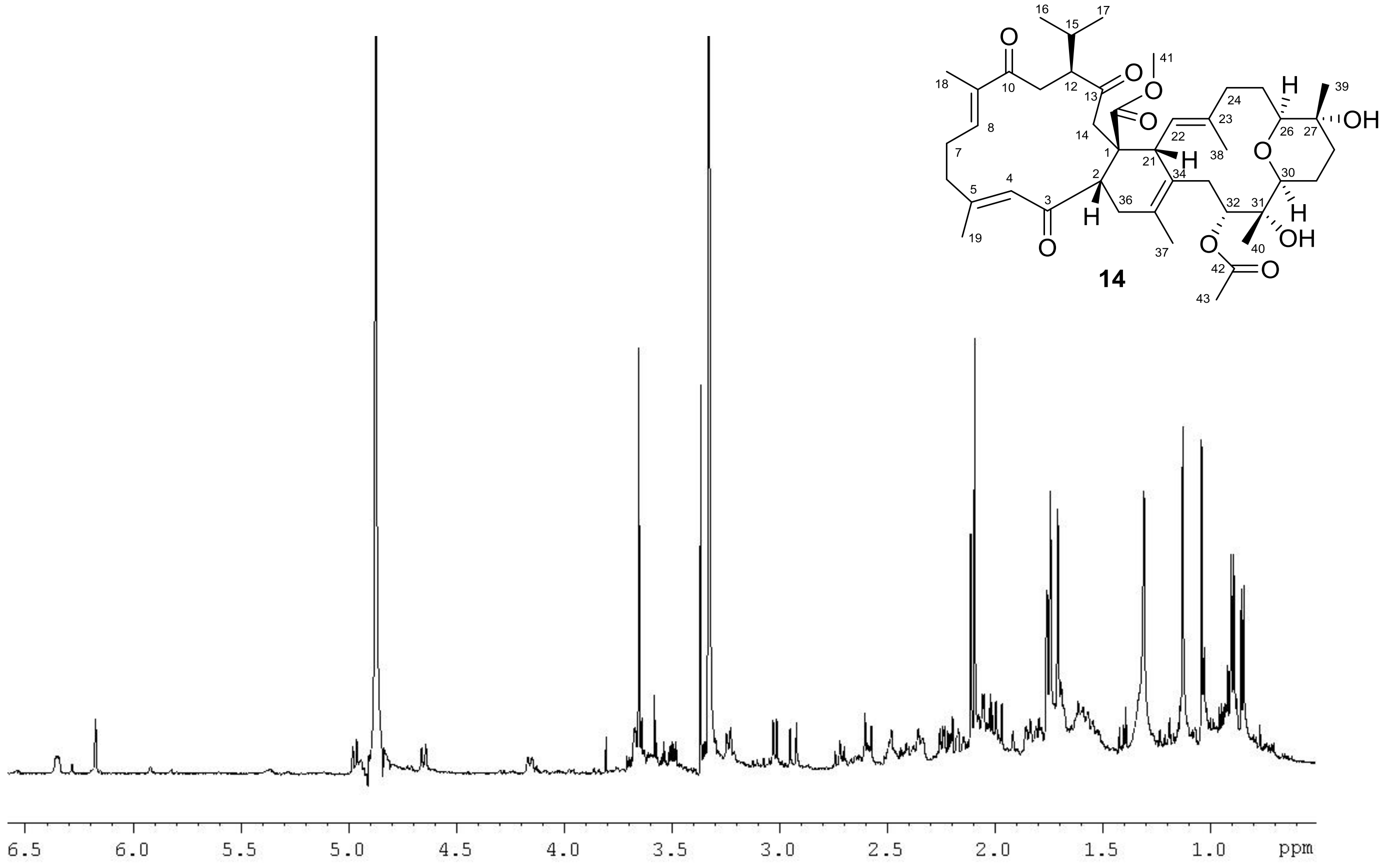

Figure S39. ^{1}H NMR spectrum of isobisglaucumlide B (**14**) in MeOH-d_4

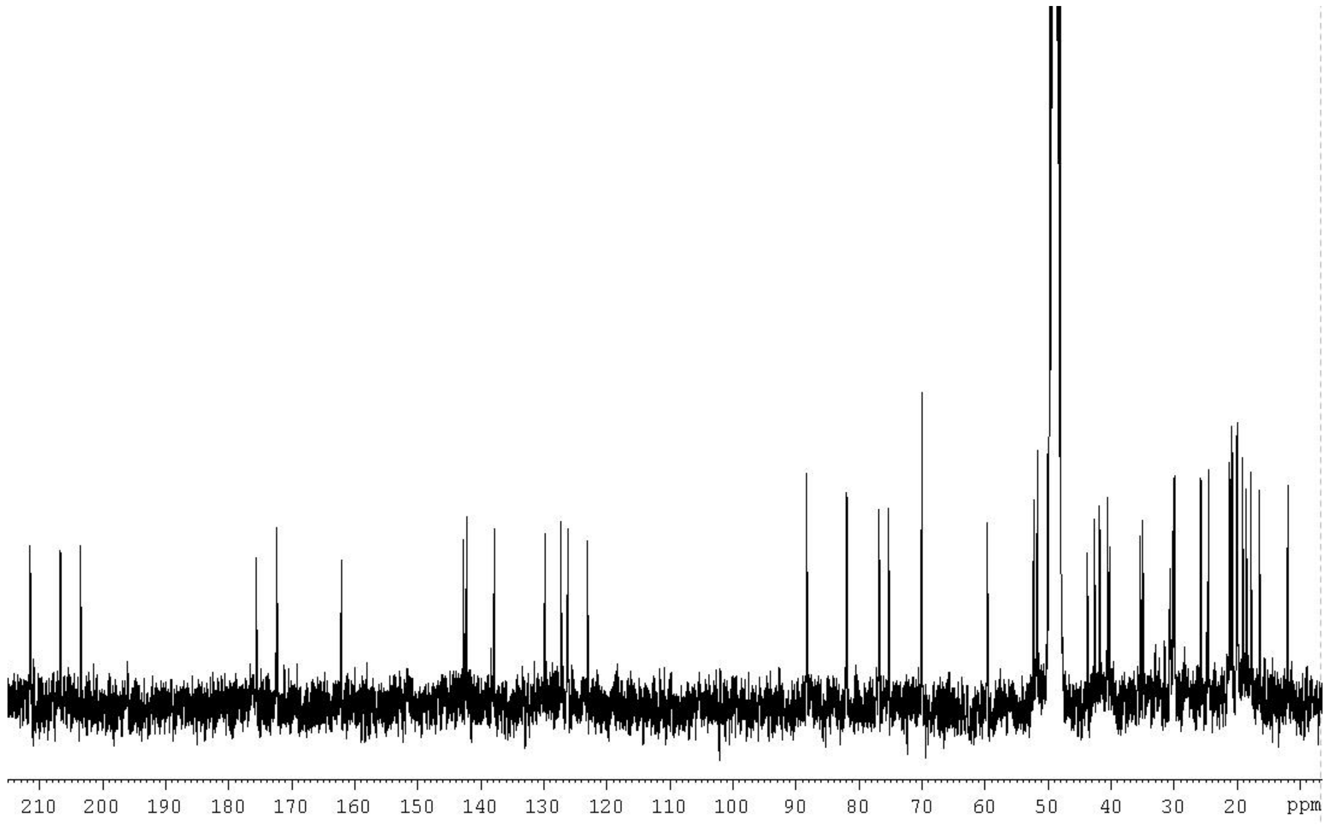

Figure S40. ^{13}C NMR spectrum of isobisglaucumlide B (**14**) in MeOH-d_4

Table S5. NMR data of isobisglaucumlide B (**14**) in MeOH-d_4

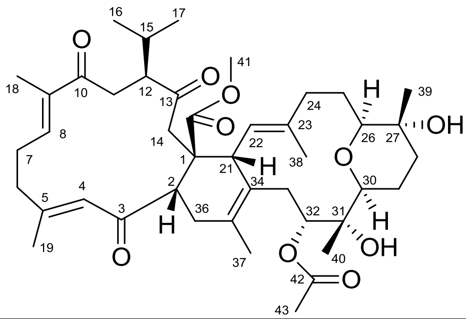

C[a]	δ_H (mult., J in Hz)	δ_C [b]	COSY	HMBC	ROESY
1		50.2, C			
2	3.50, m	52.4, CH	H-36 a	C-4	H-4, H-21, H_3-41
3		203.5, C			
4	6.18, s	126.3, CH	H_3-19	C-6	H-2, H_3-19
5		162.3, C			
6	a 2.48, m	40.3, CH_2		C-4, C-8, C-19	
	b 2.42, m			C-4, C-8, C-19	
7	a 2.64, m	25.9, CH_2	H-7b, H-8	C-5	H-6b, H_3-18
	b 2.49, m		H-7a, H-8	C-5	
8	6.36, br dd (7.0, 13.0)	142.4, CH	H_2-7, H_3-18		H-11a
9		138.0, C			
10		206.9, C			
11	a 3.35, dd (7.5, 20.0)	35.5, CH_2	H-11b, H-12	C-9, C-13, C-15	H-8, H-14 a
	b 2.24, m		H-11a, H-12	C-9, C-13, C-15	
12	2.60, br dd (6.9, 18.0)	59.7, CH	H_2-11, H-14a	C-10, C-15, C-16, C-17	
13		211.6, C			
14	a 2.94, d (18.0)	43.9, CH_2	H-12, H-14b	C-20, C-21, C-41	H-11a
	b 2.59, dd (7.0, 18.2)		H-14a	C-2, C-15, C-20, C-21,	
15	2.26, m	30.0, CH	H-11a, H-12, H_3-16, H_3-17	C-11, C-16, C-17	
16	0.86, d (7.0)	18.0, CH_3		C-12, C-15, C-17	
17	0.90, d (7.0)	21.3, CH_3		C-12, C-15, C-16	H-12
18	1.74, s	12.1, CH_3		C-8, C-9, C-10	
19	2.12, s	18.6, CH_3		C-4, C-5, C-6	H-4
20		175.7, C			
21	4.16, d (11.4)	42.7, CH	H-22, H_3-37		H-2, H-32, H_3-38
22	4.65, d (11.4)	123.2, CH	H-21, H-24a, H_3-38	C-24, C-34, C-38	H-24b
23		142.9, C			
24	a 2.35, m	41.9, CH_2	H-24b	C-22	
	b 2.07, m		H-24a	C-22	
25	a 1.81, m	25.8, CH_2	H-24a, H-25b	C-27, C-30, C-39	
	b 1.62, m		H-25a	C-27, C-30, C-39	
26	3.02, d (9.9)	88.5, CH	H-25 b, H2-28b	C-24, C-30, C-39	H-28b, H-30
27		70.1, C			
28	a 1.85, m	40.7, CH_2	H-28b	C-30	H-29a
	b 1.60, m		H-26, H-28a	C-26, C-30	
29	a 1.72, m	24.6, CH_2	H-29b		
	b 1.55, m		H-29a, H-30		
30	3.24, dd (2.0, 13.9)	82.1, CH	H-29b	C-31	H-26
31		75.4, C			
32	4.97, dd (2.0, 13.9)	76.9, CH	H_2-33	C-34, C-42	H-21, H_3-40
33	a 2.72, m	30.2, CH_2	H-32, H-33b	C-21, C-31, C-35	
	b 2.16, m		H-32, H-33a	C-21, C-31, C-35	
34		127.4, C			
35		129.9, C			
36	a 2.37, m	35.1, CH_2	H-2	C-1, C-34, C-37	
	b 2.21, m		H-2	C-34	
37	1.76, s	20.3, CH_3		C-34, C-35, C-36	H-33a, H_2-36
38	1.71, s	16.6, CH_3		C-22, C-23, C-24	H-21
39	1.13, s	20.0, CH_3		C-25, C-26, C-27, C-28	H-28b
40	1.04, s	19.3, CH_3		C-30, C-31, C-32	H-29b
41	3.65, s	51.8, CH_3		C-20	H-4, H-2, H-14b
42		172.5, C			
43	2.10, s	20.9, CH_3		C-32, C-42	

[a] All assignments are based on extensive 1D and 2D NMR measurements (COSY, HSQC, HMBC, ROESY).
[b] Multiplicities determined by DEPT.

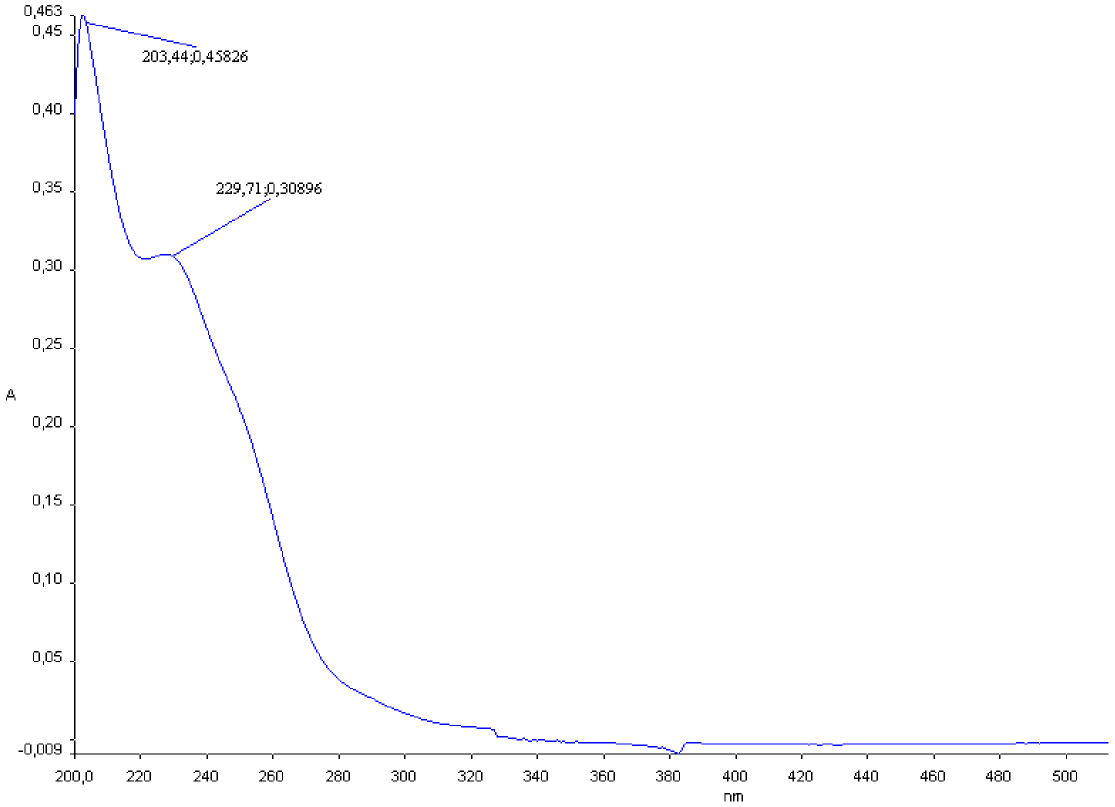

Figure S41. UV spectrum of isobisglaucumlide B (**14**) in MeOH

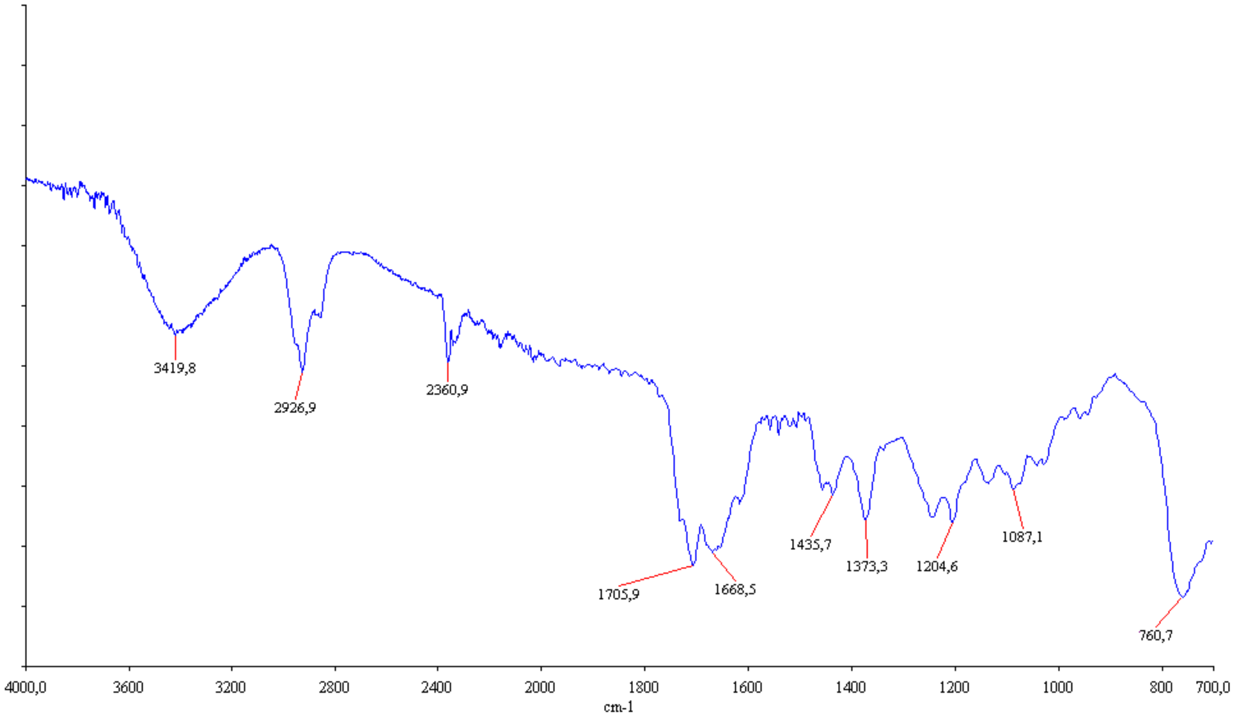

Figure S42. IR spectrum of isobisglaucumlide B (**14**)

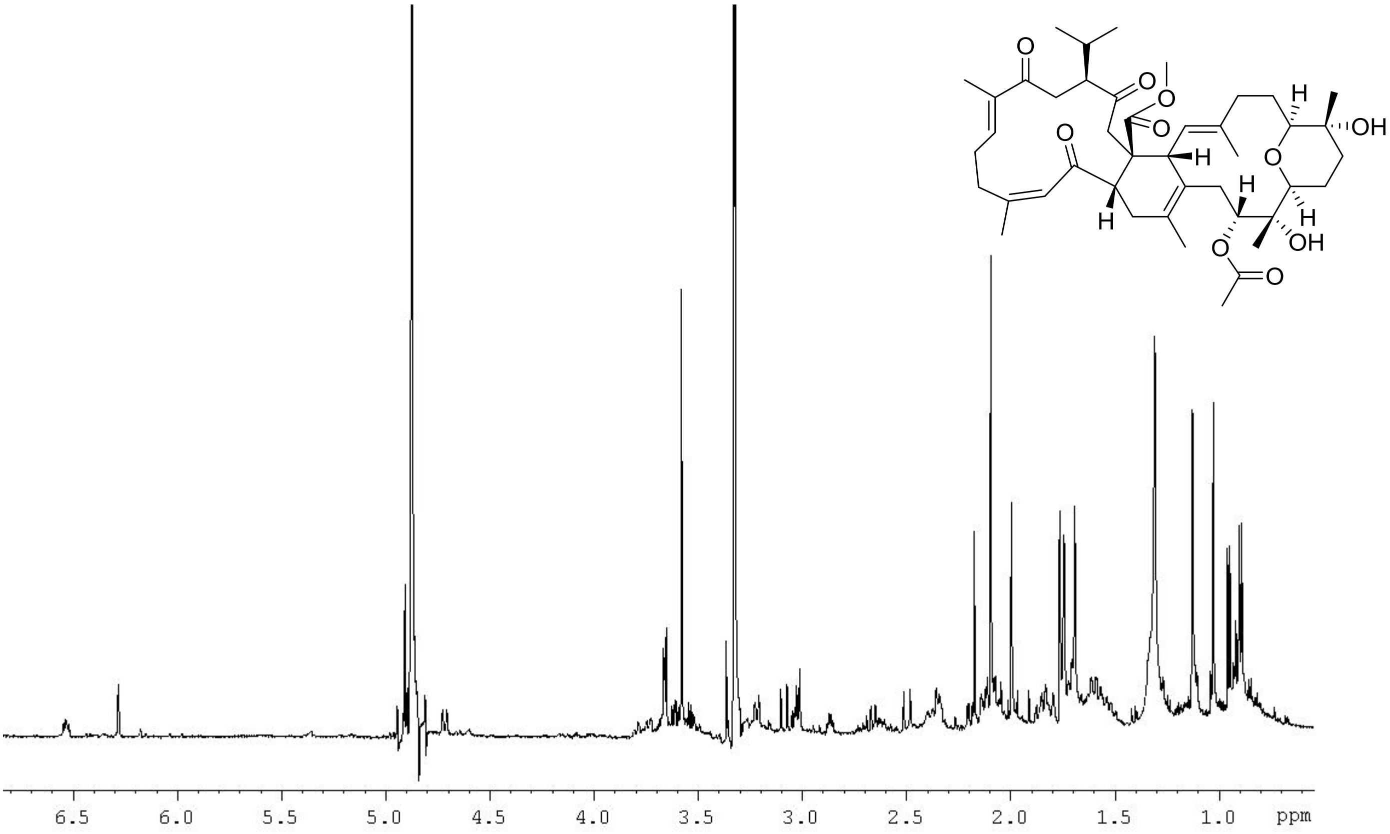

Figure S43. ^{1}H NMR spectrum of isobisglaucumlide C (**15**) in MeOH-d_4

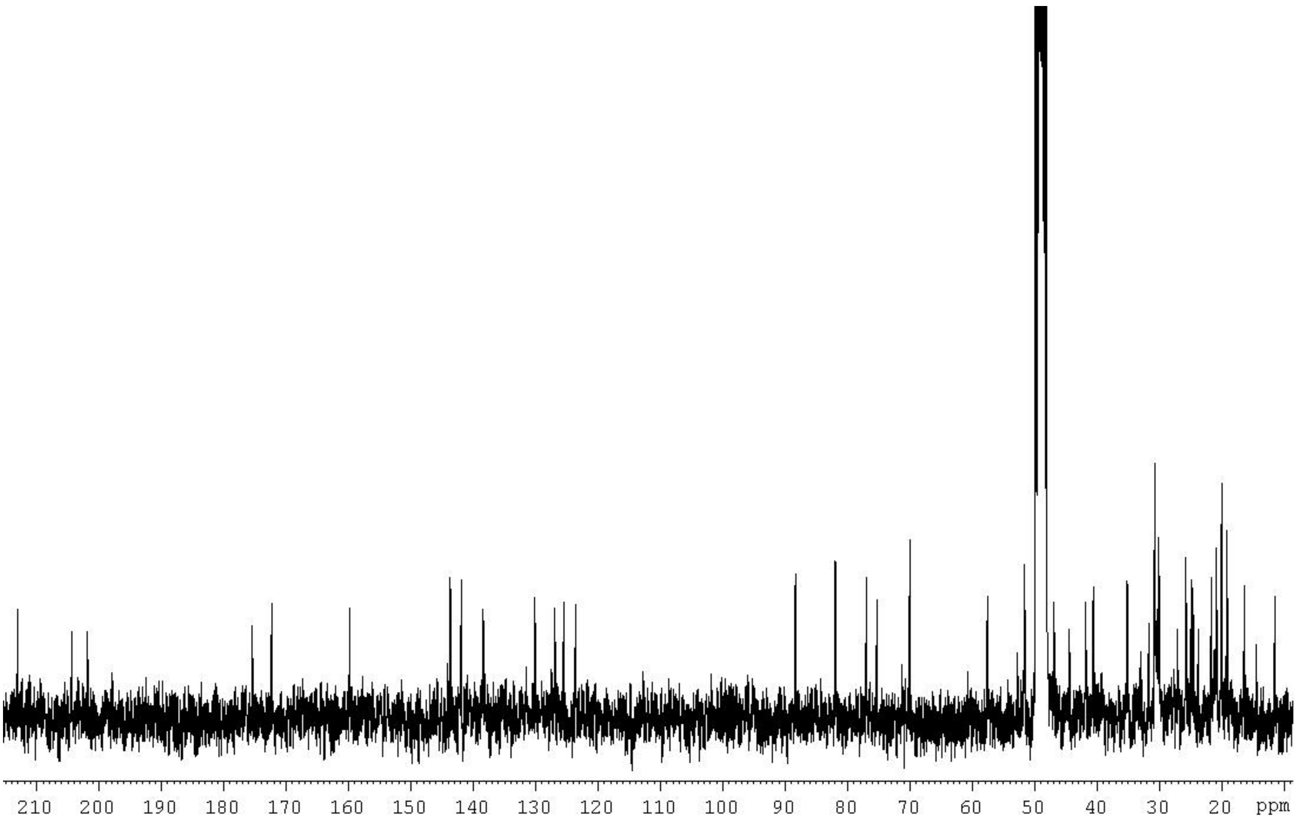

Figure S44. ^{13}C NMR spectrum of isobisglaucumlide C (**15**) in MeOH-d_4

Table S6. NMR data of isobisglaucumlide C (**15**) in MeOH-d_4.

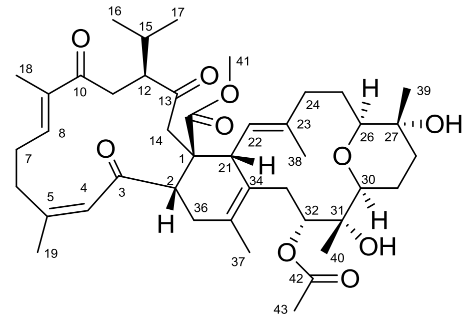

C[a]	δ_H (mult., J in Hz)	δ_C [b]	COSY	HMBC	ROESY
1		49.5, C			
2	3.62, dd (8.1, 10.8)	52.8, CH	H_2-36	C-1, C-3, C-14 (w), C-20, C-36	H-4, H-21, H_3-41
3		202.0, C			
4	6.29, s	125.6, CH	H_3-19	C-3, C-5, C-6, C-19	H-2, H_3-19
5		159.9, C			
6	a 3.79, m	31.7, CH_2	H-6b	C-4, C-5	
	b 2.14, m		H-6a, H-7a	C-4, C-5	
7	a 2.65, m	27.1, CH_2	H-7b	C-6, C-8, C-9	H-6b, H_3-18
	b 2.41, m		H-6a, H-7a, H-8	C-6, C-8, C-9	
8	6.54, m	143.8, CH	H-7a		H-11a
9		138.5, C			
10		204.5, C			
11	a 3.04, m	35.3, CH_2	H-11b, H-12	C-10, C-12, C-13, C-15	H-8, H-14 a
	b 2.19, m		H-11a, H-12	C-10, C-12, C-13, C-15, C-17	
12	2.87, dd (6.4, 10.5)	57.6, CH	H_2-11	C-10, C-11, C-13, C-15, C-16, C-17	
13		213.0, C			
14	a 3.09, br d (18.0)	46.9, CH_2	H-14b	C-1, C-2, C-13, C-20, C-21, C-34 (w)	H-11a
	b 2.50, br d (18.0)		H-14a	C-1, C-2, C-13, C-20, C-21	
15	2.13, m	30.2, CH			
16	0.90, d (6.8)	19.2, CH_3	H-15	C-12, C-15, C-17	
17	0.96, d (6.8)	21.7, CH_3	H-15	C-12, C-15, C-16	H-12
18	1.78, s	11.5, CH_3	H-8	C-8, C-9, C-10,	
19	2.00, s	24.9, CH_3	H-4	C-3 (w), C-4, C-5, C-6	H-4
20		175.5, C			
21	3.75, m	44.5, CH	H-22, H_3-37 (w)		H-2, H_3-38
22	4.72, d (11.7)	123.7, CH	H-21, H_3-38	C-24, C-38	H-24b
23		142.0, C			
24	a 2.39, m	41.8, CH_2	H-24b, H-25a		
	b 2.09, m		H-24a, H-25b	C-22 (w), C-23, C-26 (w), C-38 (w)	
25	a 1.81, m	25.8, CH_2	H-24a, H-25b	C-23, C-24, C-26, C-27, C-30	
	b 1.61, m		H-25a, H-26		
26	3.03, m	88.4, CH	H-25b, H-28b	C-24, C-25, C-27, C-28, C-30, C-39	H-28b, H-30
27		70.1, C			
28	a 1.85, m	40.6, CH_2	H-28b, H_2-29	C-26, C-27, C-30, C-39	H-29a
	b 1.60, m		H-26, H-28a	C-27, C-29	
29	a 1.72, m	24.6, CH_2	H-29b		
	b 1.54, m		H-28a, H-29a, H-30		
30	3.23, m	82.0, CH	H-29b, H-33a (w), H_3-38	C-26 (w), C-28 (w), C-31, C-40	H-26
31		75.3, C			
32	4.91, m	77.0, CH	H_2-33	C-33 (w), C-34, C-40 (w), C-42	H-21, H_3-40
33	a 2.69, m	30.0, CH_2	H-32, H-33b	C-21, C-31, C-32, C-34, C-35	
	b 2.25, m		H-33a	C-34	
34		127.0, C			
35		130.2, C			
36	2.36, m	35.1, CH_2	H-2		
37	1.75, s	20.2, CH_3	H-21	C-34, C-35, C-36	H-33a, H_2-36
38	1.70, s	16.4, CH_3	H-22	C-22, C-23, C-24	H-21
39	1.13, s	20.0, CH_3		C-26, C-27, C-28	H-28b
40	1.03, s	19.2, CH_3		C-30, C-31, C-32	H-29b
41	3.58, s	51.6, CH_3		C-20	H-4, H-2, H-14b
42		172.5, C			
43	2.10, s	20.9, CH_3		C-32, C-42	

[a] All assignments are based on extensive 1D and 2D NMR measurements (COSY, HSQC, HMBC, ROESY).
[b] Multiplicities determined by DEPT.

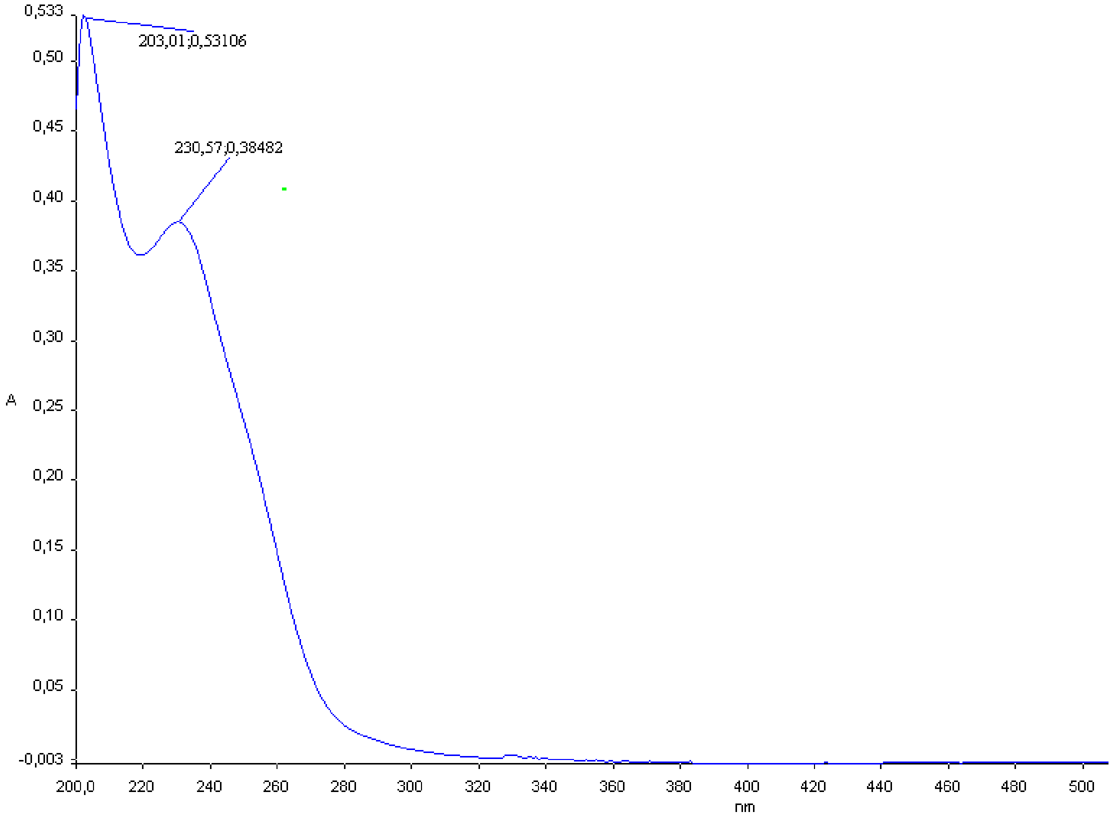

Figure S45. UV spectrum of isobisglaucumlide C (**15**) in MeOH

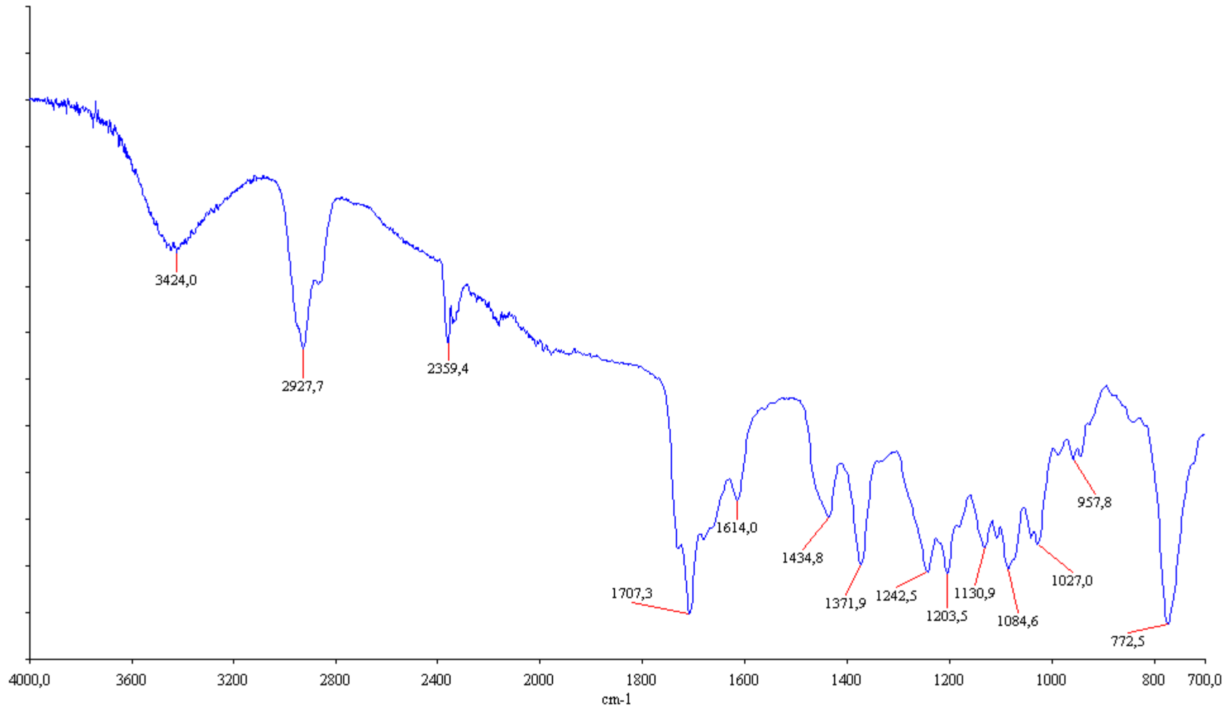

Figure S46. IR spectrum of isobisglaucumlide C (**15**)

Table S7A. Secondary metabolites of *P. longicirrum* isolated and identified in the current investigation in correlation with UPLC-HRMS data

**Molecular weight and *m/z* of prominent ion peaks**	**Chemical structure of putative metabolite**	**Presence in Fraction/ RT in min**
M+: 492<br>(M+H: 493.39)	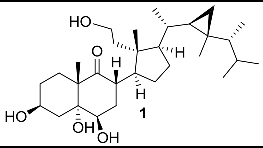 1 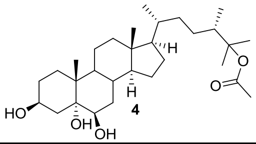 4	VLC7/ 14.7
M+: 450<br>(M+H: 451.32)	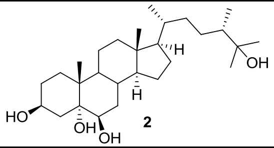 2	VLC7/ 15.0
M+: 448<br>(M+H: 449.36)	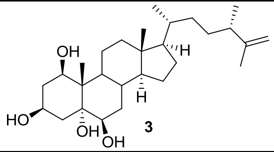 3	VLC7/ 14.2
M+: 332<br>(M+H: 333.23)	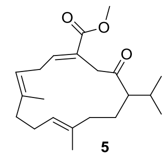 5	VLC8/ 14.2
M+: 300<br>(M+H: 301.22,<br>M+Na: 323.20)	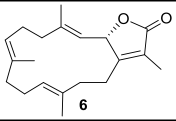 6	VLC7/ 17.0<br>VLC8/ 17.0
M+: 344<br>(M+H: 345.21,<br>M+Na: 367.19)	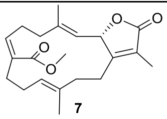 7	VLC6/ 15.4<br>VLC7/ 15.4
M+: 316<br>(M+H: 317.21)	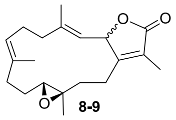 8-9	VLC6/ 12.0, 12.7<br>VLC7/ 12.70
M+: 302<br>(M+H: 303.23)	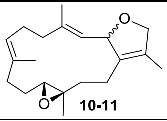 10-11	VLC7/ 16.1<br>VLC8/ 16.1
M+: 318<br>(M+H: 319.23,<br>M+Na: 341.21)	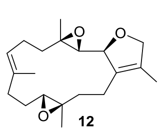 12 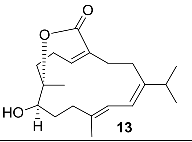 13	VLC3/ 12.2<br>VLC4/ 12.2<br>VLC5/ 14.1<br>VLC6/ 14.3<br>VLC7/ 14.1, 14.3
M+: 738<br>(M+H: 739.44,<br>M+Na: 761.42)	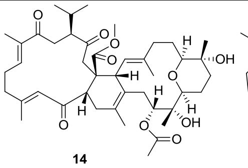 14 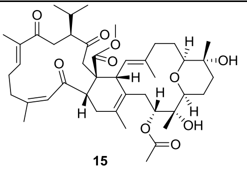 15	VLC6/ 14.3*, 14.5,14.9<br>VLC7 14.5, 14.9, 15.3*
M+: 362<br>(M+H: 363.22,<br>M+Na: 385.20)	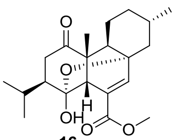 16 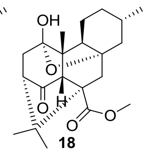 18	VLC3/ 13.2<br>VLC4/ 13.2<br>VLC5/ 13.0, 14.7<br>VLC6/ 13.0, 14.7, 16.3**<br>VLC7/ 13.0, 14.7, 16.3**

M+: 376<br>(M+H: 377.23)	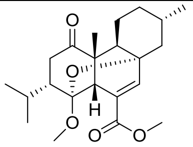<br>19	VLC6/ 15.1

Table S7B. Secondary metabolites reported from various *Sarcophyton* spp., not isolated in the current study of *P. longicirrum*, but probably present according to UPLC-HRMS data

**Molecular weight and *m/z* of ion peaks**	**Chemical structure**	**Presence in Fraction/ RT in min**
M+: 316<br>(M+H: 317.21)	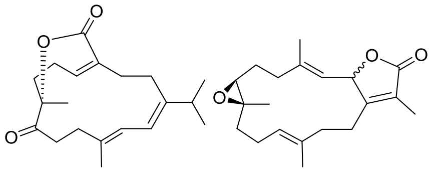	VLC6/ 14.0*<br>VLC7/, 15.9*
M+: 286<br>(M+H: 287.24)	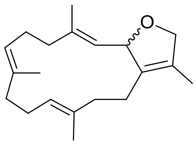	VLC7/ 15.0<br>VLC8/ 15.0
M+: 304<br>(M+H: 305.25)	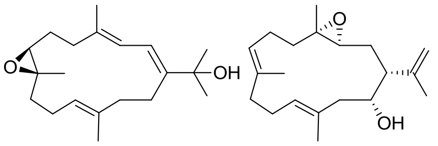	VLC5/ 13.5<br>VLC8/ 14.4
M+: 396<br>(M+H: 397.35)	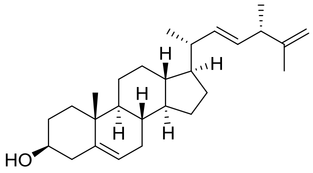	VLC7/ 13.9, 15.7*<br>VLC8/ 13.9, 15.7*
M+: 414<br>(M+H: 415.36<br>M+Na: 437.34)	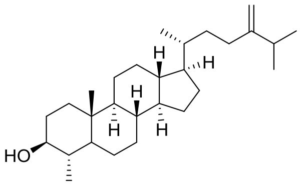 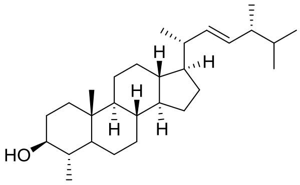	VLC7/ 13.9
M+: 442<br>(M+H: 443.35)	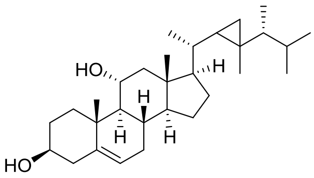	VLC7/ 14.9
M+: 466<br>(M+H: 467.37)	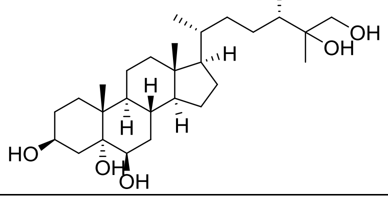	VLC7/ 14.2

M+: 486<br>(M+H: 487.36)	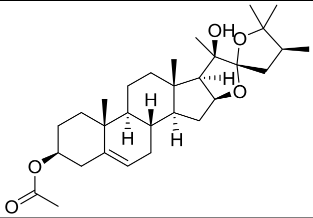	VLC7/ 17.7

Putative, not yet described biscembranoids containing **5** as dienophilic (western) part

M+: 710<br>(M+H: 711.39)	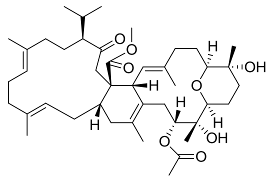	VLC6/ 15.4<br>VLC7/ 15.4
M+: 668<br>(M+H: 669.44)	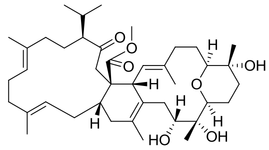	VLC7/ 12.4

* The presence of further isomers is very likely
** 4-Oxochatancin occurs in equilibrium of three mesomeric forms

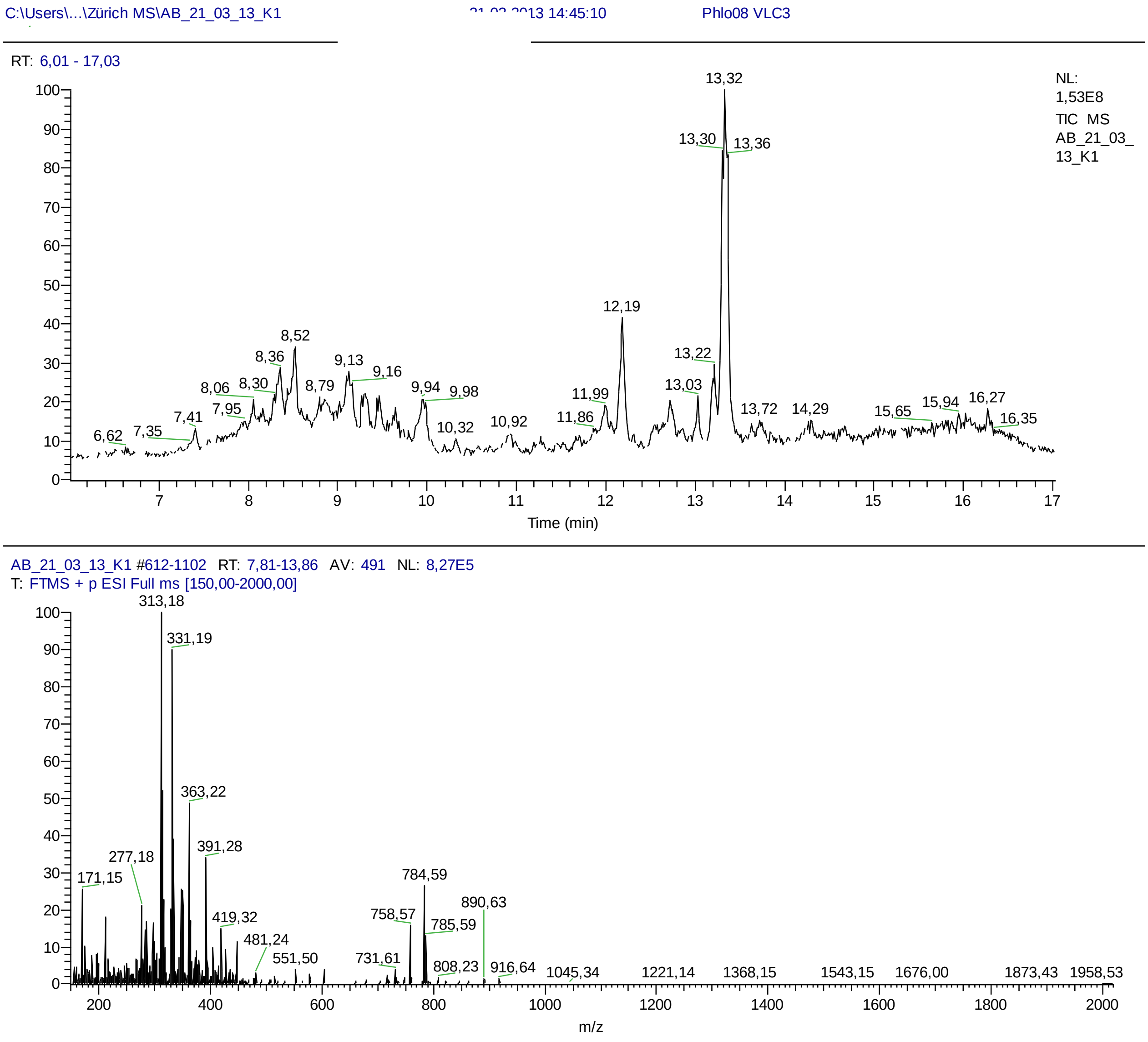

Figure S47. UPLC-HRMS chromatogram of VLC3

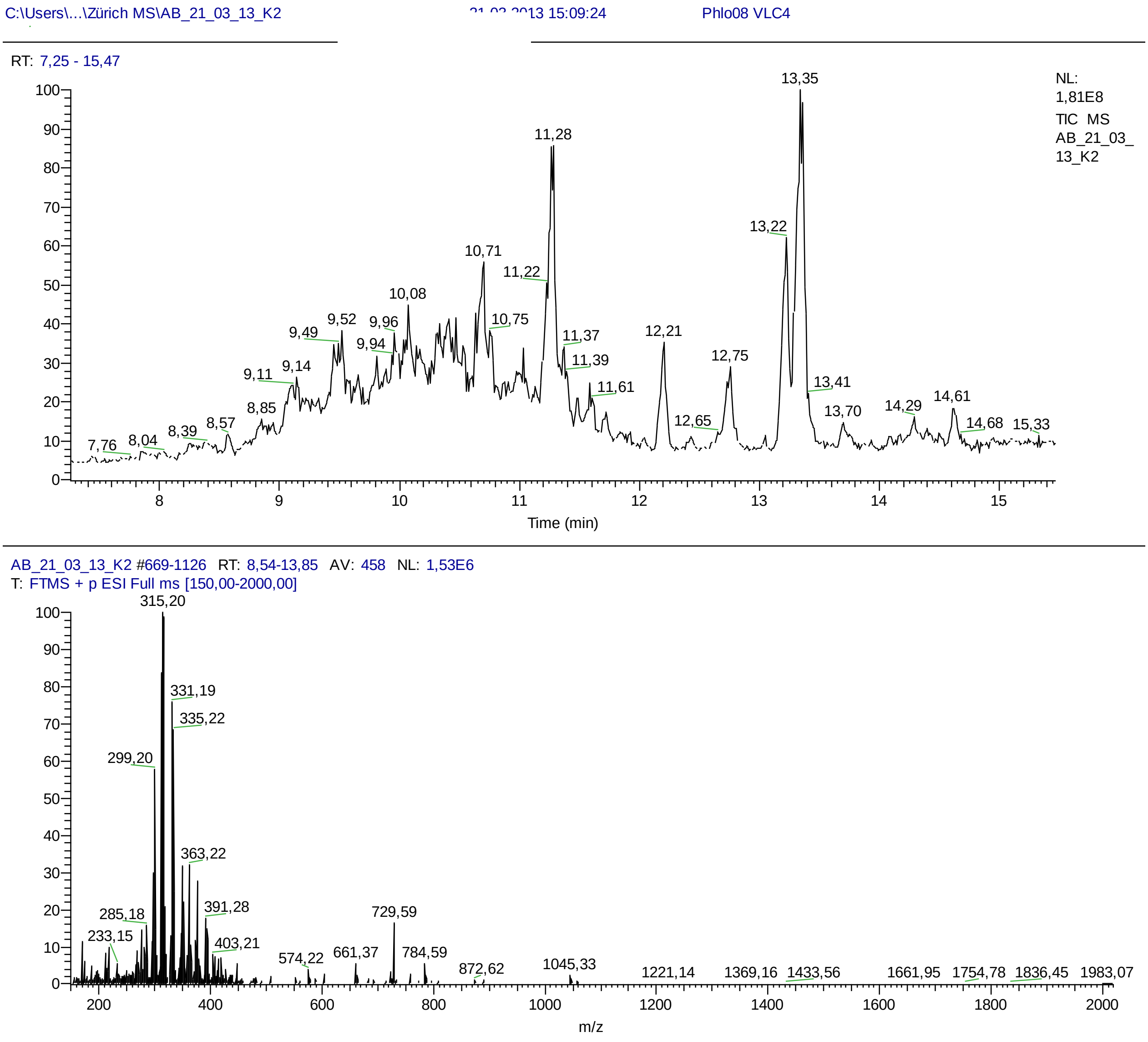

Figure S48. UPLC-HRMS chromatogram of VLC4

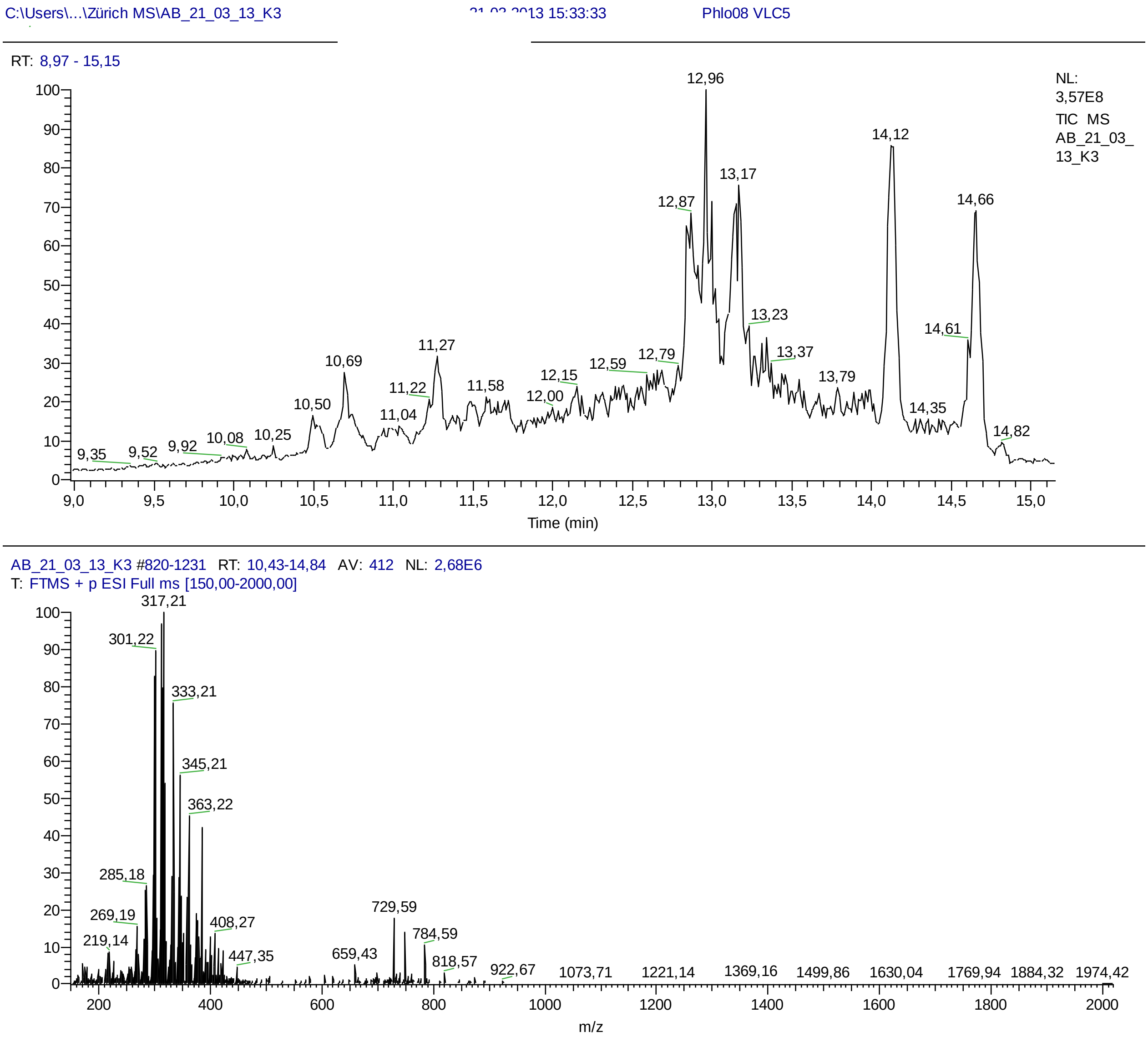

Figure S49. UPLC-HRMS chromatogram of VLC5

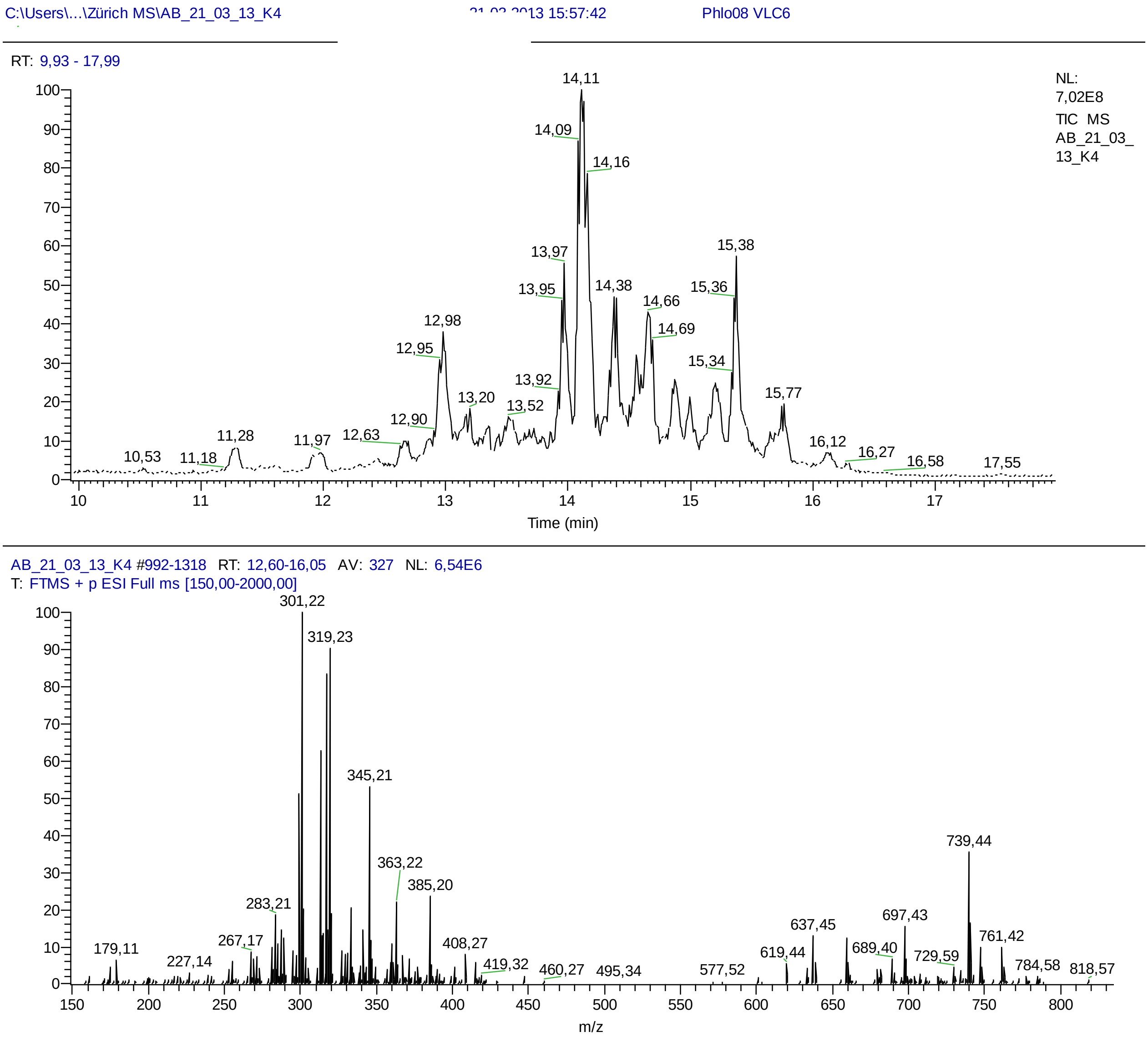

Figure S50. UPLC-HRMS chromatogram of VLC6

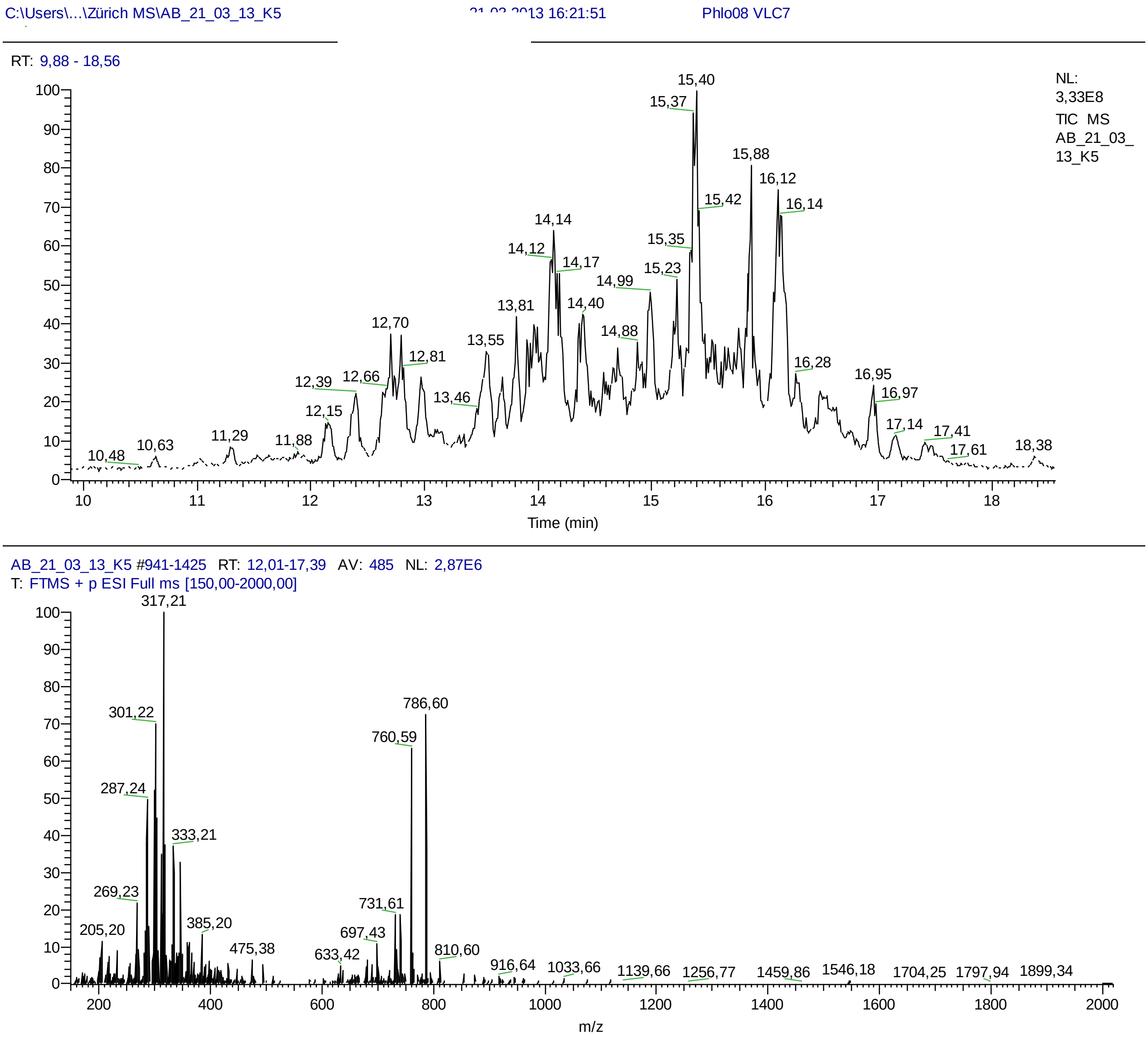

Figure S51. UPLC-HRMS chromatogram of VLC7

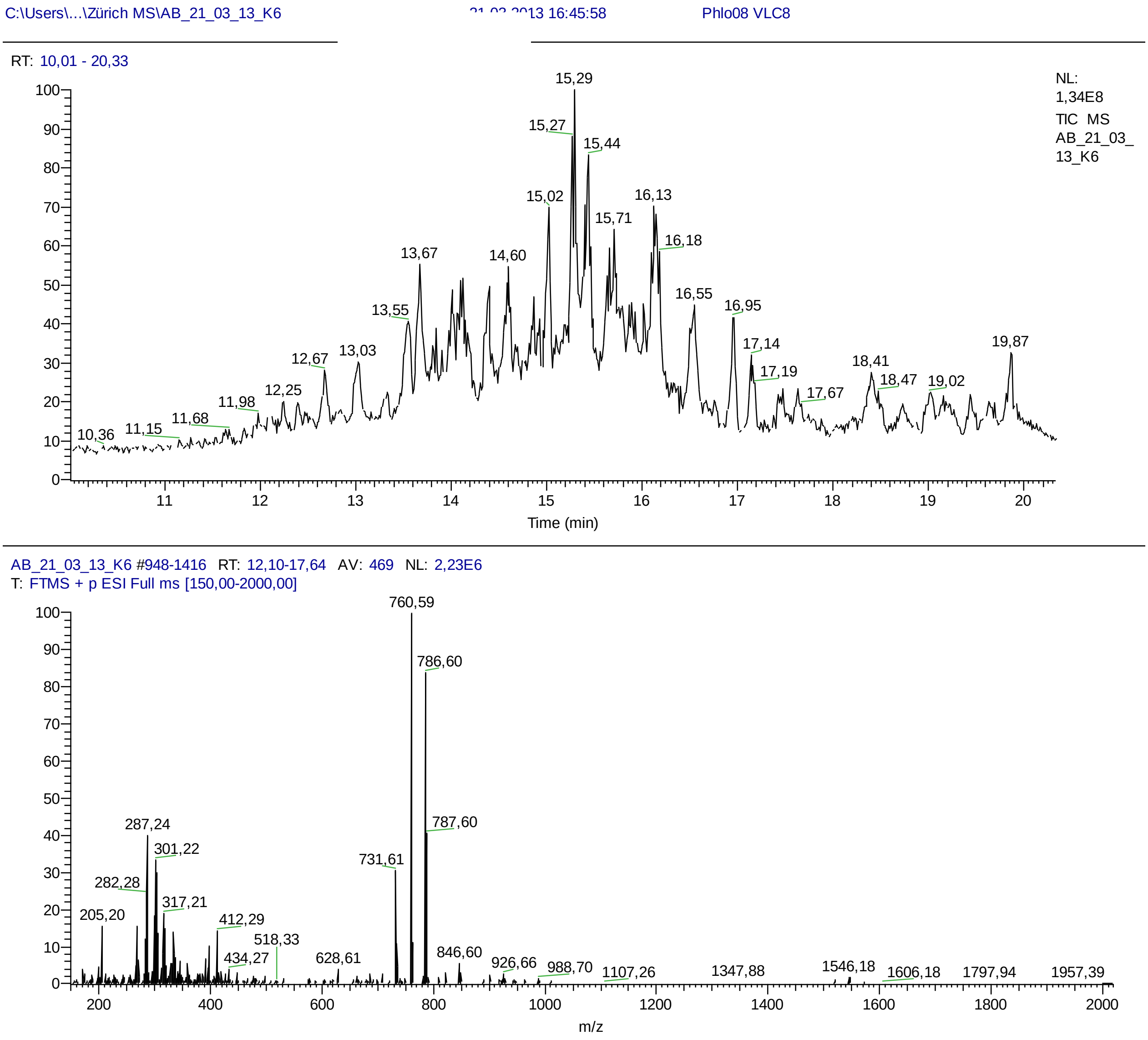

Figure S52. UPLC-HRMS chromatogram of VLC8

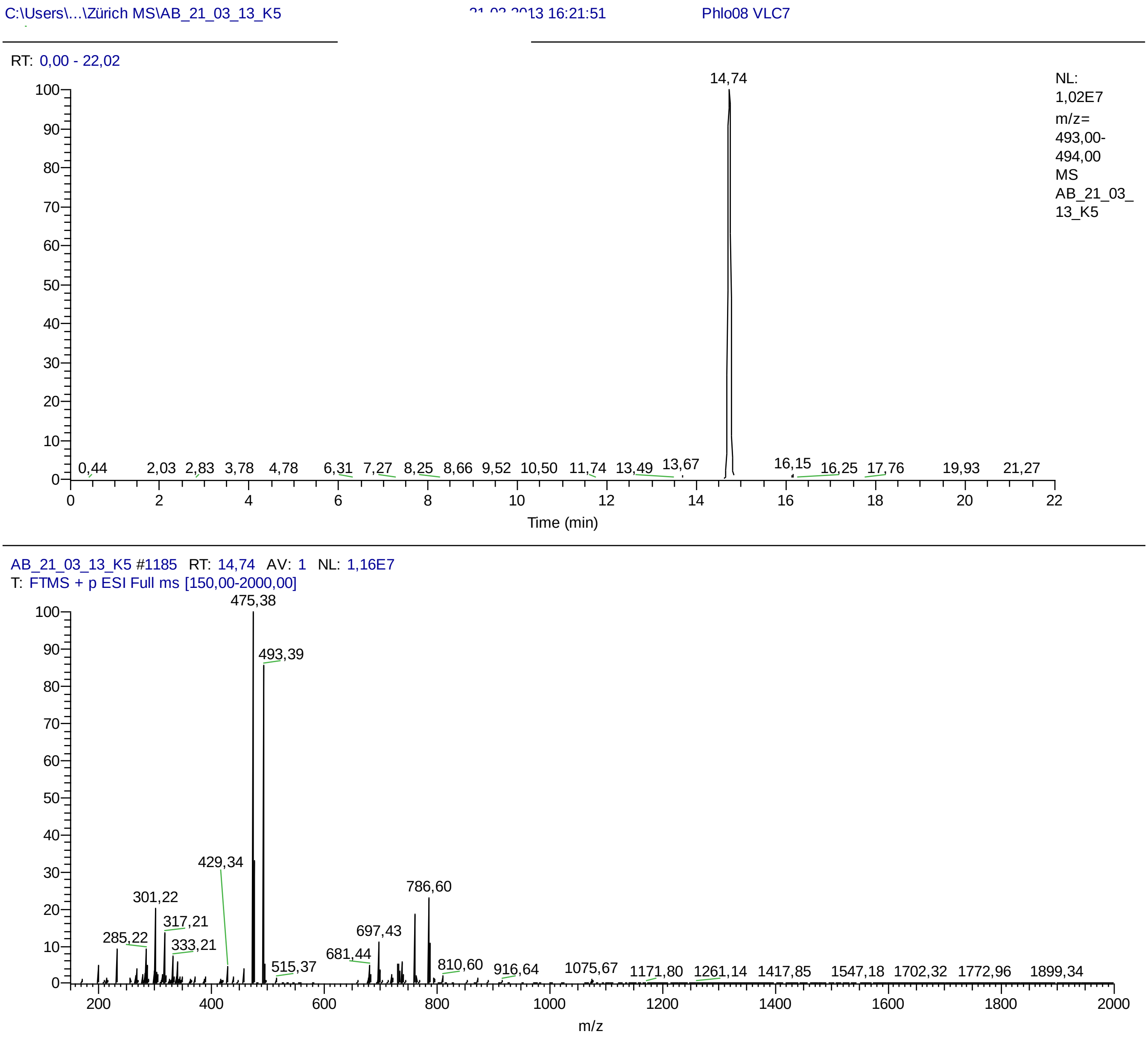

Figure S53. Abundance of 3β,5α,6β-trihydroxy-9-oxo-9,11-secogorgostan-11-ol (**1**) (*m/z* 493.39 M+H) in VLC 7

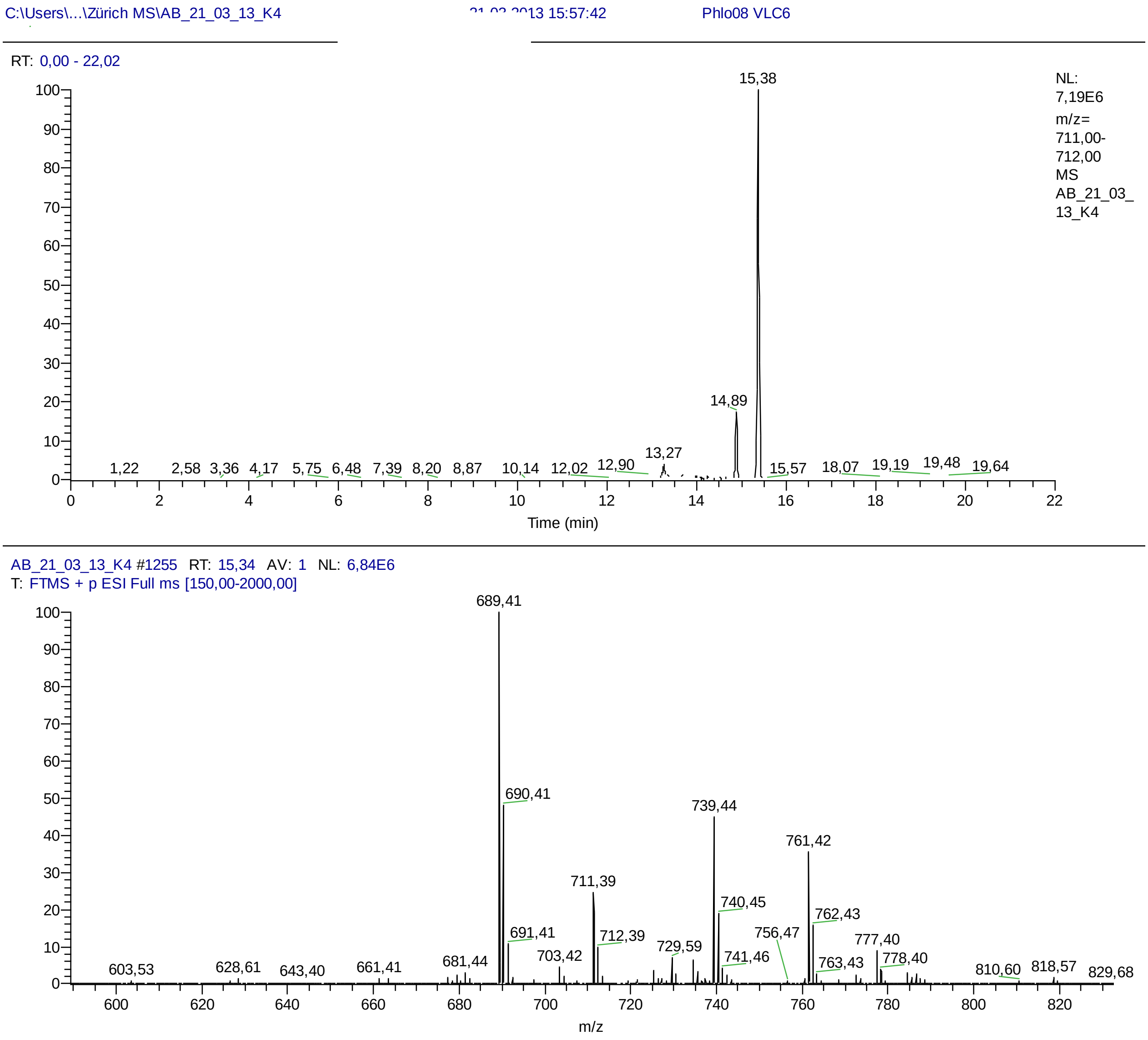

Figure S54. Abundance of *m/z* value of 711.39 in the fraction VLC 6

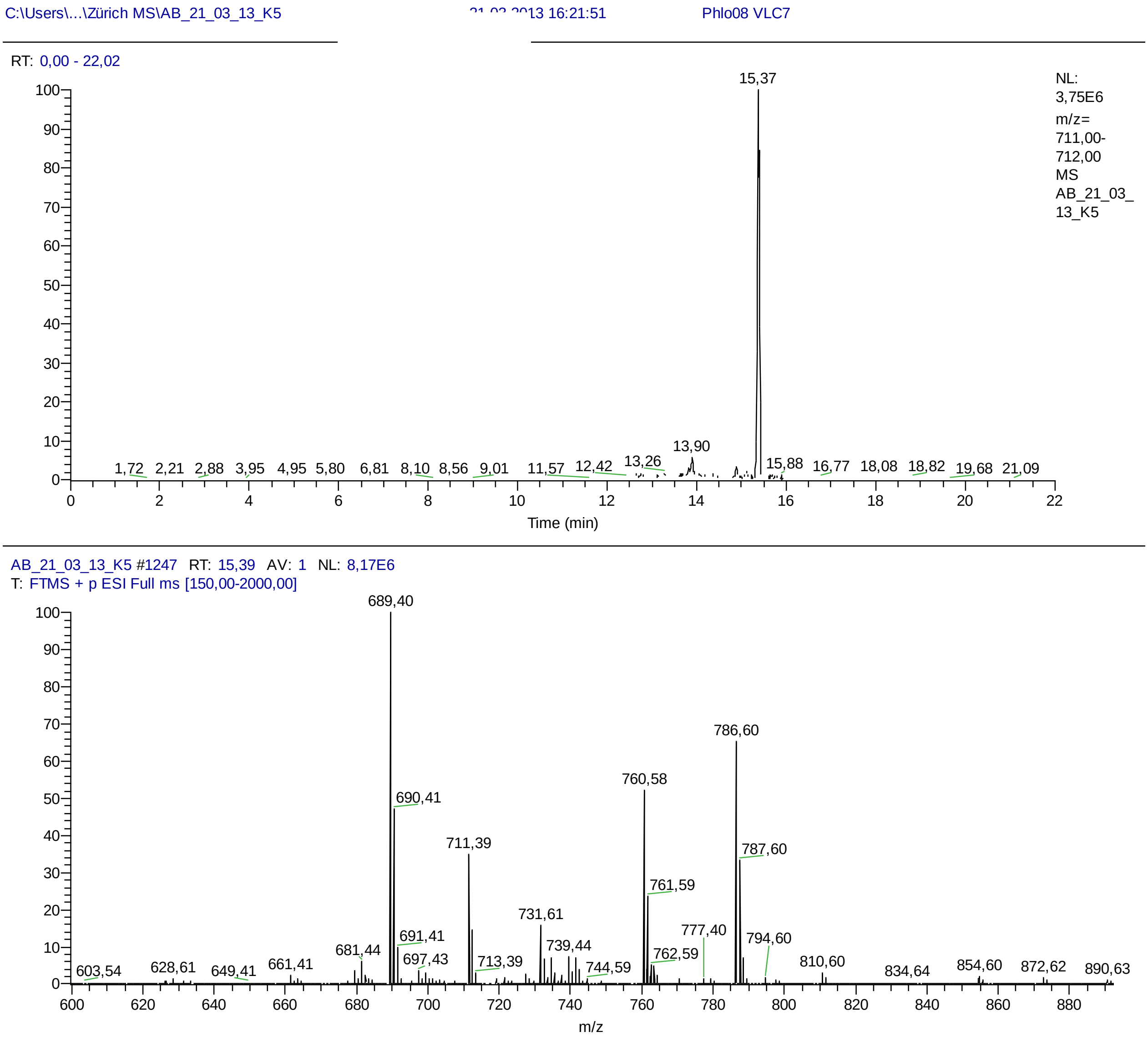

Figure S55. Abundance of *m/z* value of 711.39 in the fraction VLC 7

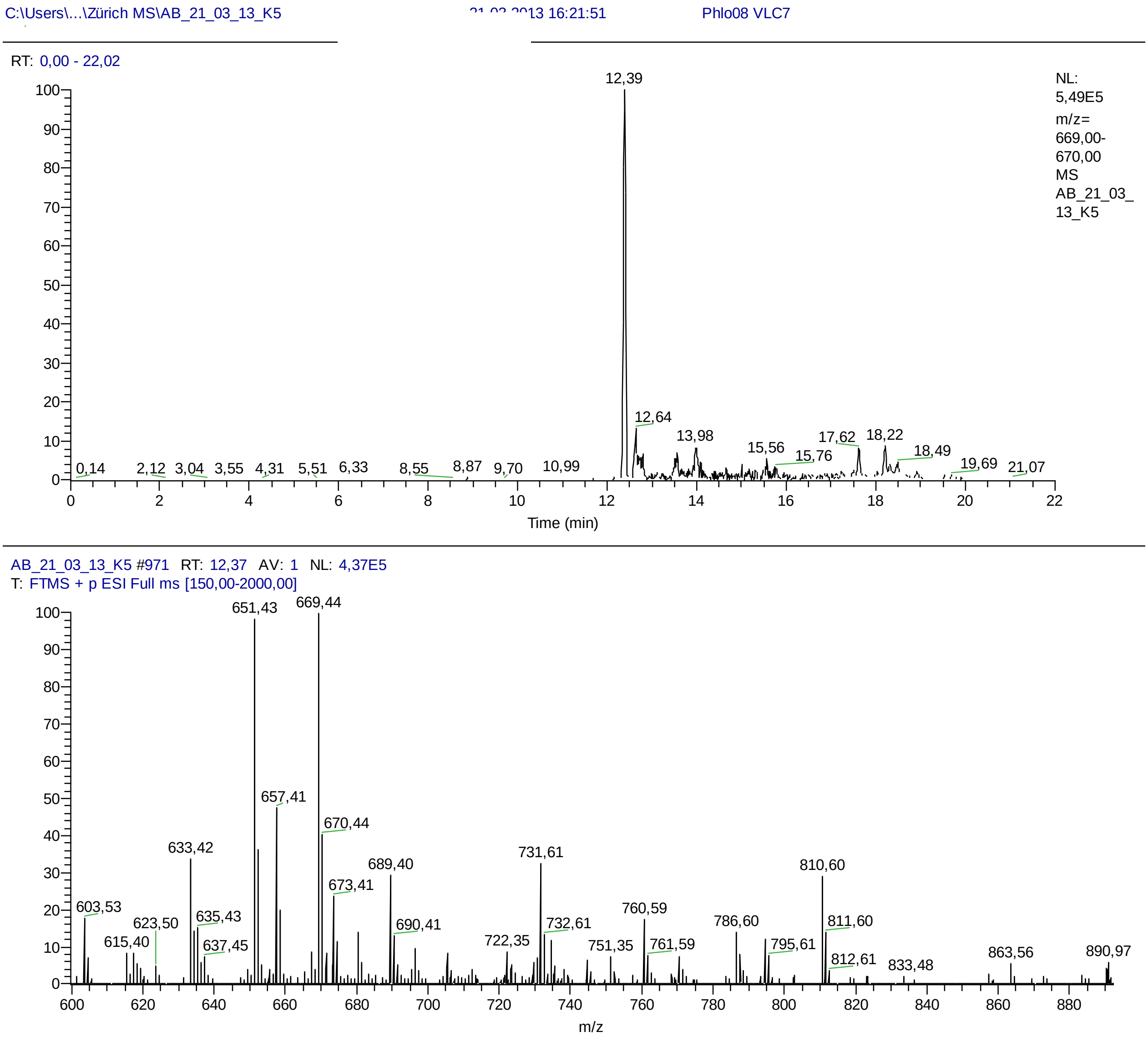

Figure S56. Abundance of *m/z* value of 669.44 in the fraction VLC 7

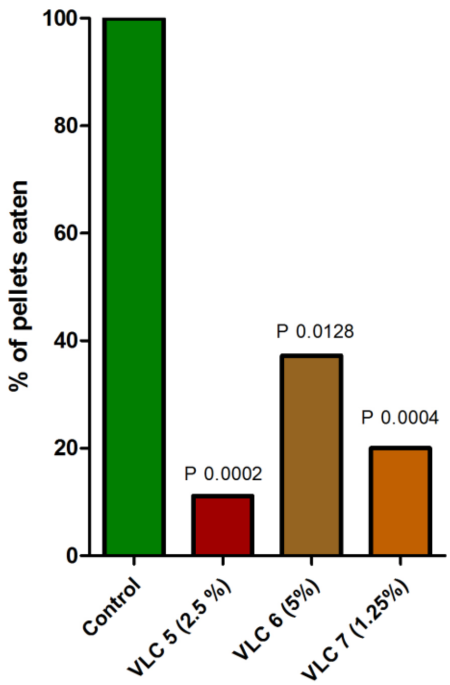

Figure S57. Preliminary results of feeding deterrence assay.
Effect of fractions obtained by vacuum liquid chromatography (VLC 5, 6 and 7) on predation by *Canthigaster solandri* (*n* 8–11) at different concentrations (% of pellet dry mass). Fishers exact test was used, P values indicate a significant feeding deterrence (all $P < 0.05$).
